# Hypochlorite-Activated Fluorescence Emission and Antibacterial Activities of Imidazole Derivatives for Biological Applications

**DOI:** 10.3389/fchem.2021.713078

**Published:** 2021-07-12

**Authors:** Thanh Chung Pham, Van-Nghia Nguyen, Yeonghwan Choi, Dongwon Kim, Ok-Sang Jung, Dong Joon Lee, Hak Jun Kim, Myung Won Lee, Juyoung Yoon, Hwan Myung Kim, Songyi Lee

**Affiliations:** ^1^ Industry 4.0 Convergence Bionics Engineering, Pukyong National University, Busan, South Korea; ^2^ Department of Chemistry and Nanoscience, Ewha Womans University, Seoul, South Korea; ^3^ Department of Chemistry, Pusan National University, Busan, South Korea; ^4^ Department of Energy Systems Research, Ajou University, Suwon, South Korea; ^5^ Department of Chemistry, Pukyong National University, Busan, South Korea; ^6^ Department of Chemistry, Ajou University, Suwon, South Korea

**Keywords:** fluorescent sensor, fluorogenic probe, hypochlorite sensor, antibacterial effect, probe–killer

## Abstract

The ability to detect hypochlorite (HOCl/ClO^−^) *in vivo* is of great importance to identify and visualize infection. Here, we report the use of imidazoline-2-thione (**R**_**1**_ **SR**_**2**_) probes, which act to both sense ClO^−^ and kill bacteria. The N_2_C=S moieties can recognize ClO^−^ among various typical reactive oxygen species (ROS) and turn into imidazolium moieties (**R**_**1**_ **IR**_**2**_) *via* desulfurization. This was observed through UV–vis absorption and fluorescence emission spectroscopy, with a high fluorescence emission quantum yield (Փ_F_ = 43–99%) and large Stokes shift (∆v∼115 nm). Furthermore, the **DIM** probe, which was prepared by treating the **DSM** probe with ClO^−^, also displayed antibacterial efficacy toward not only *Escherichia coli* (*E. coli*) and *Staphylococcus aureus* (*S. aureus*) but also methicillin-resistant *Staphylococcus aureus* (MRSA) and extended-spectrum ß-lactamase–producing *Escherichia coli* (ESBL-EC), that is, antibiotic-resistant bacteria. These results suggest that the **DSM** probe has great potential to carry out the dual roles of a fluorogenic probe and killer of bacteria.

## Introduction

Invasion of microorganisms such as bacteria and viruses can cause infectious diseases. Due to the worldwide increase in cases of severe bacterial diseases, scientists have attempted to develop technologies that serve as both fluorogenic probes for the identification of infection and antibacterial agents. Unfortunately, the continued overuse of antibiotics coupled with the rapid spread of resistance mechanisms has rendered many antibiotics inactive (Kardas et al., 2005). As a result, new pathogens have come into being that are multidrug resistant (MDR), such as methicillin-resistant *Staphylococcus aureus* (MRSA) and extended-spectrum ß-lactamase–producing *Escherichia coli* (ESBL-EC), which have undermined most clinically useful antibiotics. Therefore, the emergence of drug-resistant bacteria is one of the growing challenges to anti-infection therapy. At the same time, emerging infectious diseases need very urgent and immediate treatment due to their rapid spread. In this regard, theragnostics, a treatment strategy that combines therapeutics with diagnostics, could be embraced by clinicians and patients (Pene et al., 2009). In recent years, material-based approaches have found preliminary use for the treatment of bacterial infections (Kurapati et al., 2016; Gupta et al., 2019) and for the image-guided treatment of bacterial infections (Kim et al., 2018; Lee S. et al., 2020). Such methods have provided an approach that can produce the desired therapeutic effect with a reduced potential to develop drug-resistant bacteria (Lee et al., 2016; Li et al., 2018a; Li et al., 2018b; Li et al., 2019). Among the various reactive oxygen species (ROS), hypochlorite (HOCl/ClO^−^) acts as a powerful microbicidal agent in the innate immune system. ClO^−^ is mainly produced by the myeloperoxidase (MPO)-catalyzed reaction of H_2_O_2_ and Cl^−^ in immunocytes (Domigan et al., 1995; Xu et al., 2013; Pak et al., 2018; Wu et al., 2019; Nguyen et al., 2020). The regulated production of microbicidal HOCl is required for the host to control invading microbes. On the other hand, OCl^−^ reacts rapidly with a variety of biomolecules and is connected with various disorders (Winterbourn et al., 2000; Krasowska and Konat, 2004; Jeitner et al., 2005).

A fundamentally important yet challenging feature of studies in this area is the design of new chemorecognition processes. Imidazolium salts with good water solubility and stability have been used as fluorescence sensors in aqueous solution (Xu et al., 2010; Kim et al., 2012; Xu et al., 2015). To take advantage of the properties of imidazolium salts, we designed imidazoline-2-thione (**R**_**1**_ **SR**_**2**_) probes to be ClO^−^ fluorescent probes capable of inducing bacterial growth inhibition. Furthermore, the photophysical properties of **R**_**1**_ **IR**_**2**_ and **R**_**1**_ **SR**_**2**_ were examined *via* not only experimental results but also time-dependent DFT (TD-DFT) calculation. Bacterial growth was significantly reduced by the imidazolium moieties (**R**_**1**_ **IR**_**2**_) that were generated by the treatment of **R**_**1**_ **SR**_**2**_ with ClO^−^. Among the pairs of **R**_**1**_ **IR**_**2**_ and **R**_**1**_ **SR**_**2**_, the **DSM** probe showed excellent selectivity and sensitivity toward ClO^−^. Also, the **DIM** probe showed antibacterial efficacy toward not only *E.* c*oli* and *S. aureus* but also methicillin-resistant *S. aureus* (MRSA) and extended-spectrum ß-lactamase–producing *E. coli* (ESBL-EC). The **DIM** probe initially induces electrostatic interactions between the cationic imidazolium salts and the negatively charged bacterial surface, followed by structural perturbation, resulting in bacterial cell death. Similar membrane disruption through interactions of cationic imidazolium groups has been suggested by previous reports (Riduan and Zhang, 2013). Overall, this report demonstrates the importance and benefits of the new fluorogenic probe **DSM** for anti-pathogenic diagnostic and therapeutic applications.

## Experimental Design

For the synthesis of **R**_**1**_ **SR**_**2**_, a mixture of **R**_**1**_ **IR**_**2**_ (0.1 mmol), sulfur (1.0 mmol), and sodium methoxide (1.0 mmol) in anhydrous methanol (20 ml) was stirred overnight at room temperature. After the solvent was removed, the crude product was dissolved in DW and extracted by MC 3 times. The organic phase was collected and dried over Na_2_SO_4_. It was purified by silica gel column chromatography, using H/MC (9/1) as the eluent to get a white solid as the product (yield ∼90%).

**BSB**: ^1^H NMR (400 MHz, chloroform-*d*) δ 7.77 (dd, *J* = 6.3, 3.3 Hz, 2H), 7.69–7.61 (m, 2H), 7.41–7.32 (m, 4H), 7.20–7.09 (m, 4H), 6.97–6.90 (m, 2H), 5.80 (s, 4H). ^13^C NMR (101 MHz, chloroform-*d*) δ 174.85, 134.28, 133.18, 131.93, 130.55, 129.36, 128.02, 127.84, 127.82, 125.25, 122.77, 105.92, 48.67. ESI HRMS m/z = 536.9630 [M + H]^+^, calc. for C_25_H_18_Br_2_N_2_S = 535.96.

**BSM**: ^1^H NMR (400 MHz, chloroform-*d*) δ 7.92–7.85 (m, 1H), 7.81–7.73 (m, 1H), 7.67–7.59 (m, 1H), 7.53 (s, 1H), 7.40 (pd, *J* = 6.8, 1.6 Hz, 2H), 7.31 (s, 1H), 7.16–7.06 (m, 2H), 6.89–6.81 (m, 1H), 5.73 (s, 2H), 3.93 (d, *J* = 0.6 Hz, 3H). ^13^C NMR (101 MHz, chloroform-*d*) δ 174.26, 134.36, 133.07, 132.92, 131.88, 130.53, 130.46, 129.24, 127.94, 127.86, 127.83, 127.70, 125.21, 125.09, 122.67, 105.70, 105.04, 48.35, 31.68. ESI HRMS m/z = 383.0212 [M + H]^+^, calc. for C_19_H_15_BrN_2_S = 382.01.

**CSB**: ^1^H NMR (400 MHz, chloroform-*d*) δ 8.04 (dt, *J* = 7.8, 0.9 Hz, 2H), 7.75 (d, *J* = 7.5 Hz, 2H), 7.62 (dd, *J* = 7.7, 1.6 Hz, 1H), 7.48–7.33 (m, 6H), 7.24–7.17 (m, 4H), 7.08 (dtd, *J* = 16.7, 7.4, 1.7 Hz, 2H), 6.82–6.75 (m, 1H), 5.69 (s, 2H), 4.42 (td, *J* = 6.8, 2.1 Hz, 4H), 2.06 (dq, *J* = 31.4, 7.4 Hz, 4H). ^13^C NMR (101 MHz, chloroform-*d*) δ 140.43, 133.08, 129.23, 127.94, 127.75, 125.82, 125.11, 122.99, 120.51, 119.03, 108.77, 105.76, 105.04, 77.42, 77.10, 76.78, 48.33, 44.73, 42.65. ESI HRMS m/z = 612.1080 [M + Na]^+^, calc. for C_34_H_28_BrN_3_S = 589.12.

**CSC**: ^1^H NMR (400 MHz, chloroform-*d*) δ 8.03 (dt, *J* = 7.9, 1.0 Hz, 4H), 7.74 (dd, *J* = 6.3, 3.3 Hz, 2H), 7.46–7.35 (m, 10H), 7.23–7.13 (m, 6H), 4.34 (dt, *J* = 18.7, 6.8 Hz, 8H), 2.10–2.00 (m, 4H), 1.94 (q, *J* = 7.2 Hz, 4H), 1.30–1.20 (m, 4H), 0.90–0.79 (m, 4H). ^13^C NMR (101 MHz, chloroform-*d*) δ 172.90, 140.42, 131.88, 130.15, 127.69, 125.78, 124.96, 122.97, 120.49, 119.00, 108.77, 104.90, 77.42, 77.11, 76.79, 44.41, 42.64, 26.09, 25.31. ESI HRMS m/z = 665.2709 [M + Na]^+^, calc. for C_43_H_38_N_4_S = 642.28.

**CSD**: ^1^H NMR (600 MHz, chloroform-*d*) δ 8.03 (dt, *J* = 7.7, 1.0 Hz, 2H), 7.79 (d, *J* = 1.9 Hz, 1H), 7.75 (ddd, *J* = 8.1, 2.2, 1.2 Hz, 2H), 7.45–7.37 (m, 6H), 7.23–7.16 (m, 5H), 6.67–6.64 (m, 1H), 5.61 (s, 2H), 4.41 (q, *J* = 7.0 Hz, 4H), 2.14–1.96 (m, 4H). ^13^C NMR (101 MHz, chloroform-*d*) δ 173.81, 140.41, 135.38, 133.57, 131.64, 131.14, 130.29, 128.96, 127.78, 127.72, 125.82, 125.22, 123.16, 122.99, 121.99, 120.52, 119.05, 108.74, 105.56, 105.20, 77.42, 77.11, 76.79, 47.86, 44.76, 42.64, 26.05, 25.29. ESI HRMS m/z = 690.0185 [M + Na]^+^, calc. for C_34_H_27_Br_2_N_3_S = 667.03.

**CSM**: ^1^H NMR (400 MHz, chloroform-*d*) δ 8.04 (dt, *J* = 7.7, 1.0 Hz, 2H), 7.74 (dd, *J* = 6.6, 3.0 Hz, 2H), 7.62 (dd, *J* = 7.7, 1.5 Hz, 1H), 7.48–7.33 (m, 6H), 7.26 (s, 7H), 7.24–7.15 (m, 4H), 7.08 (dtd, *J* = 16.7, 7.4, 1.7 Hz, 2H), 6.78 (dd, *J* = 7.4, 1.9 Hz, 1H), 5.69 (s, 2H), 4.42 (dd, *J* = 7.3, 5.7 Hz, 4H), 2.10 (p, *J* = 6.8 Hz, 2H), 2.01 (p, *J* = 7.0 Hz, 2H). ^13^C NMR (101 MHz, chloroform-*d*) δ 173.25, 140.43, 132.81, 131.89, 130.28, 127.79, 127.63, 125.78, 125.03, 124.94, 122.96, 120.48, 119.00, 108.78, 104.91, 104.80, 44.47, 42.65, 31.31, 26.12, 25.38. ESI HRMS m/z = 458.1661 [M + Na]^+^, calc. for C_28_H_25_N_3_S = 435.18.

**DSB**: ^1^H NMR (400 MHz, chloroform-*d*) δ 7.84–7.73 (m, 3H), 7.69–7.60 (m, 1H), 7.43–7.34 (m, 3H), 7.33 (s, 1H), 7.31–7.22 (m, 3H), 7.20–7.09 (m, 2H), 6.96–6.89 (m, 1H), 6.82 (dd, *J* = 8.3, 0.8 Hz, 1H), 5.79 (s, 2H), 5.73 (s, 2H). ^13^C NMR (101 MHz, chloroform-*d*) δ 174.80, 135.49, 134.20, 133.54, 133.21, 131.87, 131.74, 131.22, 130.60, 130.54, 129.41, 129.08, 128.02, 127.84, 127.79, 125.38, 123.27, 122.78, 122.13, 106.09, 105.72, 48.70, 48.21. ESI HRMS m/z = 614.8735 [M + H]^+^, calc. for C_25_H_17_Br_3_N_2_S = 613.87.

**DSD**: ^1^H NMR (400 MHz, chloroform-*d*) δ 7.84–7.74 (m, 4H), 7.40 (dd, *J* = 6.3, 3.2 Hz, 2H), 7.34 (s, 2H), 7.28 (dd, *J* = 8.3, 1.9 Hz, 2H), 6.80 (dd, *J* = 8.3, 0.7 Hz, 2H), 5.72 (s, 4H). ^13^C NMR (101 MHz, chloroform-*d*) δ 174.73, 135.53, 133.44, 131.67, 131.22, 130.59, 129.05, 127.82, 125.50, 123.29, 122.20, 105.89, 48.24. ESI HRMS m/z = 692.7840 [M + H]^+^, calc. for C_25_H_16_Br_4_N_2_S = 691.78.

**DSM**: ^1^H NMR (400 MHz, chloroform-*d*) δ 7.93–7.85 (m, 1H), 7.79 (dd, *J* = 7.5, 2.0 Hz, 2H), 7.53 (s, 1H), 7.48–7.36 (m, 2H), 7.28 (s, 1H), 7.22 (d, *J* = 2.0 Hz, 3H), 6.74 (dd, *J* = 8.3, 0.8 Hz, 1H), 5.66 (s, 2H), 3.92 (s, 3H). ^13^C NMR (101 MHz, chloroform-*d*) δ 174.15, 135.38, 133.61, 132.83, 131.65, 131.12, 130.56, 130.44, 129.07, 127.84, 127.73, 125.34, 125.24, 123.19, 122.01, 105.51, 105.22, 47.89, 31.71. ESI HRMS m/z = 460.9317 [M + H]^+^, m/z = 482.9137 [M+Na]^+^ calc. for C_19_H_15_Br_2_N_2_S = 459.93.

## Results and Discussion

### Molecular Design, Synthesis, and Characterization

**SCHEME 1:**
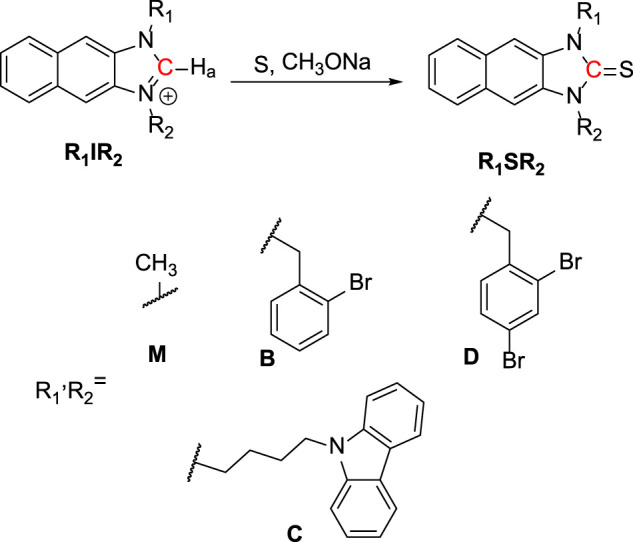
Synthesis of imidazoline-2-thiones (**R**_**1**_ **SR**_**2**_).

**FIGURE 1:**
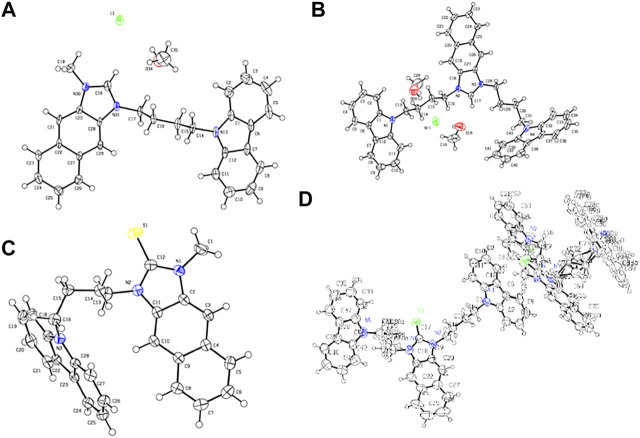
Crystal structures of **(A) CIM**, **(B) CIC**, **(C) CSM**, and **(D) CSC**.

As shown in Scheme 1, **R**_**1**_ **IR**_**2**_ was synthesized from 2,3-Diaminonaphthalene in a 3-step process as follows: imidazole cyclization, alkylation, and imidazolium salt formation. Several bromide and carbazole derivatives have shown antibacterial activity (Yaqub et al., 2013; Gottardi et al., 2014; Bashir et al., 2015; Liu et al., 2015; Salih et al., 2016; Popescu et al., 2021). Thus, the introduction of bromobenzyl, dibromobenzyl, and carbazole groups is expected to increase the antibacterial effect of the probes. Then, the **R**_**1**_ **IR**_**2**_ salt was treated with sulfur and CH_3_ONa as a catalyst in ACN, leading to the formation of the corresponding molecule **R**_**1**_ **SR**_**2**_. All synthetic processes and collected structures are detailed in the experimental section and the supporting information. Several products were characterized not only by ^1^H NMR, ^1^C NMR, and HRMS spectra (Supplementary Material) but also by crystallization structures (Figure 1; Supplementary Figures S37, S38; Supplementary Tables S1, S2), which have not been reported in previous studies on similar N_2_C=S structures (Xu et al., 2015; Xu et al., 2016). In particular, the length of the thioketone in the N_2_C=S type was found to be 1.657–1.671 Å (Table 1), which is longer than that in other thioketone types such as thiobenzophenone, thioformaldehyde, thioacetone, etc. (∼1.63–1.64 Å) due to the presence of C–N π-bonds (Mullen and Hellner, 1978; Allen et al., 1987). Thus, the N_2_C=S bond can make the compound more active and sensitive toward ROS/RNS.

**TABLE 1:** Bond lengths between the crystal and optimized structures of **CIM**, **CSM**, **CIC**, and **CSC**.

Bond	CIM		CSM		CIC		CSC	
	Crystal	Optimized	Crystal	Optimized	Crystal	Optimized	Crystal	Optimized
C=S	—	—	1.671 Å	1.669 Å	—	—	1.657 Å	1.670 Å
C-N/C=N	1.332	1.333 Å	1.367 Å	1.381 Å	1.329 Å	1.334 Å	1.383 Å	1.380 Å
C-H_a_	0.950	1.079 Å	—	—	0.950 Å	1.079 Å	—	—

— does not exist.

**FIGURE 2:**
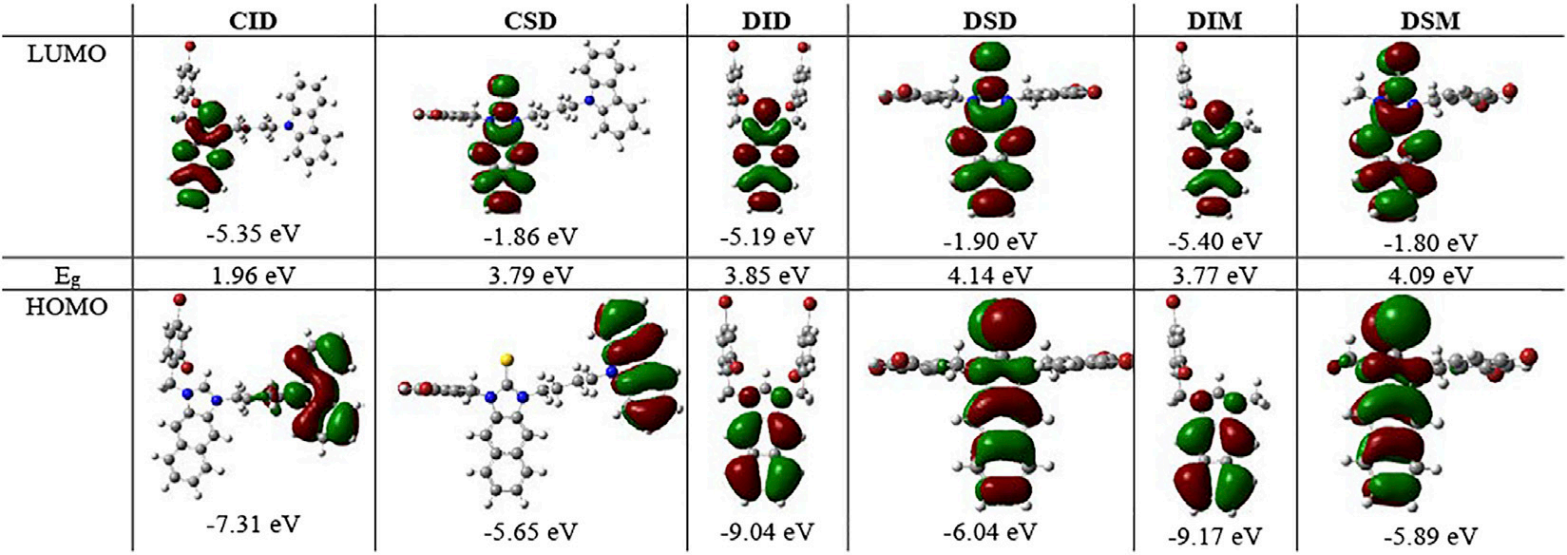
Molecular orbitals and associated energies of **CID** and **CSD**, **DID** and **DSD**, **DIM** and **DSM**, and the energy gap (E_g_) between the HOMO and the LUMO.

To better understand not only the molecular structures but also the molecular orbitals and energy levels of **R**_**1**_ **IR**_**2**_ and **R**_**1**_ **SR**_**2**_ **,** geometrical optimization was performed through theoretical DFT calculations in the Gaussian 09 program package using the B3LVPs functional with the 6-31+g(2d,p) basis set (Pham et al., 2020). The optimized structures without imaginary frequencies were similar to the crystallization structures in terms of several critical bond lengths and angles (Table 1). Their molecular orbitals and energy levels from HOMO+2 to LUMO+2 are shown in Supplementary Tables S1–S5. The HOMO of **CIR**_**2**_ is located in the carbazole moiety, and the LUMO is located in the naphthalene–imidazolium salt center core. However, the HOMO and LUMO of **BIR**_**2**_ and **DIR**_**2**_ are concentrated in the naphthalene–imidazolium salt core (Figure 2). Similarly, the HOMO of **CSR**_**2**_ is located in the carbazole moiety, whereas the HOMO and LUMO of **BSR**_**2**_ and **DSR**_**2**_ are located in the naphthalene and imidazoline-2-thione moieties. The difference originates from the introduction of the carbazole moiety, which is known as a strong donor and fluorescence quencher (Ledwon, 2019; Rehmat et al., 2020; Saritha et al., 2020). Thus, the energy gap between the LUMO and the HOMO (E_g_) of **CIR**_**2**_ (1.80–2.03 eV) is significantly lower than those of **BIR**_**2**_ and **DIR**_**2**_ (3.77–3.86 eV). The E_g_ of **CSR**_**2**_ (3.79–3.86 eV) is lower than those of **BIR**_**2**_ and **DIR**_**2**_ (4.08–4.14 eV). The reduction in difference is assigned to the strong electron acceptor ability of imidazolium salts, which enhances electron transfer from the carbazole electron donor to the imidazolium salt electron acceptor (vs. from the carbazole to the naphthalene and imidazoline-2-thione moieties).

### Photophysical Properties and Theoretical Calculations

**FIGURE 3:**
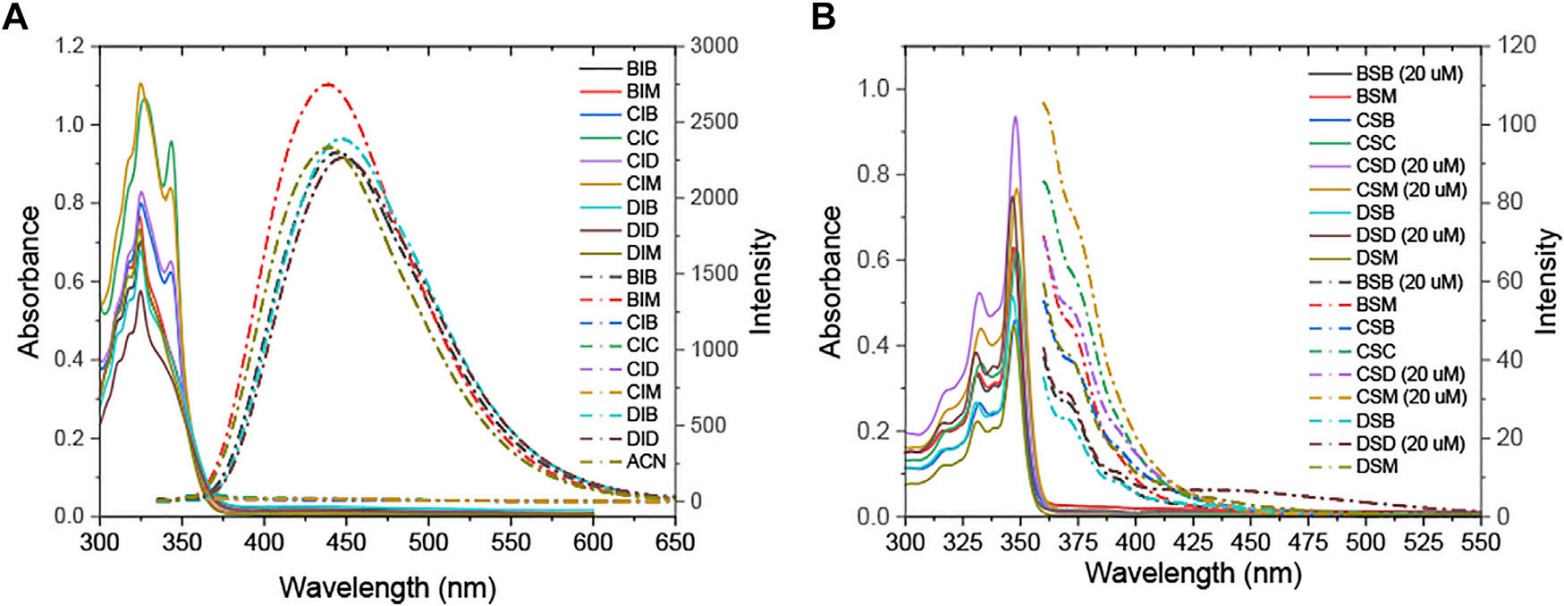
UV–vis (solid line) and fluorescence emission (dashed/dotted line) spectra of **(A) R**_**1**_ **IR**_**2**_ (80 µM) and **(B) R**_**1**_ **SR**_**2**_ (10 µM) (in ACN).

The UV–vis absorption and fluorescence emission spectroscopy of **R**_**1**_ **IR**_**2**_ and **R**_**1**_ **SR**_**2**_ were examined in various solvents (Supplementary Figures S39–S47). At the same time, time-dependent DFT (TD-DFT) calculations were carried out in the optimized structures using a hybrid functional method, a gradient-corrected method, and a popular local method (Adamo and Jacquemin, 2013; Pham et al., 2021a) to better understand their photophysical properties. The optical excitation energies of **R**_**1**_ **IR**_**2**_ and **R**_**1**_ **SR**_**2**_ were determined using the CAM-B3LYP functional with the Def-2-TZVP basis set and the TPSSTPSS functional with the 6-31+G (2 days, p) basis set, respectively. The results corresponded well to the experimental data. **R**_**1**_ **SR**_**2**_ showed absorption peaks at approximately 350 nm, with a high molar absorption coefficient (ɛ = 31.4–62.8 × 10^3^) and weak emission (Փ_F_ = 0.1–1.8%) (Figure 3B; Table 2). On the other hand, **R**_**1**_ **IR**_**2**_ exhibited absorption peaks at about 325 nm, with a lower molar absorption coefficient (ε = 7.2–13.3 ×10^3^) (Figure 3A; Table 2). **R**_**1**_ **IR**_**2**_ without a carbazole moiety showed a strong emission peak at approximately 440 nm (Փ_F_ = 26–63%) and a large Stokes shift (∆v ∼ 115 nm). In sharp contrast, **R**_**1**_ **IR**_**2**_ with a carbazole moiety exhibited weak emission (Փ_F_ = 3.8–8.2%) due to photoinduced electron transfer (PET) from the carbazole donor to the naphthalene–imidazolium salt acceptor (Sun et al., 2019). The absorption of **R**_**1**_ **IR**_**2**_ is assigned to the S_0_ → S_1_ transition and its emission is assigned to the S_1_′ → S_0_ transition, with the orbital contribution located in the naphthalene–imidazolium salt core (Supplementary Table S12). **R**_**1**_ **SR**_**2**_ exhibited S_0_ → S_3_ absorption and S_2_′ → S_1_ emission in the absence of carbazole groups, while it showed S_0_ ↔ S_n_/S_n_′ transition at the higher level of S_n_/S_n_′ in the presence of carbazole groups. The absorption band is contributed by other orbitals in addition to the HOMO and the LUMO (Supplementary Table S13), but natural transition orbitals (NTOs) showed a similar electronic transition in the naphthalene and imidazoline-2-thione moieties of **R**_**1**_ **SR**_**2**_ (Supplementary Table S14), which demonstrates that the two have the same UV/vis absorption spectra (Figure 3B).

**TABLE 2:** Photophysical properties of **R**_**1**_ **IR**_**2**_ and **R**_**1**_ **SR**_**2**_ according to ACN and computational calculations.

	*λ*_abs_ (nm)	ε (× 10^3^)	*λ*_ems_ (nm)	∆v (nm)	Փ_F_ (%)	E_g_ (eV)	Absorption (S_0_ → S_n_)				Fluorescence (S_n_→S_0_)		
							S_n_	Orbital contribution	∆E (eV)	f	S_n_	∆E (eV)	f
**BIB**	325	8.77	445	120	32.6	3.86	S_1_	[H] → [L]: 96.3%	3.954	0.12	S_1_′	3.463	0.22
**BSB**	347	31.42	—	—	0.8	4.12	S_3_	[H-1] → [L]: 75.5%	3.552	0.39	S_2_′	3.393	0.58
**BIM**	324	9.59	439	115	59.2	3.79	S_1_	[H]→[L]: 96.4%	3.955	0.12	S_1_′	3.508	0.23
**BSM**	348	62.75	—	—	0.9	4.08	S_3_	[H-1] → [L]: 81.9%	3.539	0.28	S_2_′	3.375	0.57
**CIB**	325	9.99	370	45	3.8	2.03	S_1_	[H]→[L]: 95.9%	3.942	0.13	S_1_′	3.482	0.23
**CSB**	353	54.45	—	—	1.1	3.84	S_6_	[H-2] → [L]: 60.1%	3.538	0.28	S_5_′	3.495	0.55
**CIC**	328	13.32	370	42	8.2	1.85	S_1_	[H-4] → [L]: 95.5%	3.932	0.16	S_1_′	3.335	0.22
**CSC**	349	62.16	—	—	0.9	3.81	S_11_	[H-3] → [L]: 61.0%	3.519	0.30	S_6_′	3.461	0.36
**CID**	325	10.36	370	45	5.1	1.96	S_1_	[H-2] → [L]: 95.8%	3.936	0.13	S_1_′	3.469	0.23
**CSD**	348	46.74	—	—	1.4	3.79	S_9_	[H-2] → [L]: 66.7%	3.540	0.27	S_3_′	3.638	0.53
**CIM**	325	13.82	371	46	4.6	1.80	S_1_	[H-2] → [L]: 96.0%	3.946	0.14	S_1_′	3.496	0.23
**CSM**	348	38.32	—	—	1.8	3.86	S_4_	[H-1] → [L]: 56.5%	3.502	0.29	S_4_′	3.451	0.65
**DIB**	325	8.46	446	121	26.4	3.86	S_1_	[H]→[L]: 96.2%	3.953	0.12	S_1_′	3.455	0.22
**DSB**	351	50.78	—	—	0.1	4.13	S_3_	[H-1] → [L]: 71.7%	3.550	0.36	S_2_′	3.401	0.57
**DID**	325	7.19	447	122	26.5	3.85	S_1_	[H]→[L]: 96.1%	3.947	0.12	S_1_′	3.449	0.22
**DSD**	347	37.41	—	—	0.1	4.14	S_3_	[H-1] → [L]: 64.3%	3.553	0.31	S_2_′	3.414	0.56
**DIM**	324	9.17	440	116	62.9	3.77	S_1_	[H]→[L]: 96.3%	3.950	0.12	S_1_′	3.484	0.22
**DSM**	347	44.56	—	—	0.1	4.09	S_3_	[H-1] → [L]: 76.9%	3.541	0.25	S_2_′	3.379	0.56

The molar absorption coefficient (ɛ) (M^−1^ cm^−1^), stock shift (∆v), and fluorescence quantum yield (Փ_F_) were measured in DMSO and toluene for **R**_**1**_ **IR**_**2**_ and **R**_**1**_ **SR**_**2**_, with 9,10-Diphenylanthracene (Փ_F_ = 0.90 in cyclohexane) being used as a reference; the oscillator strength (f), the energy gap (E_g_) between the HOMO and LUMO levels, and the energy gap (∆E) between S_0_ and S_n_/S_n_′ were not observed.

**FIGURE 4:**
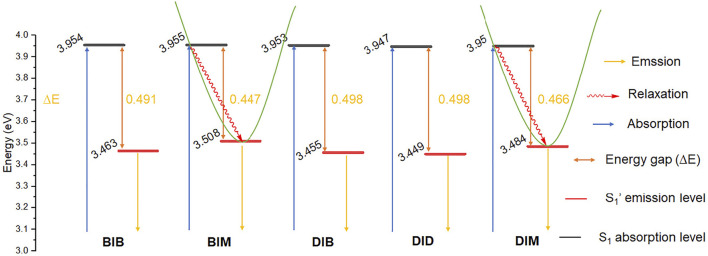
Electronic energy levels diagram of **BIB**, **BIM**, **DIB**, **DID**, and **DIM**.

The fluorescence emission quantum yield of **BIM** and **DIM** (Փ_F_ = 59.2–62.9%) is significantly greater than that of **BIB**, **DIB**, and **DID** (26.4–32.6%) in DMSO, which is attributable to the absence or presence of the second (di)bromobenzyl group. The S_1_ absorption energy of **BIB** and **BIM** vs. **DIB**, **DID**, and **DIM** is similar, whereas the S_1_′ emission energy of **BIM** and **DIM** is higher than that of **BIB** and **DIB** or **DID**, respectively. Thus, the energy gap (∆E) between the S_1_ absorption and the S_1_′ emission of **R**_**1**_ **IM** (R_1_ = **B** or **D**) is lower than that of **R**_**1**_ **IR**_**2**_ (R_1_, R_2_ = **B** or **D**) (Figure 4). Moreover, the energy relaxation wastage of **R**_**1**_ **IM** (R_1_ = **B** or **D**) from the S_1_ absorption level to the S_1_′ emission level is less than that of **R**_**1**_ **IR**_**2**_ (**R**_**1**_, **R**_**2**_ = **B** or **D**), leading to the increase in fluorescence emission quantum yield of **BIM** and **DIM**.

**FIGURE 5:**
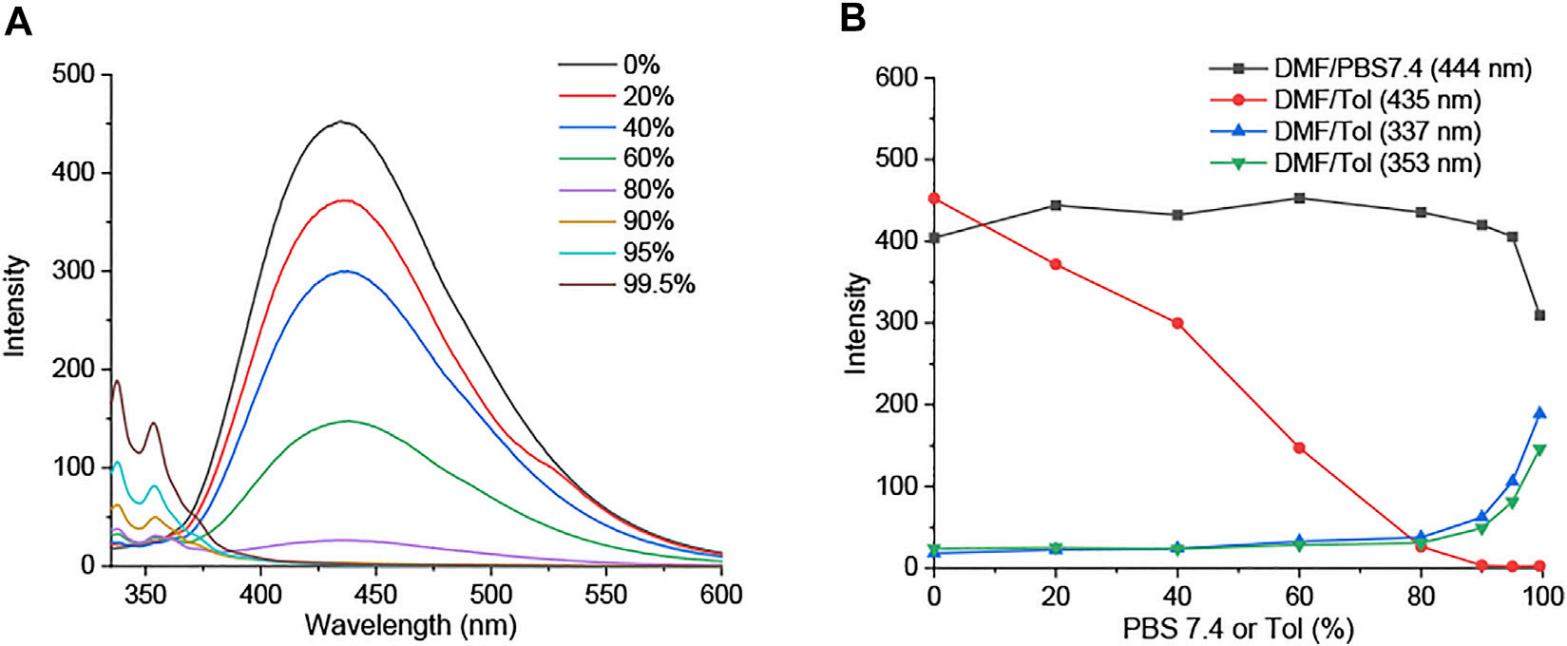
**(A)** Fluorescence emission spectra of **DIM** (5 µM) in DMF/Tol (0–99.5%). **(B)** Fluorescence intensity at emission wavelength of **DIM** (5 µM) in DMF/PBS buffer (pH 7.4) and DMF/Tol (0–99.5%).

At the different volume fractions of PBS buffer with a pH value of 7.4 (0–99.5%), the fluorescence emission of **DIM** and **DID** in DMF is maintained owing to the high water solubility of imidazolium salt groups (Figure 5B; Supplementary Figures S48, S49). We further examined their fluorescence emission in the aggregate state. Interestingly, the emission peak of **DIM** at 450 nm decreased, whereas a 335–350-nm emission band slightly increased with the increasing of toluene (Tol) concentration (0–99.5%) (Figure 5A). **DIM** showed the ACQ effect on its core structure in DMF being quenched in the high concentration of toluene (Supplementary Figure S49); at the same time, it showed blueshift emission assigned from the rotation of bromobenzyl groups around the imidazolium salt core.

### Antibacterial Activity

To compare the antibacterial activity of **R**_**1**_ **IR**_**2**_ and **R**_**1**_ **SR**_**2**_, we calculated the concentration (µM) (CFU_50_) at which the CFU rate equals 50% and the P=CFU_50(R1IR2)_/CFU_50(R1SR2)_ between the imidazolium salt and imidazoline-2-thiones (Table 3). All **R**_**1**_ **SR**_**2**_ showed weak antibacterial ability toward *E.* c*oli*, *S. aureus*, extended-spectrum ß-lactamase–producing *E. coli* (ESBL-EC), *E. coli* expressing green fluorescent protein (EC-GFP), and methicillin-resistant *S. aureus* (MRSA) bacteria with CFU_50_ > 128.0 µM. In sharp contrast, almost all imidazolium salts (**R**_**1**_ **IR**_**2**_) exhibited a stronger antibacterial effect, including against ESBL-EC and MRSA (*p* > 2.4). The antibacterial efficiency of **R**_**1**_ **IR**_**2**_ is quite similar to that of dehydroepiandrosterone-derived (Hryniewicka et al., 2021), peptide-conjugated (Reinhardt et al., 2014), ethoxyether-functionalized (Huang et al., 2011), amino acid–derived (Valls et al., 2020), polydiacetylene-conjugated (Lee et al., 2016), and unsymmetrically substituted (Çoban et al., 2017; Duman et al., 2019) imidazolium salts in previous reports. Furthermore, the antibacterial activity of imidazolium salts (**R**_**1**_ **IR**_**2**_) increased following the substitution of methyl and bromobenzyl with dibromobenzyl groups and the change from one to two dibromobenzyl groups (Table 3).

**TABLE 3:** CFU_50_ (µM) and *p*-value of **R**_**1**_ **IR**_**2**_ and **R**_**1**_ **SR**_**2**_ toward *E. coli*, *S. aureus*, ESBL-EC, EC-GFP, and MRSA bacteria (CFU_50(R1SR2)_ > 128.0 µM).

	*E. coli*		*S. aureus*		ESBL-EC		EC-GFP		MRSA	
	CFU_50_	*p*-value	CFU_50_	*p*-value	CFU_50_	*p*-value	CFU_50_	*p*-value	CFU_50_	*p*-value
BIB	17.5	> 7.3	3.3	> 38.8	26.4	> 4.8	19.8	> 6.5	8.5	> 15.1
BIM	45.3	> 2.8	39.7	> 3.2	53.0	> 2.4	24.9	> 5.1	15.2	> 8.4
CIB	> 128.0	—	2.4	> 53.3	> 128.0	—	21.4	> 6.0	15.7	> 8.2
CIC	> 128.0	—	3.4	> 37.6	> 128.0	—	21.0	> 6.1	15.7	> 8.2
CID	> 128.0	—	1.9	> 67.4	> 128.0	—	15.2	> 8.4	14.6	> 8.8
CIM	31.2	> 4.1	4.2	> 30.5	18.3	> 7.0	11.9	> 10.8	11.7	> 10.9
DIB	9.5	> 13.5	2.1	> 60.1	8.7	> 14.7	22.2	> 5.8	4.1	> 31.2
DID	11.1	> 11.5	2.2	> 58.2	14.0	> 9.1	4.3	> 29.8	5.4	> 23.7
DIM	12.9	> 9.9	6.0	> 21.3	22.1	> 5.8	14.9	> 8.6	14.8	> 8.6

—not calculated.

On the other hand, the incorporation of dibromobenzyl groups enhanced the antibacterial effect toward Gram-positive bacteria such as *S. aureus* and MRSA. The introduction of a carbazole moiety increased the strength of the antibacterial effect toward *S. aureus* (CFU_50_ = 1.9–4.2 µM, *p* > 37.6), whereas the antibacterial ability was moderate toward EC-GFP and MRSA and weak toward *E. coli* and ESBL-EC (CFU_50_ > 128.0 µM). The negative amino group of carbazole affects the antibacterial ability of a positively charged imidazolium salt toward Gram-negative bacteria and enhances the antibacterial ability toward Gram-positive bacteria. In sum, the imidazolium salts **DIM** and **DID** showed strong antibacterial effects (*p* > 5.8) compared to the imidazoline-2-thiones (**R**_**1**_ **SR**_**2**_) **DSM** and **DSD**, respectively. Thus, **DSM** and **DSD** were potentially selected for OFF-ON antibacterial fluorescent probes.

### ClO^−^ Response

**FIGURE 6:**
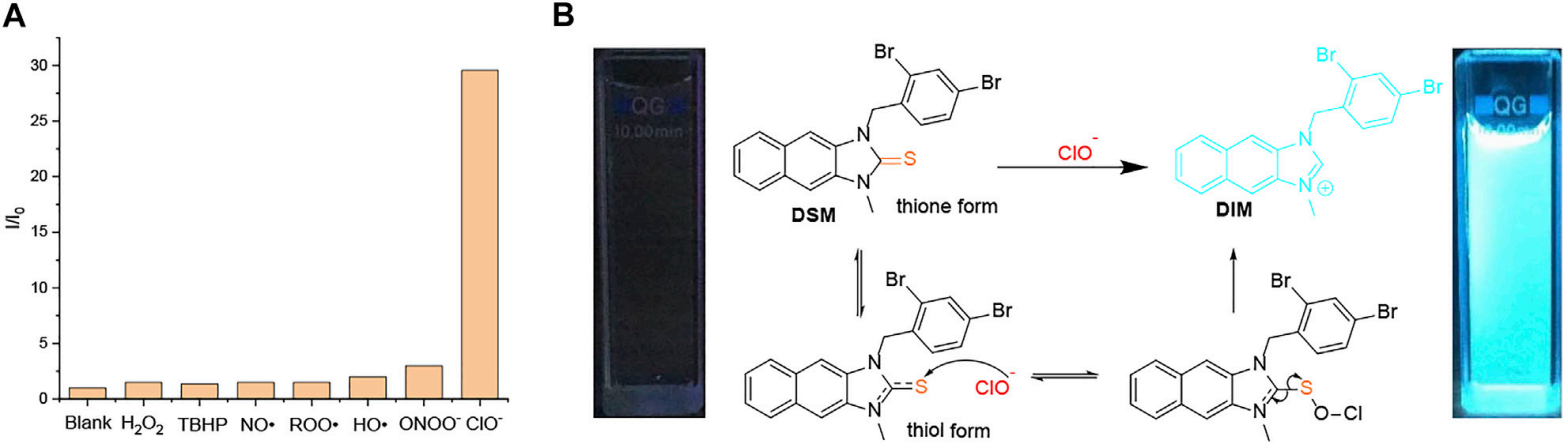
**(A)** Fluorescence intensity ratio (I/I_0_) of **DSM** (5 µM) in PBS buffer with a pH value of 7.4 (0.5% DMF) in the presence of ClO^−^ (50 µM), ROO^•^ (1 mM), NO^•^ (1 mM), H_2_O_2_ (1 mM), TBHP (1 mM), ONOO^−^ (200 µM), and •OH (200 µM). **(B)** Proposed desulfurization mechanism of **DSM** *via* hypochlorite (ClO^−^).

**FIGURE 7:**
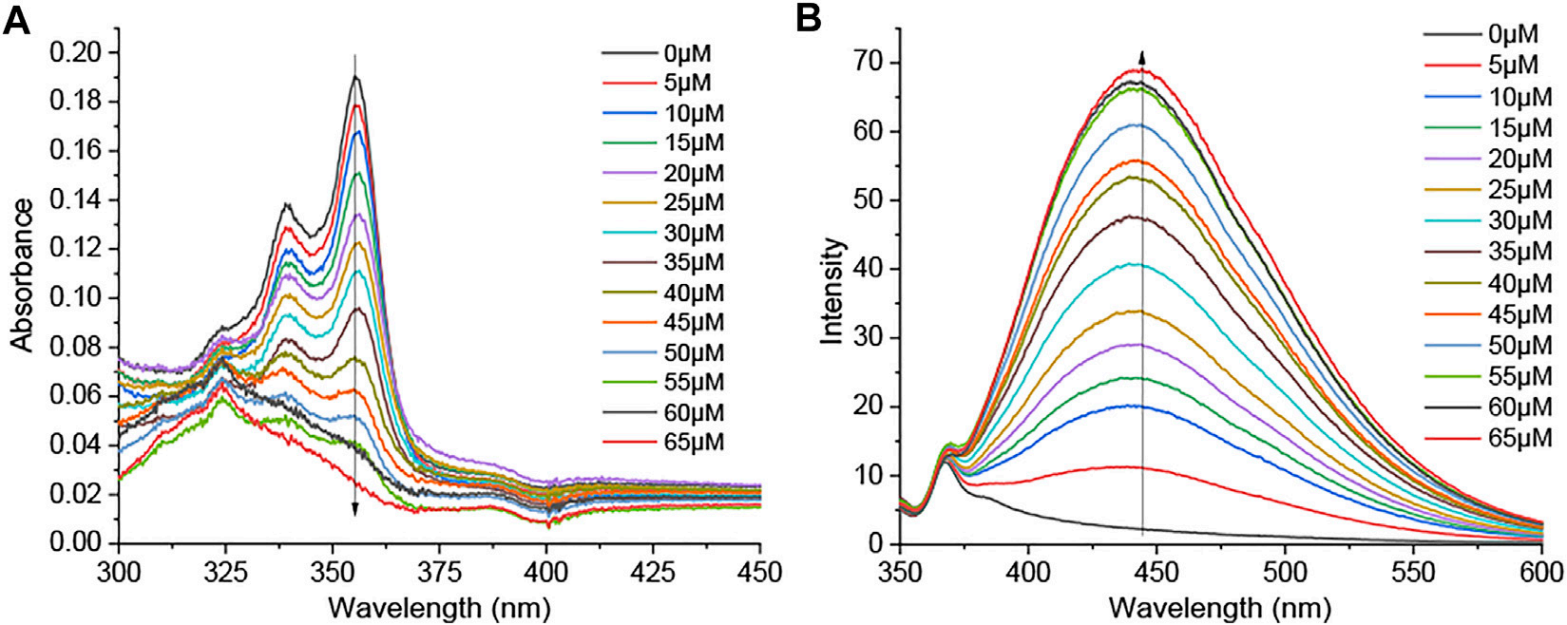
**(A)** UV–vis absorption and **(B)** fluorescence emission spectra (*λ*_ex_ = 325 nm; slit 5/5) of **DSM** (5 µM) upon treatment with ClO^−^ (0–65 µM) in PBS with a pH value of 7.4 (0.05% DMF).

Recognition of ROS/RNS by **DSM** was observed through UV–vis absorption and fluorescence emission spectroscopy in PBS buffer with a pH value of 7.4 (0.5% DMF). The absorbance band (300–375 nm) of **DSM** (5 µM) is decreased, and its blue fluorescence emission is significantly enhanced after 30 min of incubation in ClO^−^ (50 µM). In sharp contrast, the UV–vis absorption spectra of **DSM** are slightly changed, and its fluorescence emission is quenched upon exposure to other types of ROS/RNS, even at higher concentrations (Figure 6A; Supplementary Figure S51). On the other hand, when **DSD** (5 µM) was treated with ClO^−^ (0–160 µM), its fluorescence emission was also inhibited (Supplementary Figure S50A). **DSD** cannot react to ClO^−^ owing to steric hindrance between the two dibromobenzyl groups. Thus, **DSM** showed a highly selective response to ClO^−^ among various ROS/RNS, relative to other **R**_**1**_ **SR**_**2**_ (Figure 6A). Upon the gradual addition of ClO^−^ (0–65 µM) to **DSM** (5 µM) in PBS buffer with a pH value of 7.4 (0.5% DMF), the absorption band at 300–375 nm decreased, and an absorption peak appeared at about 325 nm (Figure 7A). Furthermore, the fluorescence intensity of **DSM** (5 µM) at ∼445 nm was significantly increased in the presence of ClO^−^ (50–65 µM) (Figure 7A), which is attributed to the appearance of **DIM** *via* desulfurization (Figure 6B). Thiourea, a basic form of N_2_C=S, occurs in two tautomeric isomers, a thione form and a thiol form. The thiol form of **DSM** can react with reactive ClO^−^, leading to the breakage of the C–S bond and the formation of **DIM** (Figure 6B). The reaction of this fused imidazolium salt was referred to in several previous reports (Xu et al., 2015; Xu et al., 2016). The conversion of **DSM** to imidazolium salt was examined *via* the reaction of **DSM** and ClO^−^ under similar conditions. The obtained main product (**DSM**′) was confirmed with **DIM** by ^1^H NMR, ^13^C NMR, and ESI-HRMS (Supplementary Figures S35, S36B). Furthermore, **DSM** was highly sensitive to ClO^−^, with a detection limit (LOD) of 0.13 µM (Supplementary Figure S50B), which is lower than that of many ClO^−^ probes in previous reports (Xiao et al., 2015; Shen et al., 2017; Wang et al., 2018; Lee S. C. et al., 2020; Nguyen et al., 2020).

**FIGURE 8:**
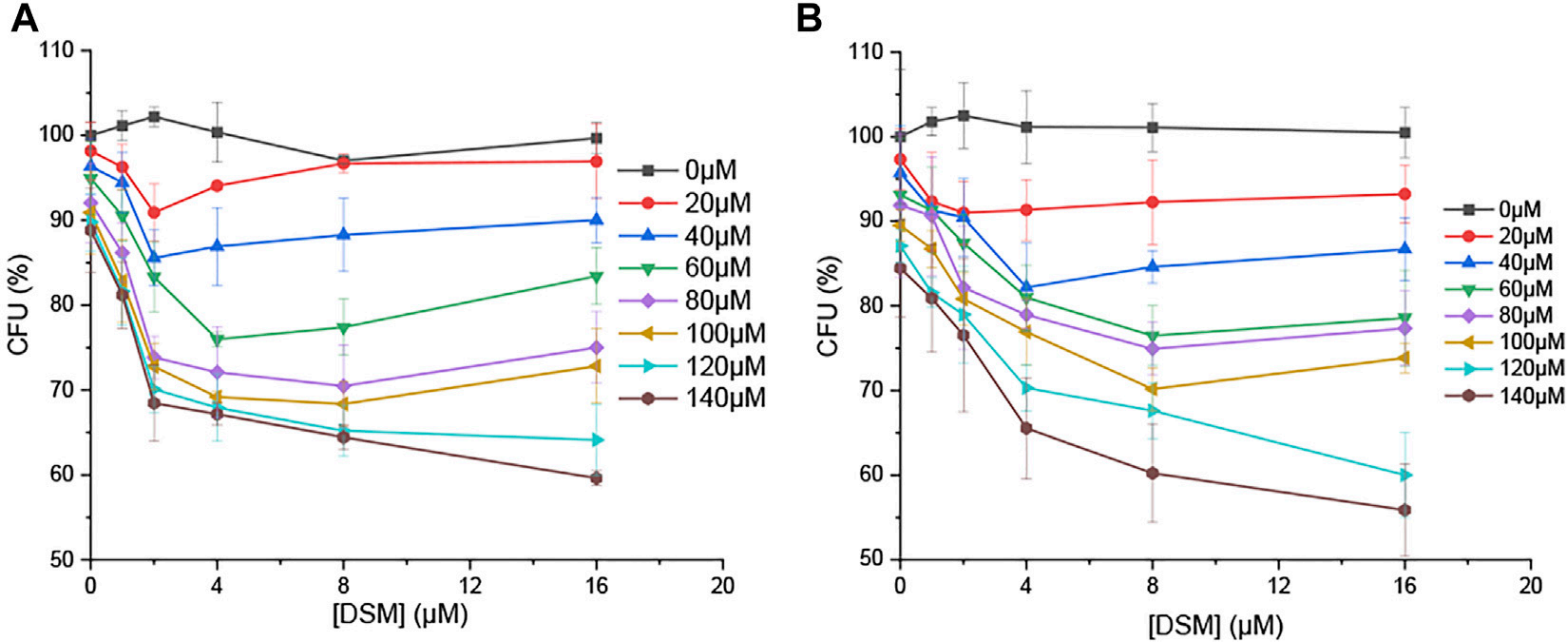
CFU percentage (%) of bacteria in the presence of **DSM** (0, 1, 2, 4, 8, and 16 µM) and ClO^−^ (0, 20, 40, 60, 80, 100, 120, and 140 µM) toward **(A)** ESBL-EC and **(B)** MRSA.

Owing to the high fluorescence emission quantum yield and antibacterial activity of **DIM**, **DSM** can act as a potential OFF-ON fluorescent and antibacterial probe in the presence of ClO^−^. Consequently, it was further studied in an antibacterial test. ESBL-EC and MRSA were treated with **DSM** (0–16 µM) and/or ClO^−^ (0–140 µM). The CFU percentage was measured after 18 h of incubation. The growth of bacteria was slightly inhibited in the presence of either ClO^−^ (0–140 µM) or **DSM** (0–16 µM) (Figure 8). In contrast, their CFU percentages decreased under simultaneous treatment with ClO^−^ and **DSM**. At ClO^−^ concentrations of 20 and 80 μM, the lowest CFU rates were achieved at **DSM** concentrations of 2 and 8 μM, respectively. At a higher concentration of ClO^−^ (140 µM), the lowest CFU rates were observed to be 59.6 and 55.9% at 16 µM of **DSM** toward ESBL-EC and MRSA bacteria, respectively. This demonstrates that **DSM** is converted to **DIM** upon ClO^−^ treatment and subsequently inhibits bacterial growth. Therefore, **DSM** can be applied as a potential ClO^−^-activated fluorophore for enhanced fluorescence emission and antibacterial activity. This finding is unprecedented with regard to previous reports of ClO^−^ fluorescent probes (Jiao et al., 2018; Pham et al., 2021b; Kwon et al., 2021).

## Conclusion

A series of imidazolium salts (**R**_**1**_ **IR**_**2**_) and imidazoline-2-thiones (**R**_**1**_ **SR**_**2**_) (R_1_, R_2_ = methyl, dibromobenzyl, and carbazole groups) were synthesized and characterized by ^1^HNMR, ^13^CNMR, mass spectra, X-ray crystal structures, and DFT calculation–based molecular orbital analysis. The excitation wavelengths of the molecules were theoretically examined *via* TD-DFT with various functions and basis sets. Imidazolium salts (**R**_**1**_ **IR**_**2**_) without a carbazole moiety showed high fluorescence emission, whereas imidazoline-2-thiones (**R**_**1**_ **SR**_**2**_) exhibited weak emission. The antibacterial activities of these compounds against *E. coli*, *S. aureus*, ESBL-EC, EC-GFP, and MRSA were studied. Among these structures, **DSM**/**DIM** and **DID**/**DSD** showed high antibacterial activity ratios that would be useful for the design of OFF-ON antibacterial probes. However, **DSD** rarely reacted with ClO^−^ due to steric hindrance, whereas **DIM** showed a high fluorescence emission quantum yield (Փ_F_ = 62.9%) that would be useful for bacterial imaging, and **DSM** exhibited a highly selective ClO^−^ response with an LOD of 0.13 µM. Finally, the **DSM** probe was converted to **DIM** upon ClO^−^ treatment and inhibited bacterial growth.

## Data Availability

The original contributions presented in the study are included in the article/Supplementary Material; further inquiries can be directed to the corresponding authors.
