# Supplementary material for: Hypochlorite-Activated Fluorescence Emission and Antibacterial Activities of Imidazole Derivatives for Biological Applications
Source: Front Chem. 2021 Jul 12;9:713078. doi: 10.3389/fchem.2021.713078 (PMC8311462; doi:10.3389/fchem.2021.713078)
Supplement: Supplementary file 1 [file DataSheet1.docx]

Supplementary Material

Contents

[1. Experimental data 2](#_Toc67318780)

[1.1. General consideration 2](#_Toc67318781)

[1.2. Synthesis process 2](#_Toc67318782)

[1.3. Fluorescence quantum yield measurements 6](#_Toc67318783)

[1.4. Generation of ROS/RNS 6](#_Toc67318784)

[1.5. LOD calculation 6](#_Toc67318785)

[1.6. Reaction of DSM with ClO^-^: 6](#_Toc67318786)

[1.7. Computational details 7](#_Toc67318787)

[1.8. Bacterial experiments 7](#_Toc67318788)

[2. Results 8](#_Toc67318789)

[2.1. NMR and Mass spectra 8](#_Toc67318790)

[2.2. Photophysical results 17](#_Toc67318791)

[2.3. Computational calculation results 22](#_Toc67318792)

[2.4. Bacterial experiments results 28](#_Toc67318793)

[3. Reference 31](#_Toc67318794)

1. **Experimental data**
   1. **General consideration**

All reagents and organic solvents used in the synthesis were obtained from Aldrich, TCI (South Korea) and used without further purification. Flash chromatography was carried out on silica gel (230-400 mesh) followed by determination of ^1^H and ^13^C NMR spectra using a Bruker Avance 400 MHz spectrometer. Mass spectra were obtained using a maXis-HD (Bruker). UV absorption spectroscopy measurements were carried out on V-730 UV-Visible Spectrophotometer (Jasco) at room temperature. Fluorescence emission spectra were obtained using an F-7000 Fluorescence Spectrophotometer (Hitachi High-Tech).

- 1. **Synthesis process**

Scheme S1. Synthesis process of R_1_IR_2_.

- - 1. Synthesis **DBr**: 2,4-Dibromotoluene (3.0 g, 2 mmol) was added in 50 mL CH_3_CN including NBS (2.3 g – 12.9 mmol). The mixture was stirred and irradiated under white lamp 25W during 30 min. The reaction solution was evaporated and purified by column chromatography on silica gel using n-hexane as eluent to obtain yellow oil as pure product (yield ~ 95 %). ^1^H NMR (400 MHz, Chloroform-*d*) δ 7.72 (d, *J* = 2.0 Hz, 1H), 7.41 (dd, *J* = 8.2, 2.0 Hz, 1H), 7.30 (d, *J* = 8.2 Hz, 1H), 4.52 (s, 2H). ^13^C NMR (101 MHz, CHLOROFORM-*D*) δ 136.61, 136.16, 132.67, 131.65, 125.52, 123.46, 32.82.

Scheme S2. Synthesis of **DBr** and **CBr**.

- - 1. Synthesis **CBr:** A mixture of carbazole (5.0 g), benzene (15 mL), 1,4 dichlorobutane (10 eq.), benzyltriethylammonium chloride (phase transfer catalyst) and aqueous sodium hydroxide (15 mL, 50%) was stirred overnight. The obtained solution was acidified with HCl to pH around 3, extracted with chloroform, and then dried over anhydrous Na_2_SO_4_. After evaporation, the viscous liquid was purified by column chromatography on silica gel using n-hexane-DCM (9/1) as eluent to obtain brown powder as pure product (yield ~ 65%). ^1^H NMR (400 MHz, Chloroform-*d*) δ 8.10 (dq, *J* = 7.7, 1.1 Hz, 2H), 7.46 (ddt, *J* = 8.2, 7.1, 1.3 Hz, 2H), 7.36 (s, 2H), 7.28 – 7.19 (m, 2H), 4.29 (td, *J* = 7.0, 1.3 Hz, 2H), 3.33 (t, *J* = 6.5 Hz, 2H), 2.08 – 1.96 (m, 2H), 1.93 – 1.81 (m, 2H); ^13^C NMR (101 MHz, Chloroform-*d*) δ 140.43, 125.89, 123.04, 120.59, 119.11, 108.70, 42.26, 33.32, 30.37, 27.78; ESI HRMS m/z = 302.0534 [M+H]^+^, calc. for C_16_H_16_BrN = 301.05.
    2. Synthesis of **I**: A mixture of 2,4-diaminonaphthalene (2.0 g) and formic acid (20 mL) was refluxed at 100 °C for 24 h. The obtained solution was added NaOH solution (50%) to pH 9, extracted with chloroform, and then dried over anhydrous Na_2_SO_4_. After evaporation, the crude product was purified by column chromatography on silica gel using n-hexane-Methanol (95/5) as eluent to obtain white powder as pure product (yield ~ 75%). ^1^H NMR (400 MHz, Acetone-*d*_6_) δ 8.43 (s, 1H), 8.15 (s, 2H), 7.99 (dt, *J* = 6.0, 3.0 Hz, 2H), 7.42 – 7.34 (m, 2H). ^13^C NMR (101 MHz, DMSO-*D*_6_) δ 147.08, 130.33, 128.42, 123.98. ESI HRMS m/z = 169.0761 [M+H]^+^, calc. for C_11_H_8_N_2_ = 168.07.
    3. Synthesis of **R_1_I**: NaH (200 mg) was added to the mixture of **I** (1.0 g, 5.95 mmol) and tetrahydrofuran (20 mL) under ice bath, and the mixture was stirred for 30 min. Then, **R_­1_Br** or **R_2_Br** (6.0 mmol) was added, and ice bath was removed. The mixture was refluxed under N_2_ overnight. After solvent evaporation, water was added and washed with MC. Organic phase was collected, dried over anhydrous Na_2_SO_4_, then evaporation. The crude product was purified by column chromatography on silica gel using n-hexane-Ethyl acetate (9/1) as eluent to obtain brown powder as pure product (yield ~ 50%).
       1. **BI**: ^1^H NMR (400 MHz, Chloroform-*d*) δ 8.32 (s, 1H), 8.12 (s, 1H), 8.04 – 7.96 (m, 1H), 7.91 – 7.83 (m, 1H), 7.70 – 7.59 (m, 2H), 7.45 – 7.34 (m, 2H), 7.23 – 7.12 (m, 2H), 6.87 – 6.78 (m, 1H), 5.50 (s, 2H); ^13^C NMR (101 MHz, CHLOROFORM-*D*) δ 147.43, 143.93, 134.68, 134.46, 133.37, 130.75, 130.33, 129.95, 128.70, 128.63, 128.15, 127.59, 124.79, 123.82, 122.98, 117.83, 105.93, 49.10; ESI HRMS m/z = 337.0339 [M+H]^+^, calc. for C_18_H_13_BrN_2_ = 336.03.
       2. **CI**: ^1^H NMR (400 MHz, Chloroform-*d*) δ 8.25 (s, 1H), 8.09 (dt, *J* = 7.7, 1.0 Hz, 2H), 8.01 – 7.95 (m, 1H), 7.91 – 7.79 (m, 2H), 7.60 (s, 1H), 7.46 – 7.36 (m, 4H), 7.33 (dt, *J* = 8.2, 0.9 Hz, 2H), 7.23 (ddd, *J* = 7.9, 7.1, 1.0 Hz, 3H), 4.33 (d, *J* = 5.8 Hz, 2H), 4.05 (d, *J* = 5.8 Hz, 2H), 3.47 (s, 2H); ^13^C NMR (101 MHz, CHLOROFORM-*D*) δ 140.31, 130.55, 130.16, 128.67, 127.53, 125.94, 124.68, 123.69, 123.03, 120.64, 119.25, 117.62, 108.58, 105.41, 50.92, 44.95, 42.46, 27.48, 26.37; ESI HRMS m/z = 390.1967 [M+H]^+^, calc. for C_27_H_23_N_3_ = 389.19.
       3. **DI:** ^1^H NMR (400 MHz, Chloroform-*d*) δ 8.32 (s, 1H), 8.12 (s, 1H), 8.04 – 7.96 (m, 1H), 7.91 – 7.84 (m, 1H), 7.81 (d, *J* = 2.0 Hz, 1H), 7.63 (s, 1H), 7.46 – 7.35 (m, 2H), 7.30 (dd, *J* = 8.3, 2.0 Hz, 1H), 6.69 – 6.62 (m, 1H), 5.44 (s, 2H). ^13^C NMR (101 MHz, CHLOROFORM-*D*) δ 147.25, 143.81, 135.73, 134.22, 133.82, 131.33, 130.79, 130.39, 129.58, 128.72, 127.56, 124.95, 123.95, 123.38, 122.80, 118.00, 105.83, 48.66. ESI HRMS m/z = 414.9443 [M+H]^+^, calc. for C_18_H_12_Br_2_N_2_ = 413.94.
    4. Synthesis of **R_1_IR_2_**: A mixture of **R_1_I** (1.0 mmol) and **R_2_Br** or **CH_3_I** (1.2 mmol) in CH_3_CN were refluxed overnight. After cooled to room temperature, solvents were removed, then residue was dissolved in methanol (1 mL). Hexane (15-25 mL) was added to the solution, vibrate in Ultrasonic Cleaner and the precipitate was collected by filter to obtain the product as white solid (yield ~ 90%).
       1. **BIB**: ^1^H NMR (400 MHz, DMSO-d6) δ 10.03 (s, 1H), 8.61 (s, 2H), 8.22 – 8.13 (m, 2H), 7.79 (dd, J = 7.8, 1.4 Hz, 2H), 7.70 – 7.62 (m, 2H), 7.47 – 7.35 (m, 6H), 5.95 (s, 4H). ^13^C NMR (101 MHz, DMSO-D6) δ 148.43, 133.86, 133.19, 131.64, 131.46, 130.81, 130.71, 129.01, 128.84, 127.52, 123.43, 112.08, 51.24. ESI HRMS m/z = 504.9909 [M]^+^, calc. for C_25_H_19_Br_2_N_2_ = 504.99.
       2. **BIM**: ^1^H NMR (400 MHz, DMSO-d6) δ 9.91 (s, 1H), 8.68 (s, 1H), 8.55 (s, 1H), 8.28 – 8.21 (m, 1H), 8.21 – 8.14 (m, 1H), 7.80 (dd, J = 7.8, 1.6 Hz, 1H), 7.73 – 7.62 (m, 2H), 7.47 – 7.34 (m, 3H), 5.90 (s, 2H), 4.20 (s, 3H). ^13^C NMR (101 MHz, DMSO-D6) δ 147.92, 133.81, 133.28, 131.66, 131.55, 131.50, 131.44, 130.80, 130.68, 128.97, 128.84, 128.81, 127.31, 123.48, 111.87, 111.47, 50.87, 34.13. ESI HRMS m/z = 504.9909 [M]^+^, calc. for C_25_H_19_Br_2_N_2_ = 504.99. ESI HRMS m/z = 351.0497 [M]^+^, calc. for C_19_H_16_BrN_2_ = 351.05.
       3. **CIB**: ^1^H NMR (400 MHz, DMSO-*d*_6_) δ 9.93 (s, 1H), 8.68 (s, 1H), 8.52 (s, 1H), 8.23 – 8.09 (m, 4H), 7.74 (ddd, *J* = 7.1, 2.8, 1.6 Hz, 1H), 7.72 – 7.64 (m, 2H), 7.61 (dt, *J* = 8.2, 0.9 Hz, 2H), 7.41 (ddd, *J* = 8.3, 7.1, 1.2 Hz, 2H), 7.39 – 7.32 (m, 3H), 7.18 (ddd, *J* = 7.9, 7.2, 0.9 Hz, 2H), 5.84 (s, 2H), 4.64 (t, *J* = 7.0 Hz, 2H), 4.48 (t, *J* = 6.9 Hz, 2H), 2.07 (q, *J* = 7.4 Hz, 2H), 1.91 (dq, *J* = 14.5, 7.0 Hz, 2H). ^13^C NMR (101 MHz, DMSO-D6) δ 147.56, 140.50, 133.90, 133.20, 131.64, 131.58, 131.52, 131.02, 130.84, 130.75, 129.03, 128.90, 127.45, 126.27, 123.54, 122.67, 120.91, 119.36, 112.03, 111.75, 109.86, 51.13, 47.44, 42.26, 31.55, 26.53, 26.13, 22.66, 14.56. ESI HRMS m/z = 558.1539 [M]^+^, calc. for C_34_H_29_BrN_3_ = 558.15.
       4. **CIC**: ^1^H NMR (600 MHz, DMSO-*d*_6_) δ 9.82 (s, 1H), 8.55 (d, *J* = 2.3 Hz, 2H), 8.14 (dt, *J* = 5.7, 2.7 Hz, 2H), 8.10 (d, *J* = 7.7 Hz, 4H), 7.69 (dt, *J* = 5.8, 2.6 Hz, 2H), 7.56 (d, *J* = 8.2 Hz, 4H), 7.39 (t, *J* = 7.8 Hz, 4H), 7.16 (t, *J* = 7.3 Hz, 4H), 4.51 (t, *J* = 7.2 Hz, 4H), 4.43 (t, *J* = 7.2 Hz, 4H), 2.01 (p, *J* = 7.6 Hz, 4H), 1.87 (p, *J* = 7.3 Hz, 4H). ^13^C NMR (101 MHz, DMSO-*D*_6_) δ 146.60, 140.40, 131.41, 130.67, 128.78, 127.17, 126.18, 122.58, 120.84, 119.29, 111.50, 109.74, 47.14, 42.16, 26.34, 26.00. ESI HRMS m/z = 611.3169 [M]^+^, calc. for C_43_H_39_N_3_ = 611.32.
       5. **CID**: ^1^H NMR (600 MHz, DMSO-*d*_6_) δ 9.87 (s, 1H), 8.68 (s, 1H), 8.53 (s, 1H), 8.17 (ddd, *J* = 8.6, 7.1, 1.7 Hz, 2H), 8.12 (dt, *J* = 7.7, 0.9 Hz, 2H), 8.01 (d, *J* = 2.0 Hz, 1H), 7.73 – 7.65 (m, 2H), 7.64 – 7.57 (m, 3H), 7.41 (ddd, *J* = 8.2, 7.1, 1.2 Hz, 2H), 7.30 (d, *J* = 8.4 Hz, 1H), 7.18 (ddd, *J* = 7.9, 7.1, 0.9 Hz, 2H), 5.79 (s, 2H), 4.62 (t, *J* = 7.1 Hz, 2H), 4.47 (t, *J* = 7.1 Hz, 2H), 2.06 (p, *J* = 7.1 Hz, 2H), 1.89 (p, *J* = 7.3 Hz, 2H). ^13^C NMR (101 MHz, DMSO-*D*_6_) δ 146.66, 139.65, 134.93, 131.91, 131.77, 131.12, 130.81, 129.89, 128.06, 126.63, 125.43, 123.77, 122.63, 121.83, 120.09, 118.53, 111.19, 110.90, 109.02, 49.80, 46.63, 41.44, 25.70, 25.31. ESI HRMS m/z = 636.0654 [M]^+^, calc. for C_34_H_28_Br_2_N_3_ = 636.06.
       6. **CIM**: ^1^H NMR (400 MHz, DMSO-*d*_6_) δ 9.81 (s, 1H), 8.59 (d, *J* = 2.6 Hz, 2H), 8.25 – 8.19 (m, 1H), 8.19 – 8.15 (m, 1H), 8.15 – 8.08 (m, 2H), 7.70 (dt, *J* = 6.0, 3.4 Hz, 2H), 7.62 (d, *J* = 8.2 Hz, 2H), 7.43 (ddd, *J* = 8.3, 7.1, 1.2 Hz, 2H), 7.23 – 7.14 (m, 2H), 4.58 (t, *J* = 7.0 Hz, 2H), 4.49 (t, *J* = 6.8 Hz, 2H), 4.09 (s, 3H), 3.39 (s, 5H), 2.06 (dd, *J* = 17.1, 8.6 Hz, 2H), 1.94 (t, *J* = 7.7 Hz, 2H). ^13^C NMR (101 MHz, DMSO-*D*_6_) δ 147.24, 140.44, 131.57, 131.40, 130.57, 128.81, 128.77, 127.15, 127.12, 126.20, 122.58, 120.83, 119.29, 111.44, 111.30, 109.80, 47.07, 42.24, 33.88, 26.56, 26.07. ESI HRMS m/z = 404.2121 [M]^+^, calc. for C_28_H_26_N_3_ = 404.21.
       7. **DIB**: ^1^H NMR (400 MHz, DMSO-*d*_6_) δ 10.01 (s, 1H), 8.62 (d, *J* = 6.5 Hz, 2H), 8.18 (dt, *J* = 7.4, 3.1 Hz, 2H), 8.09 (d, *J* = 2.0 Hz, 1H), 7.80 (dd, *J* = 7.7, 1.5 Hz, 1H), 7.72 – 7.63 (m, 3H), 7.48 – 7.30 (m, 4H), 5.93 (d, *J* = 10.0 Hz, 4H). ^13^C NMR (101 MHz, DMSO-*D*_6_) δ 148.44, 135.74, 133.86, 133.17, 132.72, 132.34, 131.95, 131.66, 131.44, 130.79, 130.76, 130.61, 129.00, 128.85, 127.54, 124.51, 123.39, 112.08, 51.24, 50.79. ESI HRMS m/z = 582.9015 [M]^+^, calc. for C_25_H_18_Br_3_N_2_ = 582.90.
       8. **DID**: ^1^H NMR (400 MHz, DMSO-*d*_6_) δ 9.97 (s, 1H), 8.61 (s, 2H), 8.22 – 8.14 (m, 2H), 8.08 (d, *J* = 2.0 Hz, 2H), 7.72 – 7.62 (m, 4H), 7.31 (d, *J* = 8.3 Hz, 2H), 5.90 (s, 4H). ^13^C NMR (101 MHz, DMSO-*D*_6_) δ 135.74, 132.73, 132.25, 131.95, 131.69, 130.76, 128.89, 127.59, 124.47, 123.36, 112.09, 50.80. ESI HRMS m/z = 660.8120 [M]^+^, calc. for C_25_H_17_Br_4_N_2_ = 660.81.
       9. **DIM**: ^1^H NMR (400 MHz, DMSO-*d*_6_) δ 9.87 (s, 1H), 8.69 (s, 1H), 8.56 (s, 1H), 8.28 – 8.22 (m, 1H), 8.22 – 8.15 (m, 1H), 8.09 (d, *J* = 2.0 Hz, 1H), 7.74 – 7.63 (m, 3H), 7.34 (d, *J* = 8.3 Hz, 1H), 5.87 (s, 2H), 4.19 (s, 3H). ^13^C NMR (101 MHz, DMSO-*D*_6_) δ 135.71, 132.41, 131.91, 131.65, 128.87, 127.35, 124.57, 123.38, 111.88, 111.49, 50.42, 34.10. ESI HRMS m/z = 428.9596 [M]^+^, calc. for C_19_H_15_Br_2_N_2_ = 428.96.
  1. **Fluorescence quantum yield measurements**

The photoluminescence quantum yield (Փ_F_) was determined according to following equation:

$$Փ_{s}=Փ_{ref}\times\left( \frac{A_{ref}}{A_{s}} \right)\times\left( \frac{I_{s}}{I_{ref}} \right)\times\left( \frac{\eta_{s}}{\eta_{ref}} \right)^{2}$$

Where the subscripts *ref* and *s* denote reference and test respectively, Փ is the fluorescence quantum yield, A is absorbance of test solution, I is integrated fluorescence intensity, and η the refractive index of the solvent. 9,10-Diphenylanthracene (Փ_F_ = 0.90 in Cyclohexane) was used as a reference.^1^

- 1. **Generation of ROS/RNS**

H_2_O_2_ was diluted from a 28 % solution in water, and tert-butyl hydroperoxide was diluted from a 70 % solution in water. ROO· was generated from 2,2’- azobis(2-amidinopropane)dihydrochloride, NO· was generated from SNP (sodium nitroferricyanide(III)dihydrate), and ·OH was generated from the reaction of iron(II) chloride with H_2_O_2_. ONOO^−^ was prepared according to previous literature, and the concentration was determined by the absorbance at 302 nm.^2^ NaClO was obtained by dilution of 5 % of the solution in water. The above ROS or reactive nitrogen species (RNS) was incubated with the probe in a PBS (pH 7.4)/THF (5/5) mixture for 15 min.

- 1. **LOD calculation**

The limit of detection was calculated according to the literature procedure. ^3^ The linear calibration cure was assumed that the response y is linearly related to the concentration x for a limited range of concentration and expressed as y = a + bx. This model was used to determine the sensitivity (b) and the LOD value. The limit of detection (LOD) was calculated using the following equation: LOD=3×S_a_/b, where S_a_ is the standard deviation of the response and b as the slope of calibration cure.

- 1. **Reaction of DSM with ClO^-^:**

0.1 mL of NaClO solution (5%) was added slowly to the solution of **DSM** (100 mg) in CH_3_CN (25 mL) under stirring 30 min. The solvent was removed under reduced pressure, the residue was purified by silica gel column chromatography, using MC/MeOH (from 9/1) as eluent to get a white solid as the main product (**DSM’**) (yield ~ 91 %) which assigned **DIM**. ^1^H NMR (400 MHz, DMSO-*d*_6_) δ 9.90 – 9.85 (m, 1H), 8.68 (d, *J* = 1.1 Hz, 1H), 8.55 (d, *J* = 1.0 Hz, 1H), 8.27 – 8.21 (m, 1H), 8.21 – 8.14 (m, 1H), 8.08 (d, *J* = 2.0 Hz, 1H), 7.73 – 7.62 (m, 3H), 7.33 (d, *J* = 8.3 Hz, 1H), 5.86 (s, 2H), 4.21 – 4.16 (m, 3H); ^13^C NMR (101 MHz, DMSO-*D*_6_) δ 147.38, 135.14, 132.27, 131.86, 131.35, 131.10, 131.01, 130.96, 130.07, 128.31, 128.27, 126.78, 124.01, 122.83, 111.33, 110.93, 49.87, 40.13, 33.59; ESI HRMS m/z = 428.9602 [M]^+^, calc. for C_19_H_15_Br_2_N_2_ = 428.96.

- 1. **Computational details**

The DFT calculations of the molecules were performed using the Gaussian 09 program package. Geometry optimizations of the molecule were performed using the B3LYP hybrid functional with 6-31+G(2d,p) basis set.^4-5^ After optimizing structures, vibrational frequencies were computed to ensure that there are no imaginary frequencies. Optical excitation energies were calculated with various functionals using time-dependent DFT (TD-DFT) with the 6-31+G(2d,p), def-2-TZVP and cc-PVTZ basis sets in ACN solvent.^6^ The solvent was modeled by the polarizable continuum model (PCM) using the integral equation formalism variant (IEFPCM) as implemented in Gaussian 09. Our calculated excitation energies using the Cam-B3LYP functional with Def2-TZVP for **R_1_IR_2_** and TPSSTPSS functional with 6-31+G(2d,p) for **R_1_SR_2_** were nearest to the experimental data (Table S2). Natural transition-orbital (NTO) analysis was done to characterize the nature of different molecular excited states.^7^

- 1. **Bacterial experiments**

Three strains of gram-negative bacteria (*E. Coli, ESBL EC, EC GFP*) and two strains of gram-positive bacteria (*S. Aureus, MRSA*) were procured from the Korean Collection for Type Cultures (KCTC) at the Korea Research Institute of Bioscience and Biotechnology (KRIBB). Three isolated colonies were transferred to 3 mL LB broth and grown overnight at 37 °C in a shaking incubator at 250 rpm. The final test concentration of bacterial suspension was adjusted to 2×10^5^ CFU/mL in LB broth. **R_1_IR_2_** and **R_1_SR_2_** were dissolved in DMSO. The obtained solution was diluted with sterilized LB broth to give a stock solution of 256 µM in 1% DMSO (stock solution). The stock solution was diluted two-fold serially in the LB in microplates to a final volume of 100 μL in each well. The same volume (100 μL) of bacterial suspension was added in each well making concentration range of 1, 2 to 128 µM for each test compound. The microplates were then incubated at 37 °C for 18 h, and the absorbance at 600 nm was measured using a FilterMax™ F5 Multi-Mode Microplate Reader (Molecular Devices, USA). CFU rate (%) was calculated by ratio of CFU value between bacteria treated with/without reagents. To compare the antibacterial activity between couple of **R_1_IR_2_** and **R_1_SR_2_,** we calculated the concentration (µM) (CFU_50_­), at which the CFU rate equals 50 %; and the $P= \frac{{CFU}_{50(R_{1}IR_{2})}}{{CFU}_{50(R_{1}SR_{2})}}$ between a couple of imidazolium salt and imidazoline-2-thiones. The CFU_50_ of imidazoline-2-thiones_­_ are higher than 128 µM.

1. **Results**
   1. **NMR and Mass spectra**


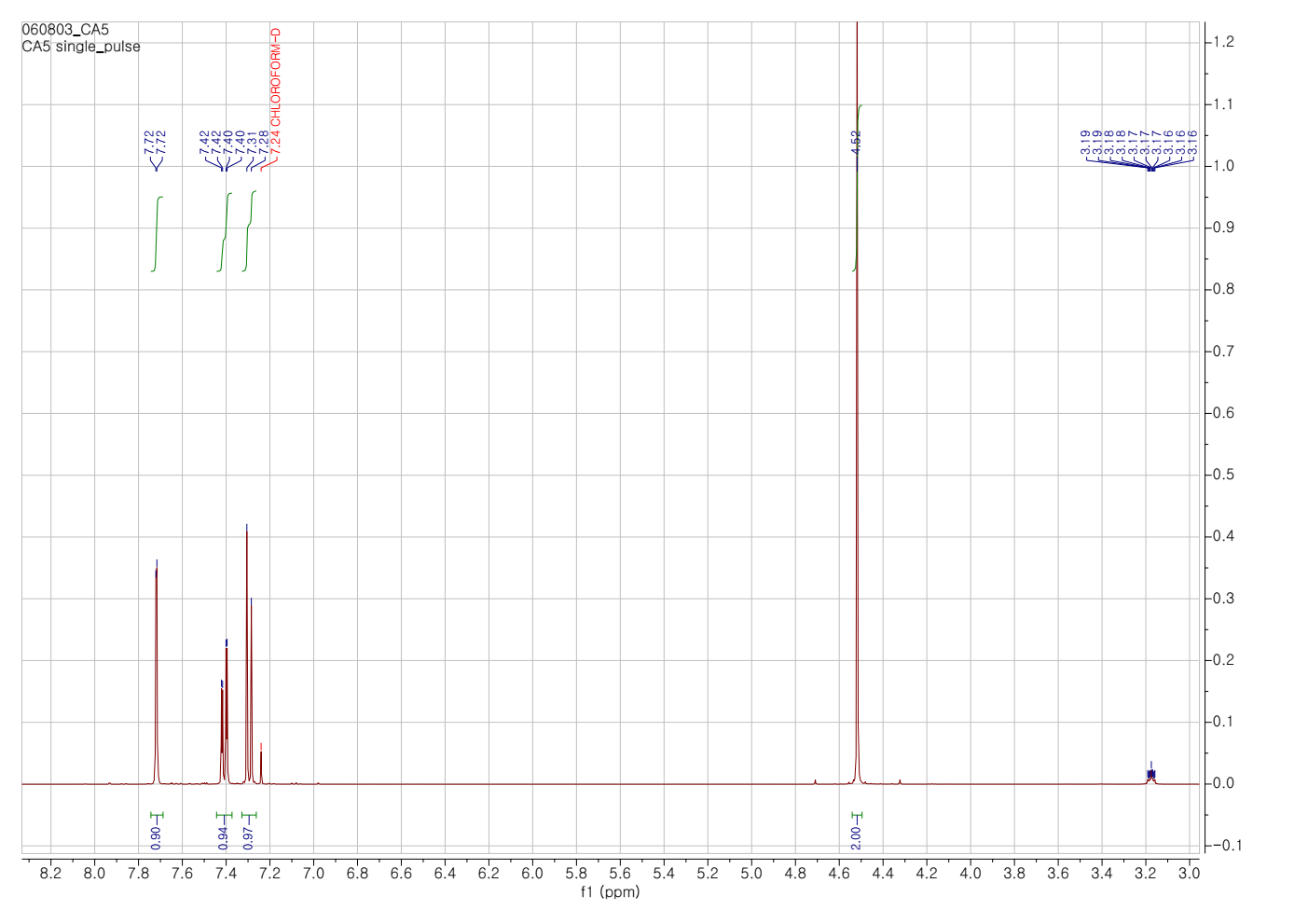

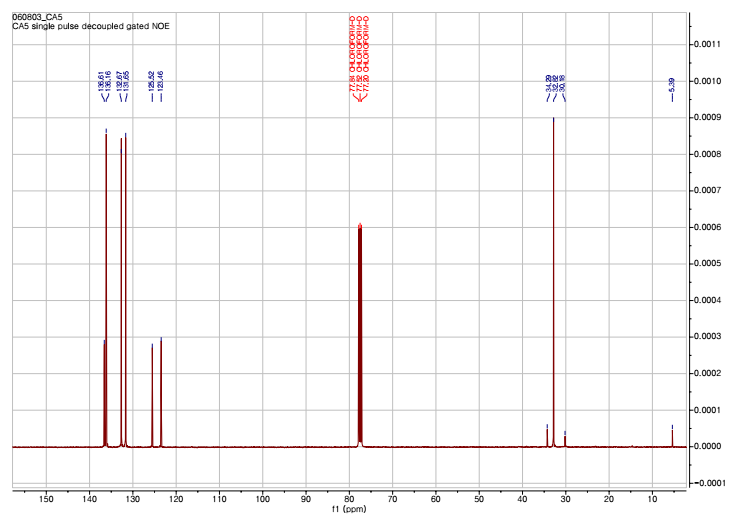


Figure S1. ^1^H-NMR and ^13^C-NMR spectra of **DBr** in CDCl_3_



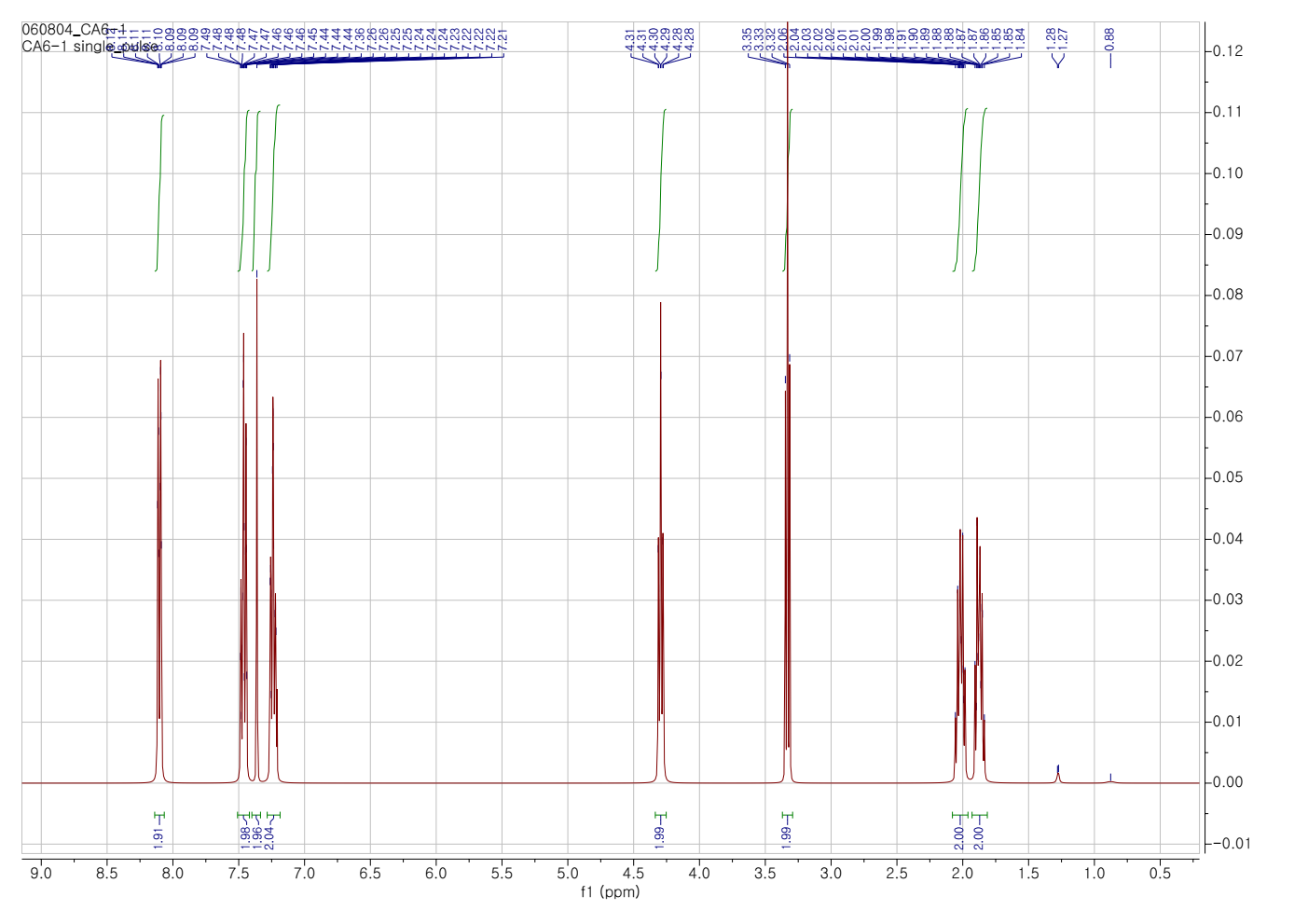

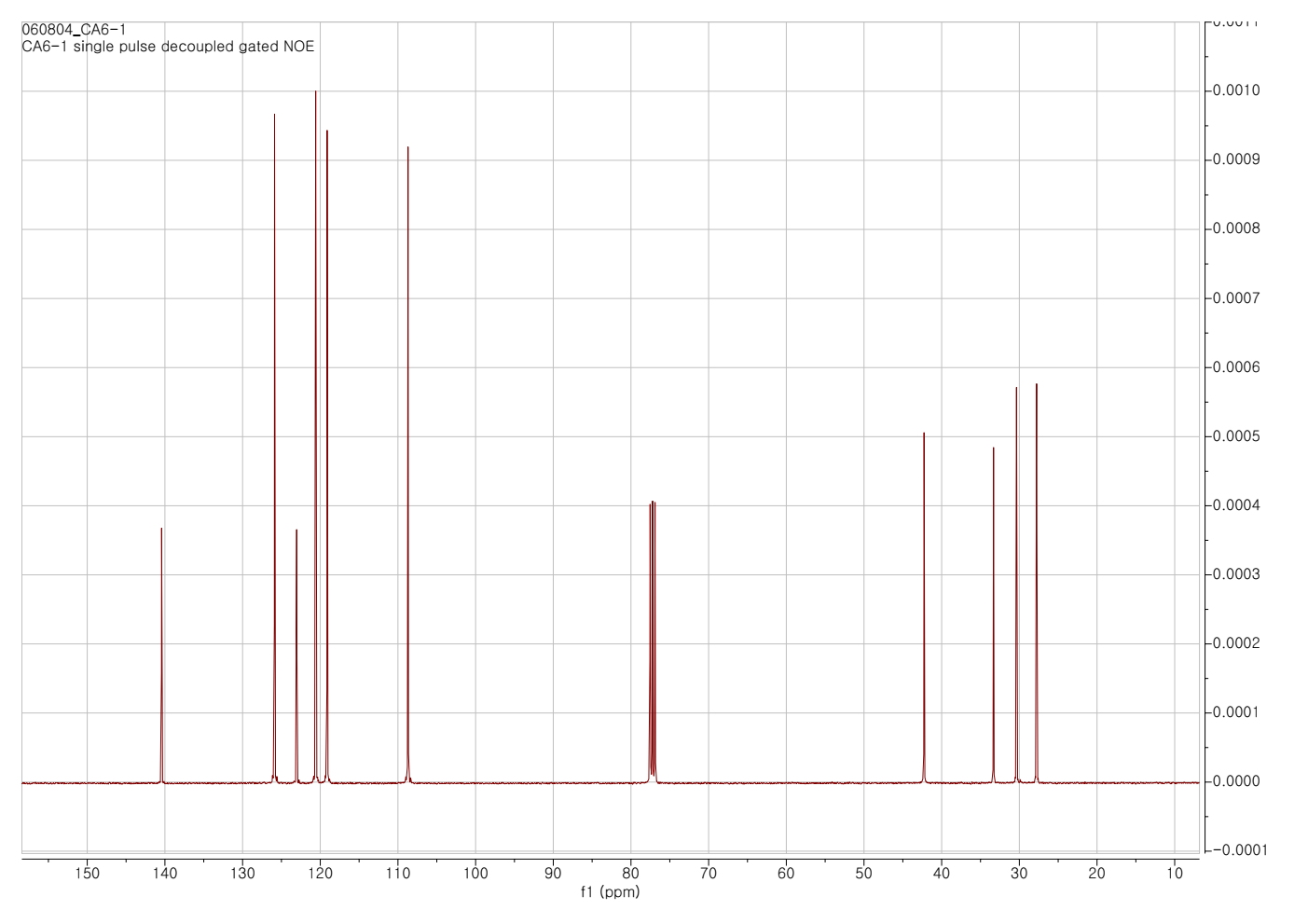


Figure S2. ^1^H-NMR and ^13^C-NMR spectra of **CBr** in CDCl_3_



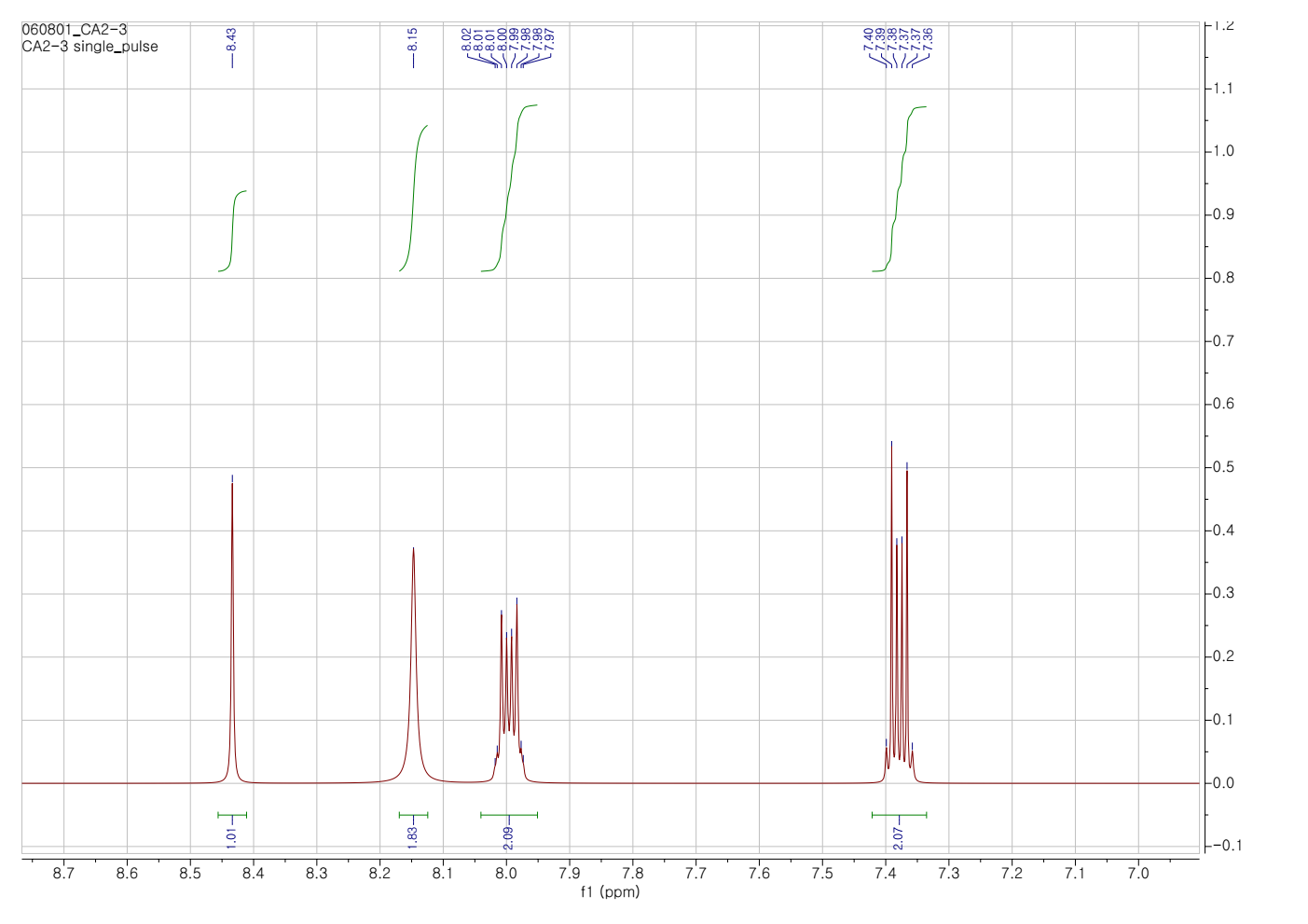

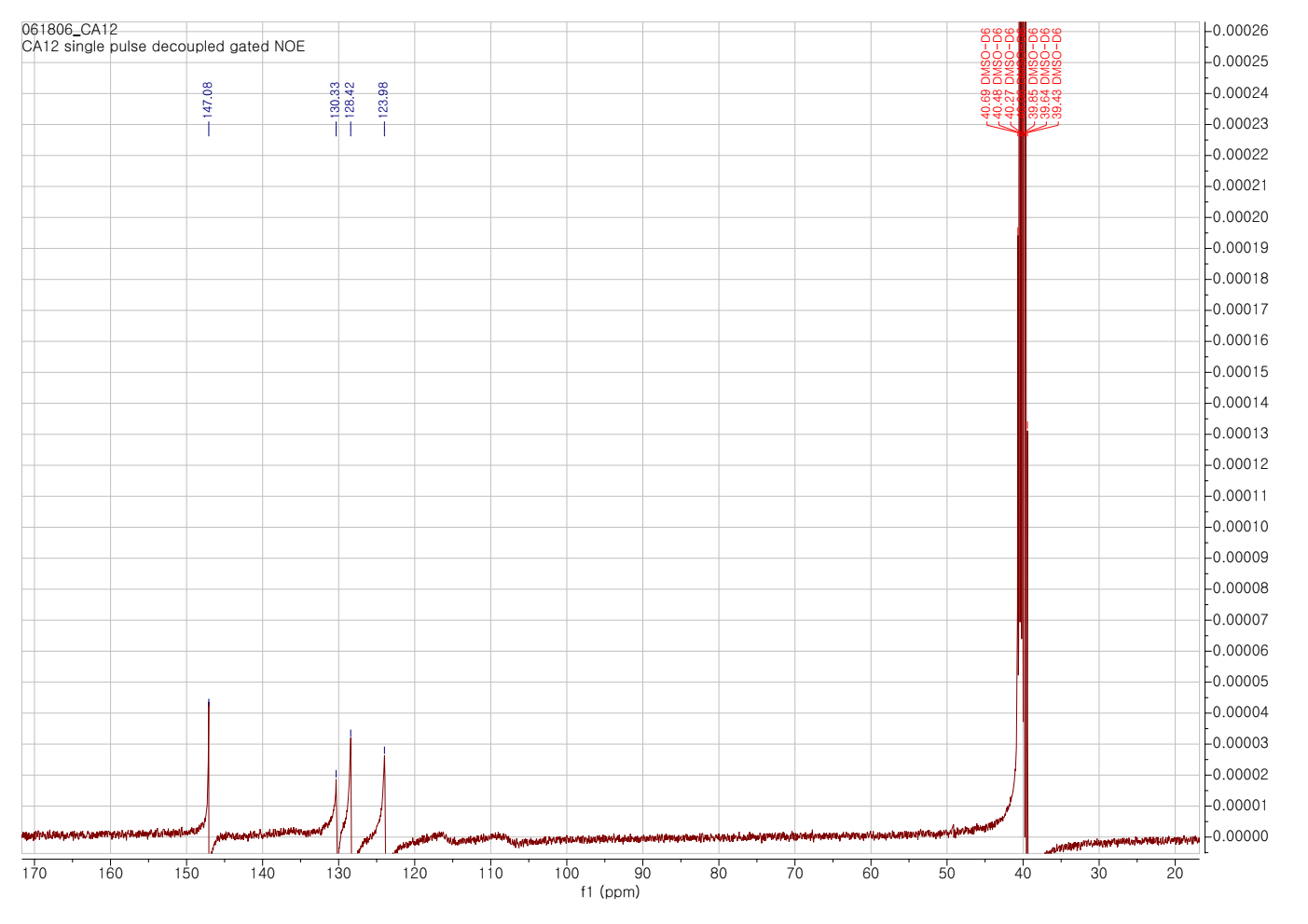


Figure S3. ^1^H-NMR and ^13^C-NMR spectra of **I** in DMSO

| _a) Mass spectra of_ **_CBr_**  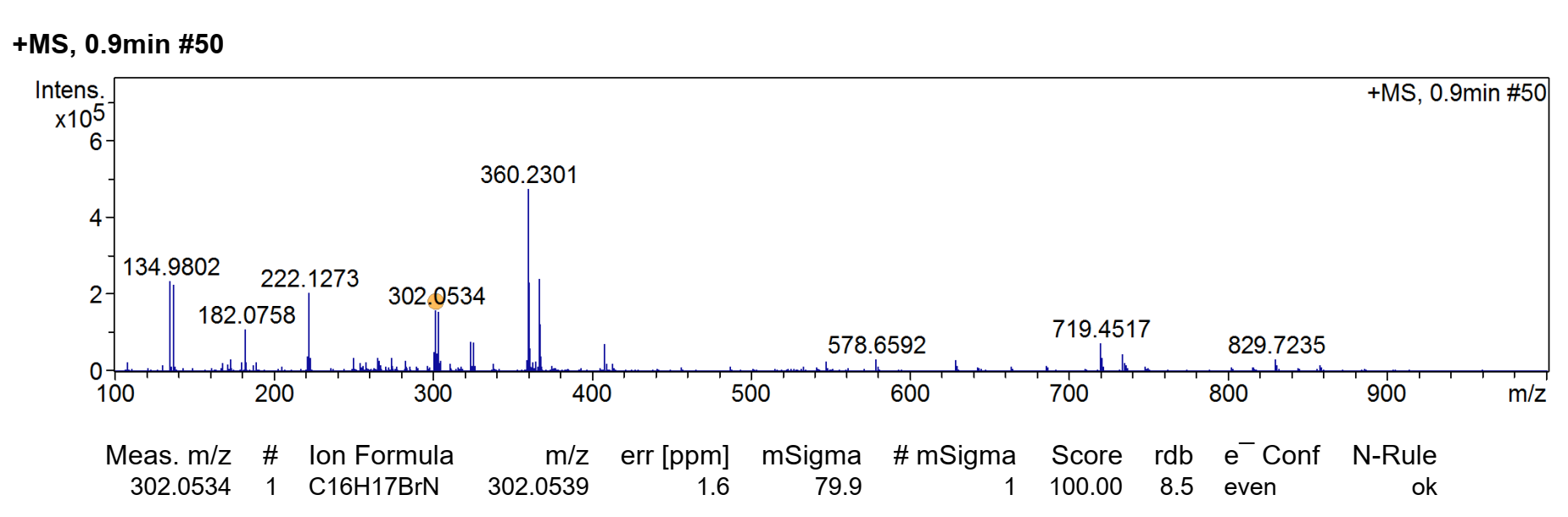 | _b) Mass spectra of_ **_I_**  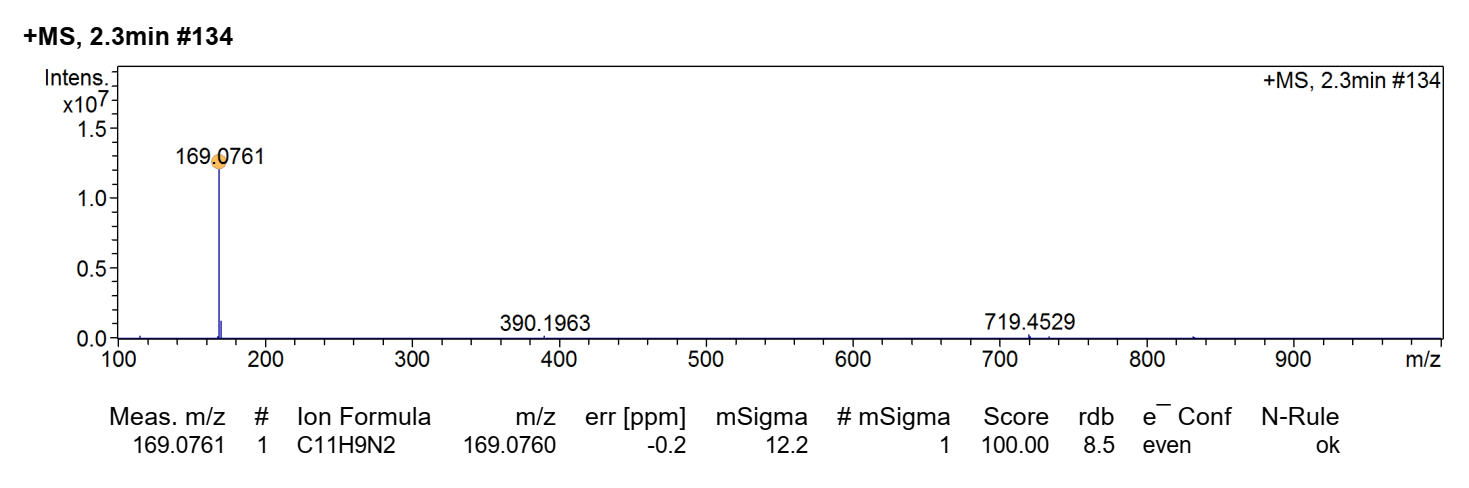 |
| --- | --- |

Figure S4. Mass spectra of **CBr** and **I**.

| a) Mass spectra of **BI**  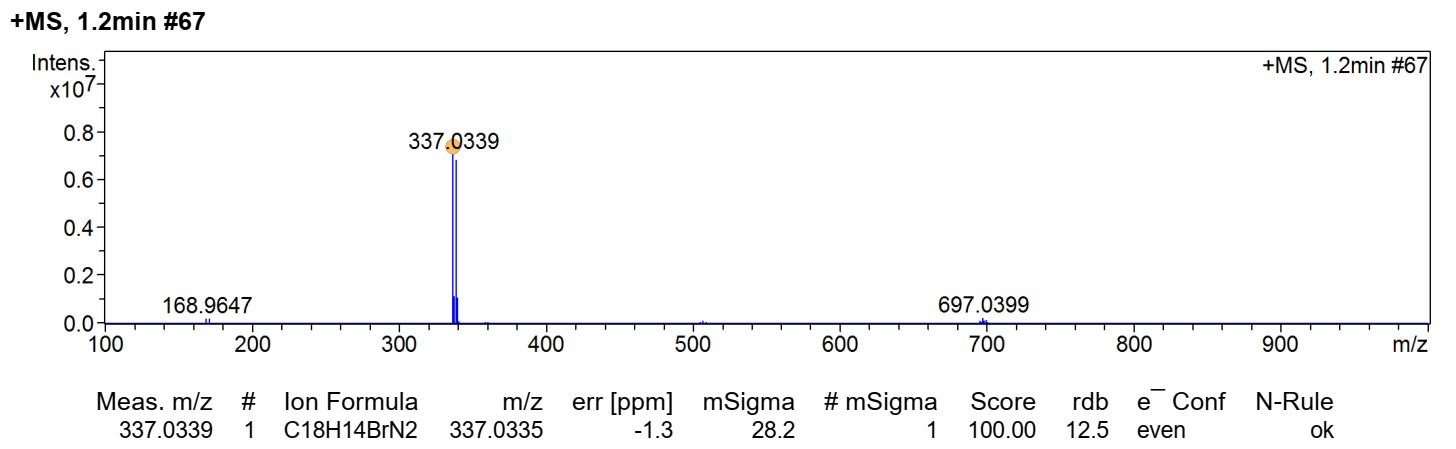 | b) Mass spectra of **CI**  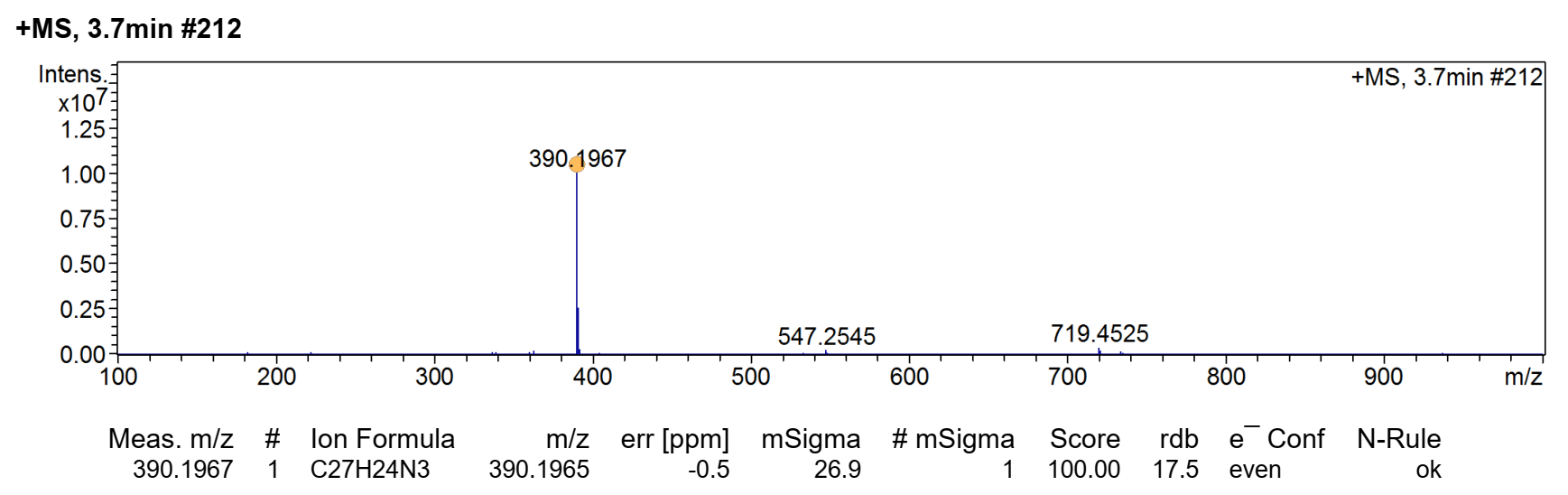 |
| --- | --- |

Figue S5. Mass spectra of (a) **BI** and (b) **CI**.


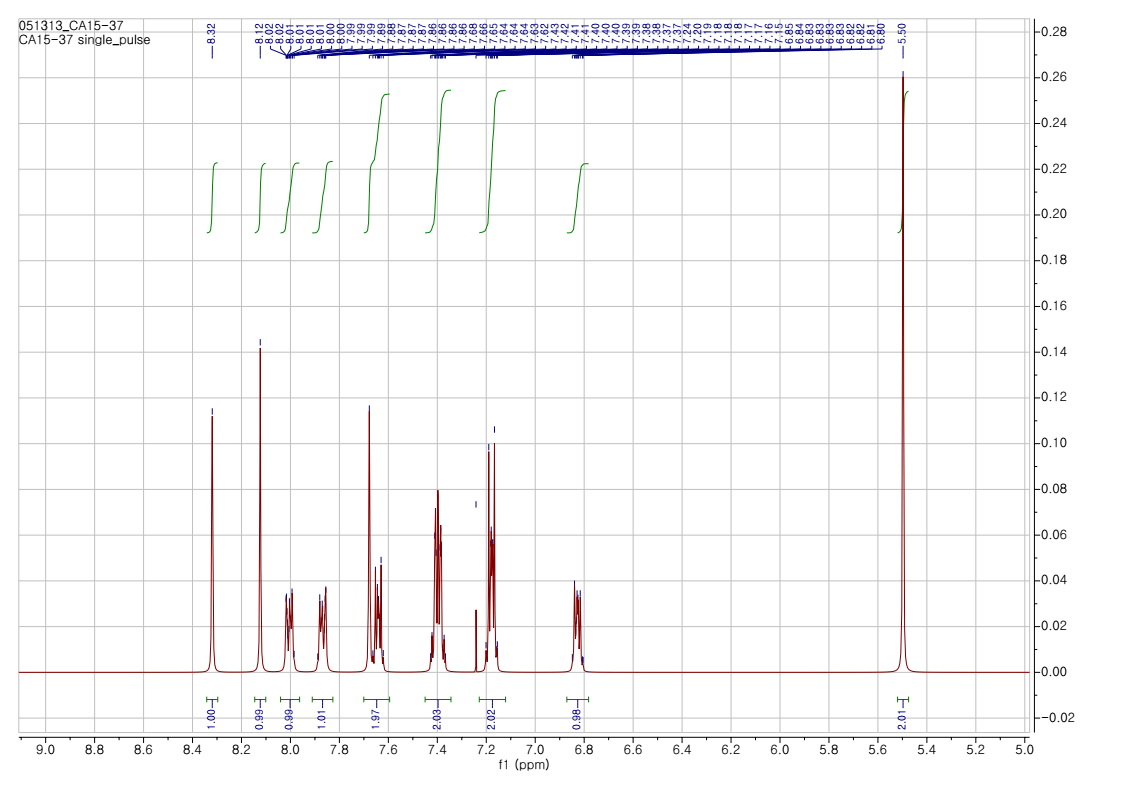

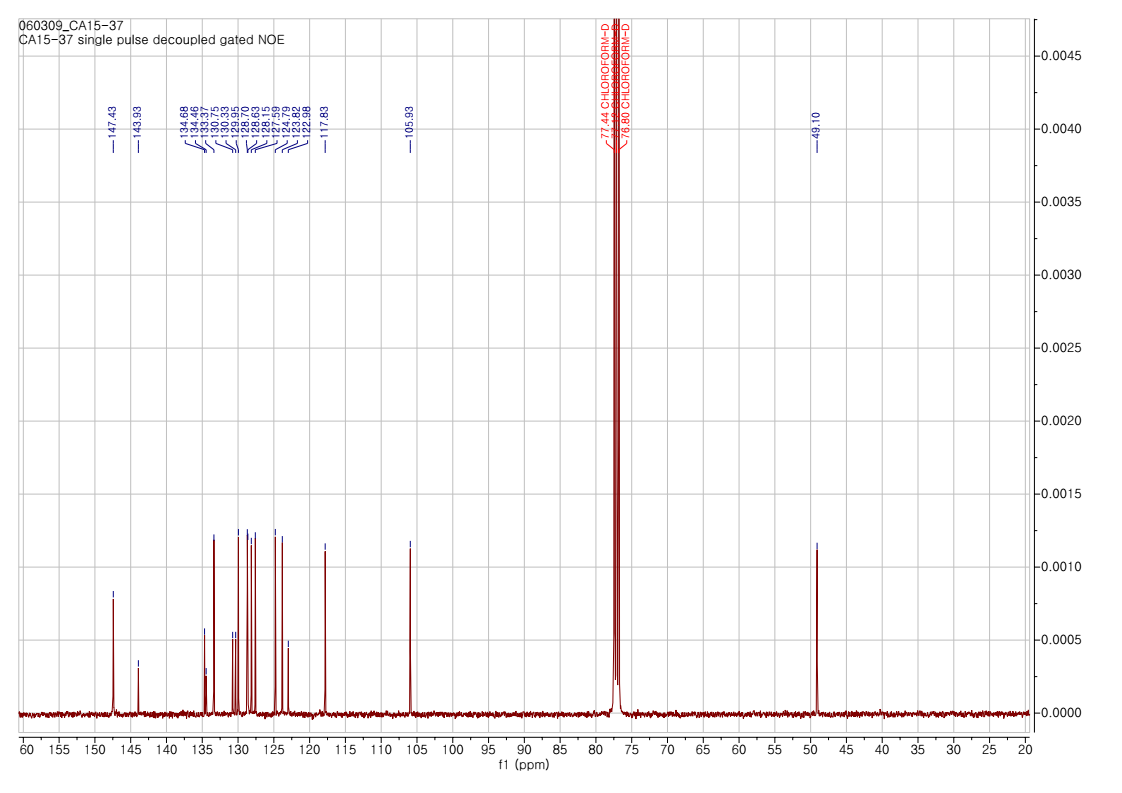


Figure S6. ^1^H-NMR and ^13^C-NMR spectra of **BI** in CDCl_3_



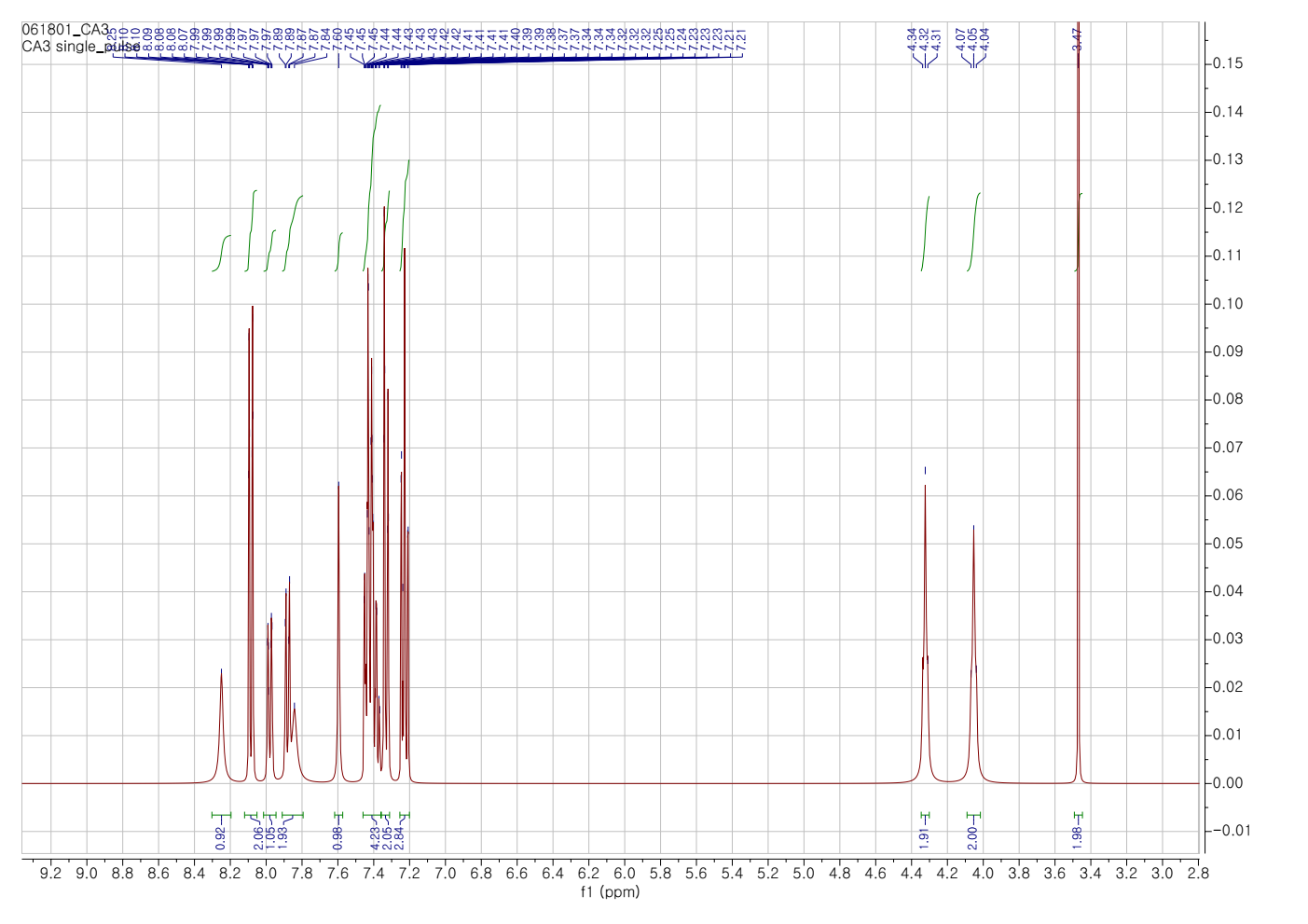

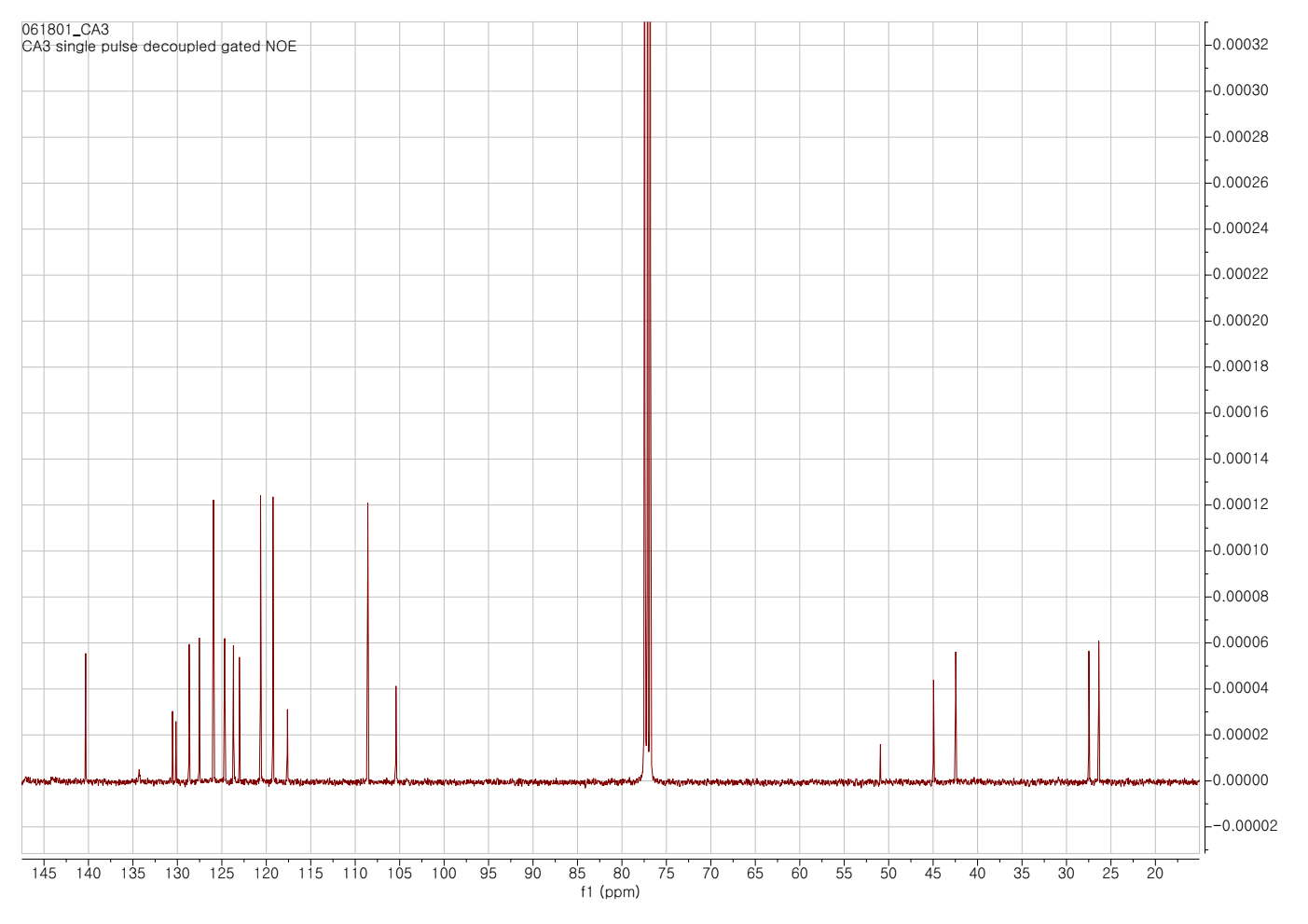


Figure S7. ^1^H-NMR and ^13^C-NMR spectra of **CI** in CDCl_3_



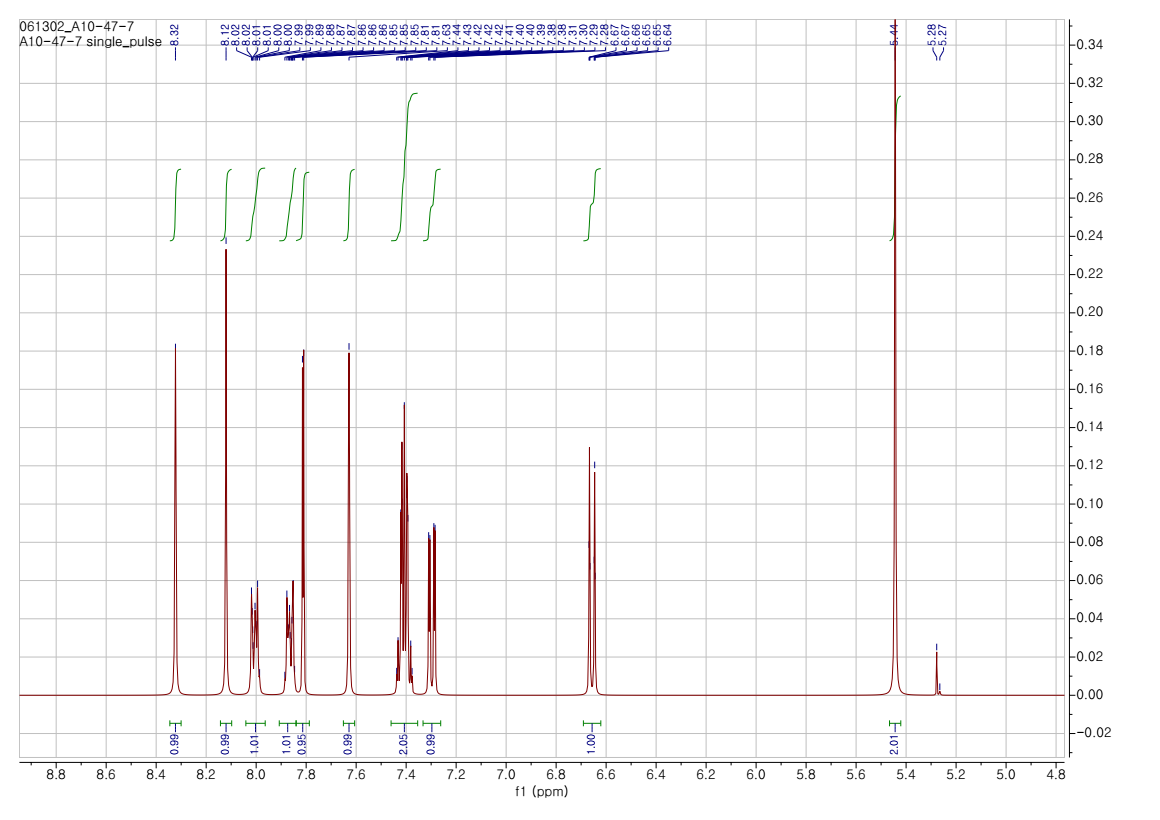

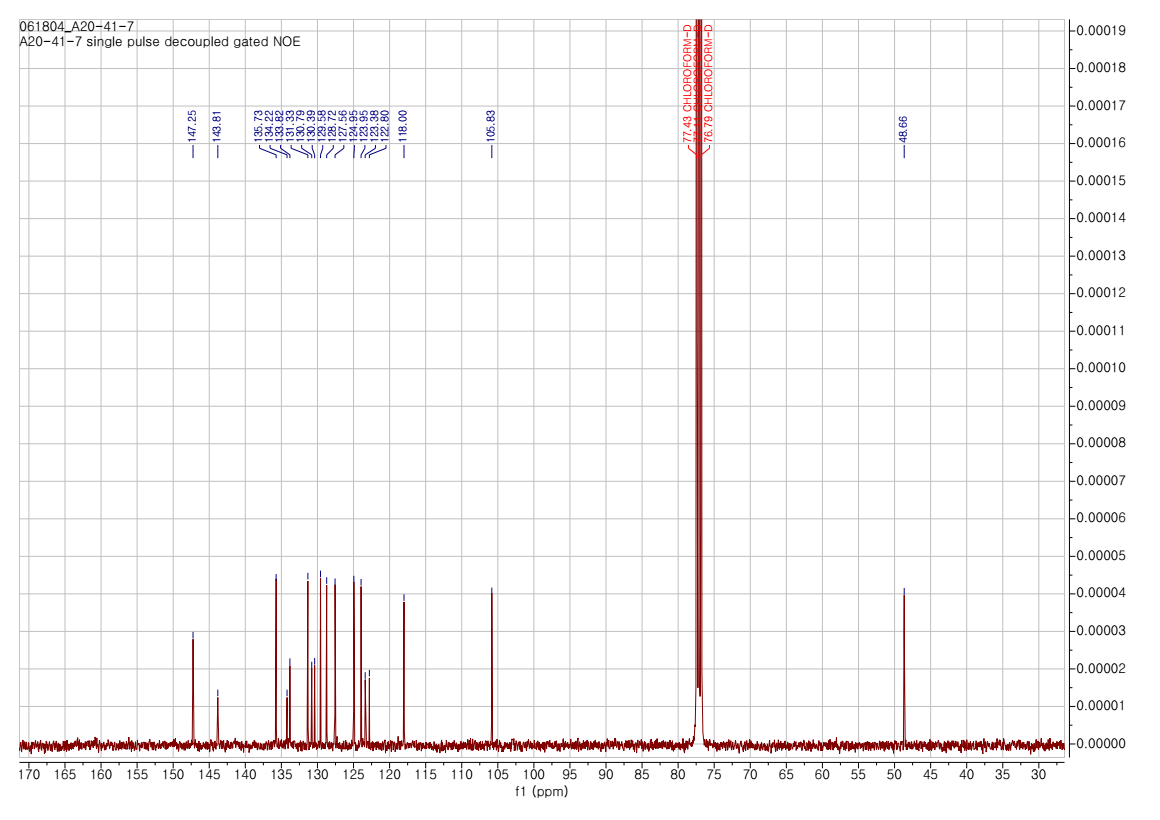


Figure S8. ^1^H-NMR and ^13^C-NMR spectra of **DI** in CDCl_3_



| a) Mass spectra of **DI**  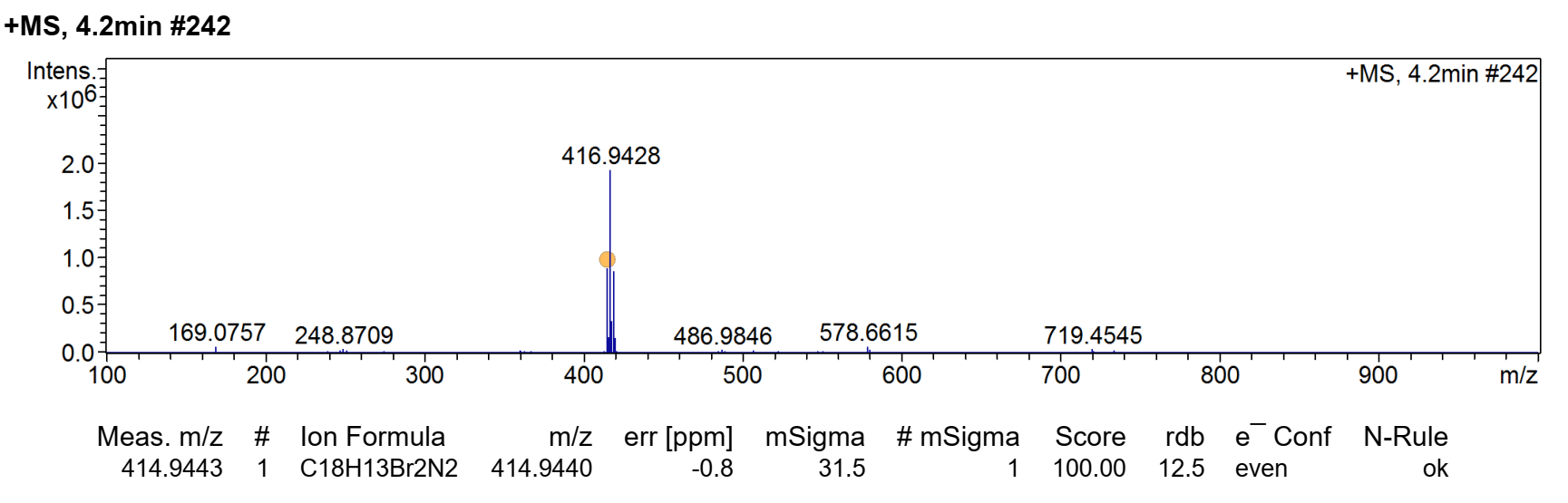 | a) Mass spectra of **BIB**  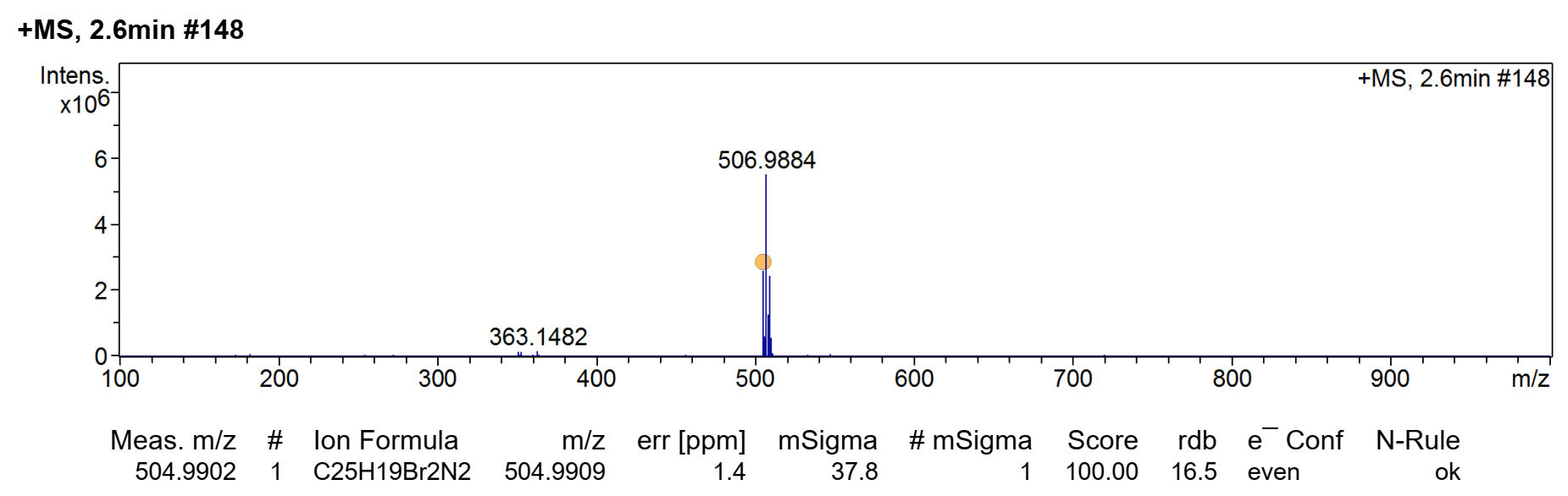 |
| --- | --- |

Figue S9. Mass spectra of (a) **DI** and (b) **BIB**


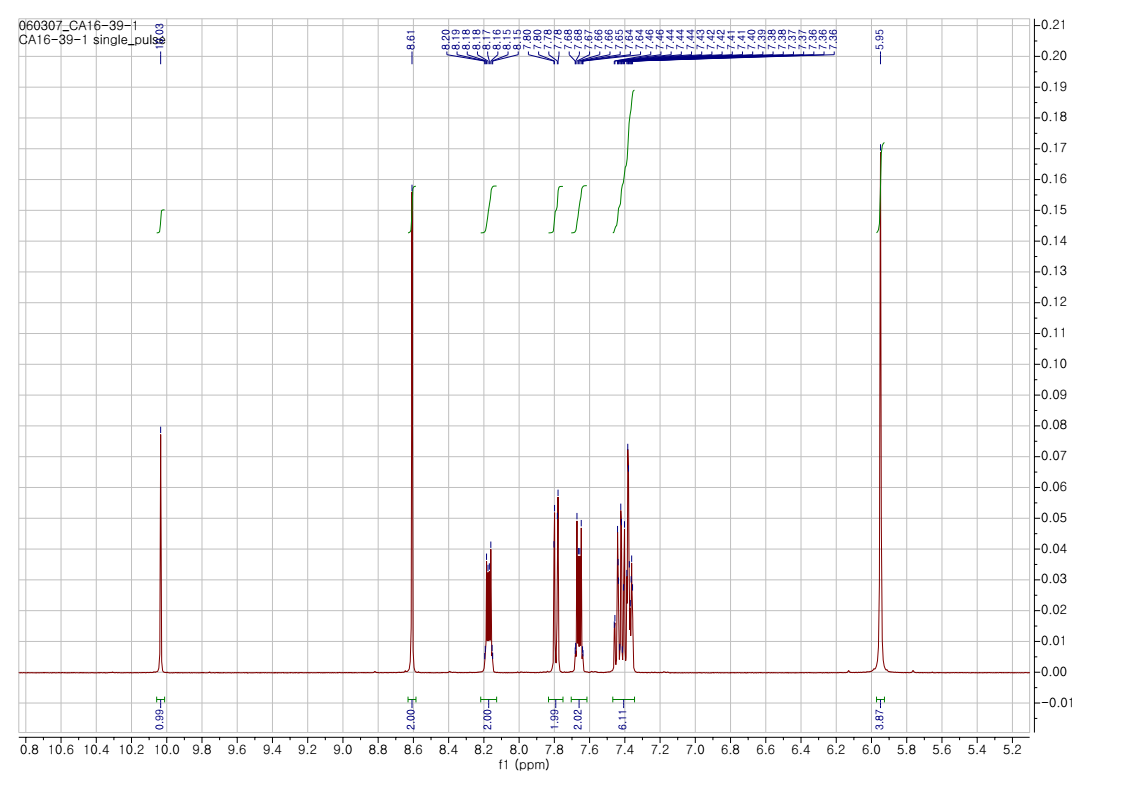

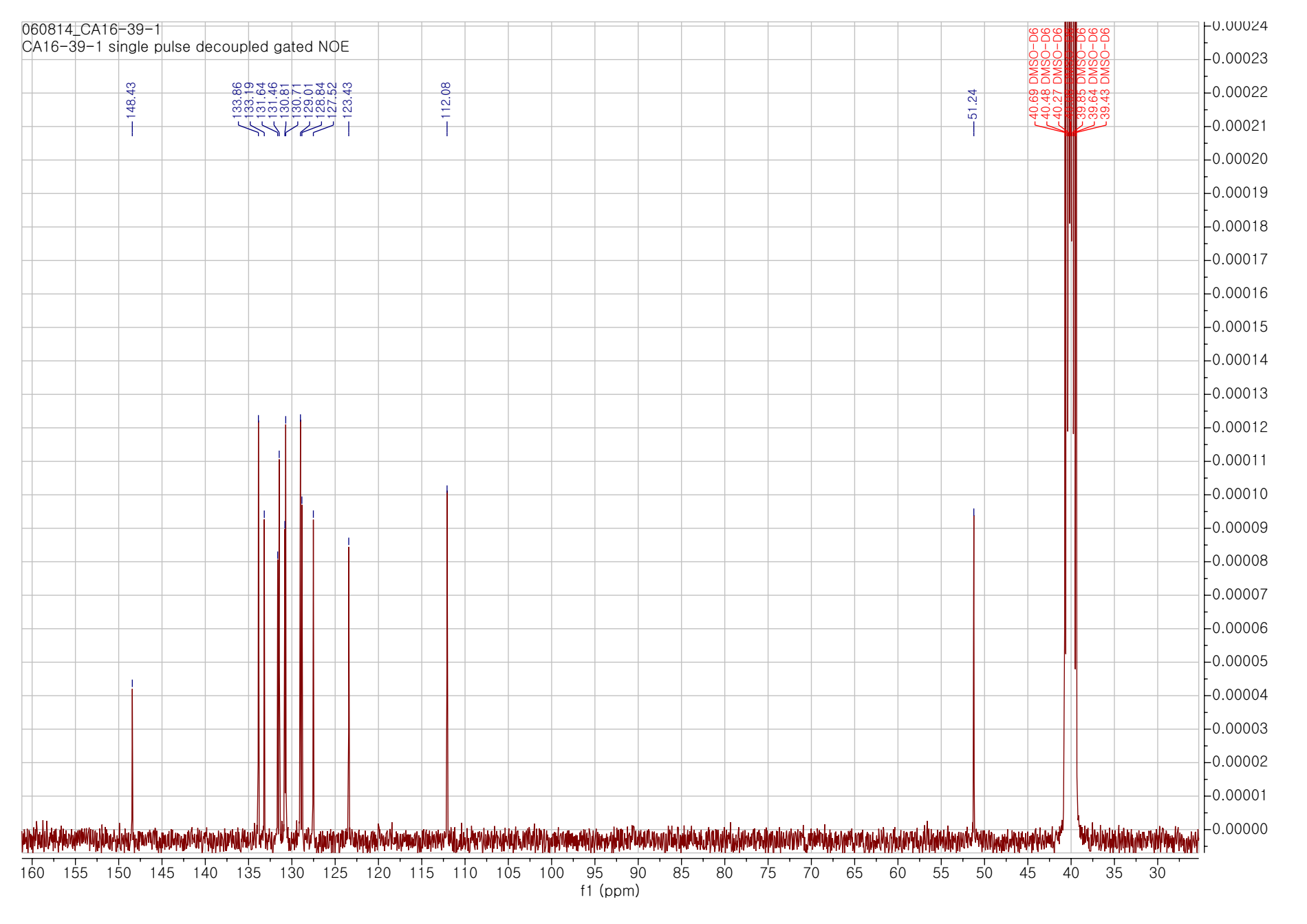


Figure S10. ^1^H-NMR and ^13^C-NMR spectra of **BIB** in DMSO


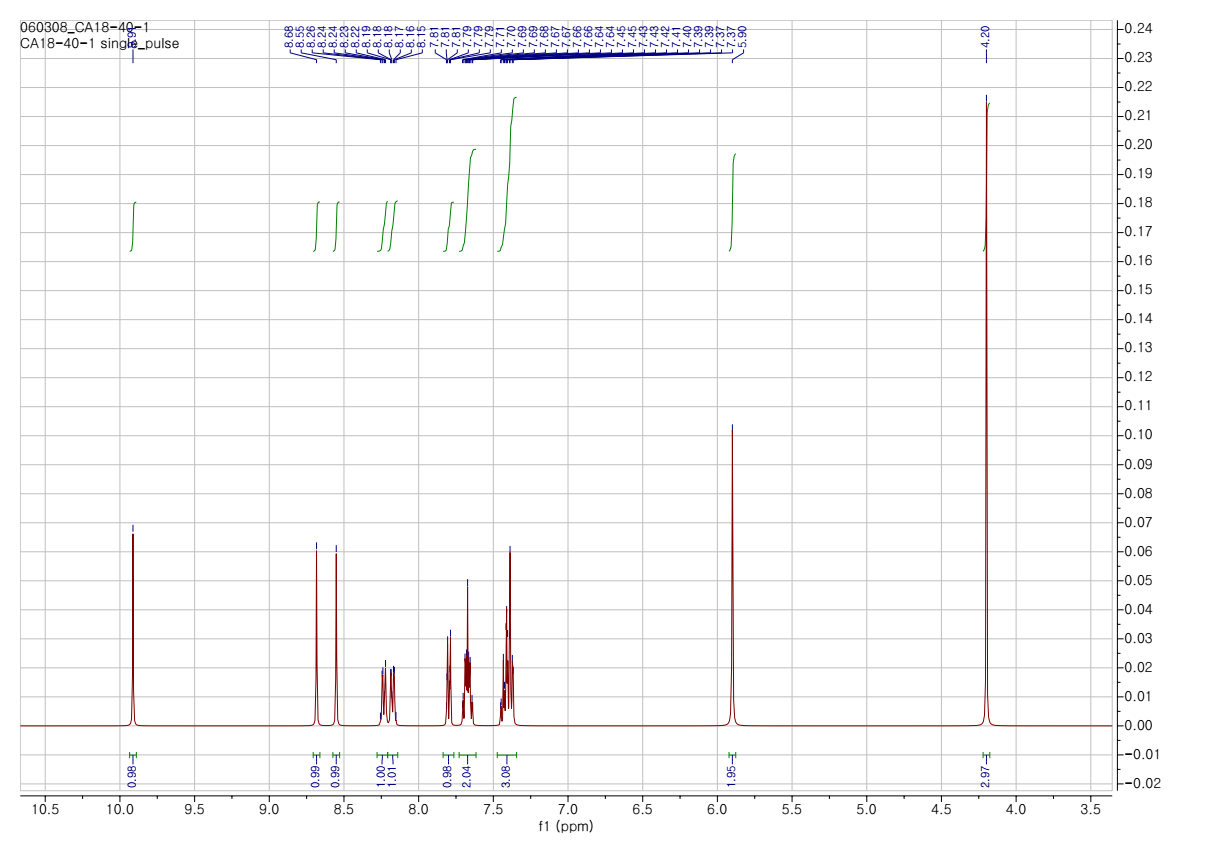

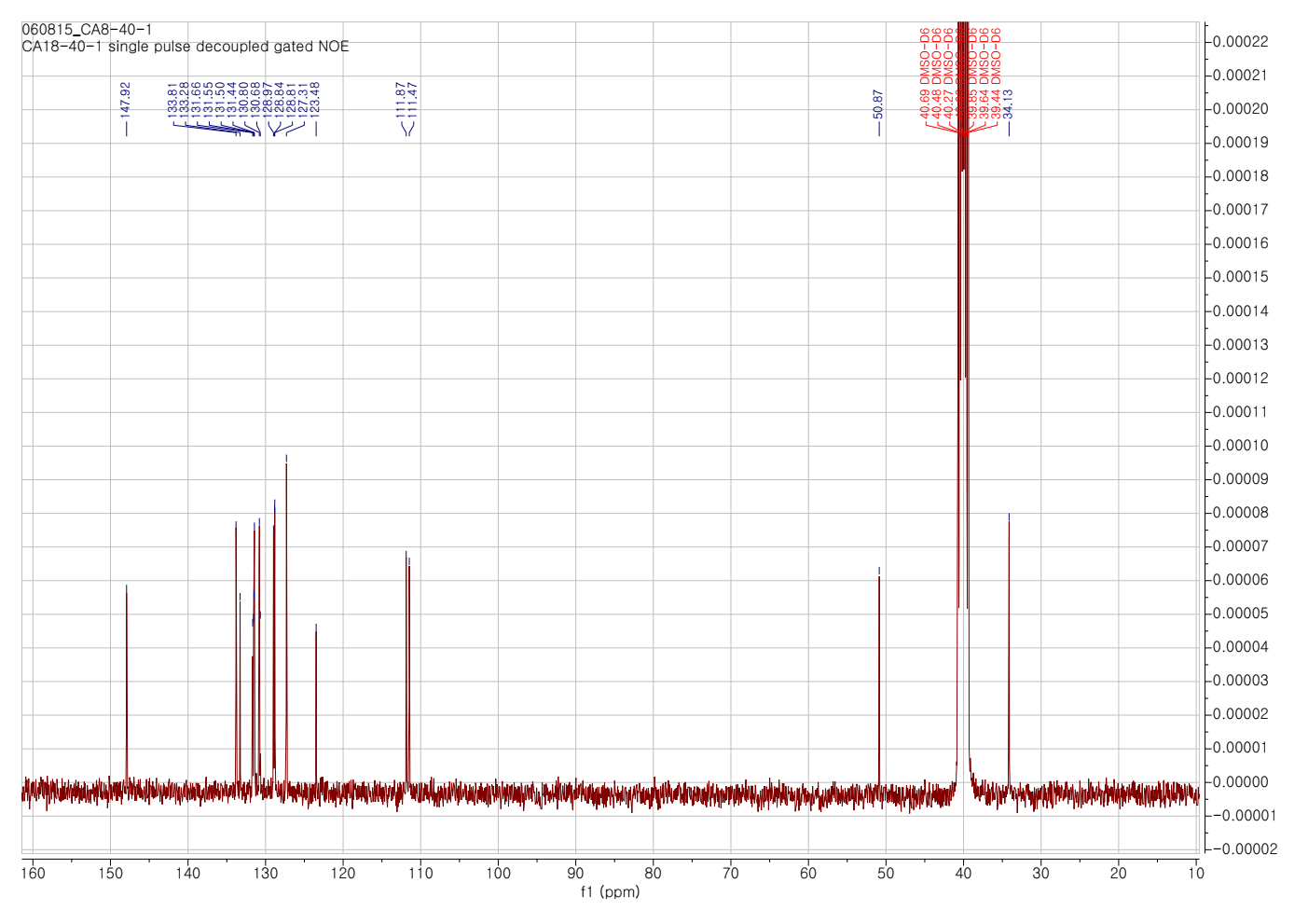


Figure S11. ^1^H-NMR and ^13^C-NMR spectra of **BIM** in DMSO



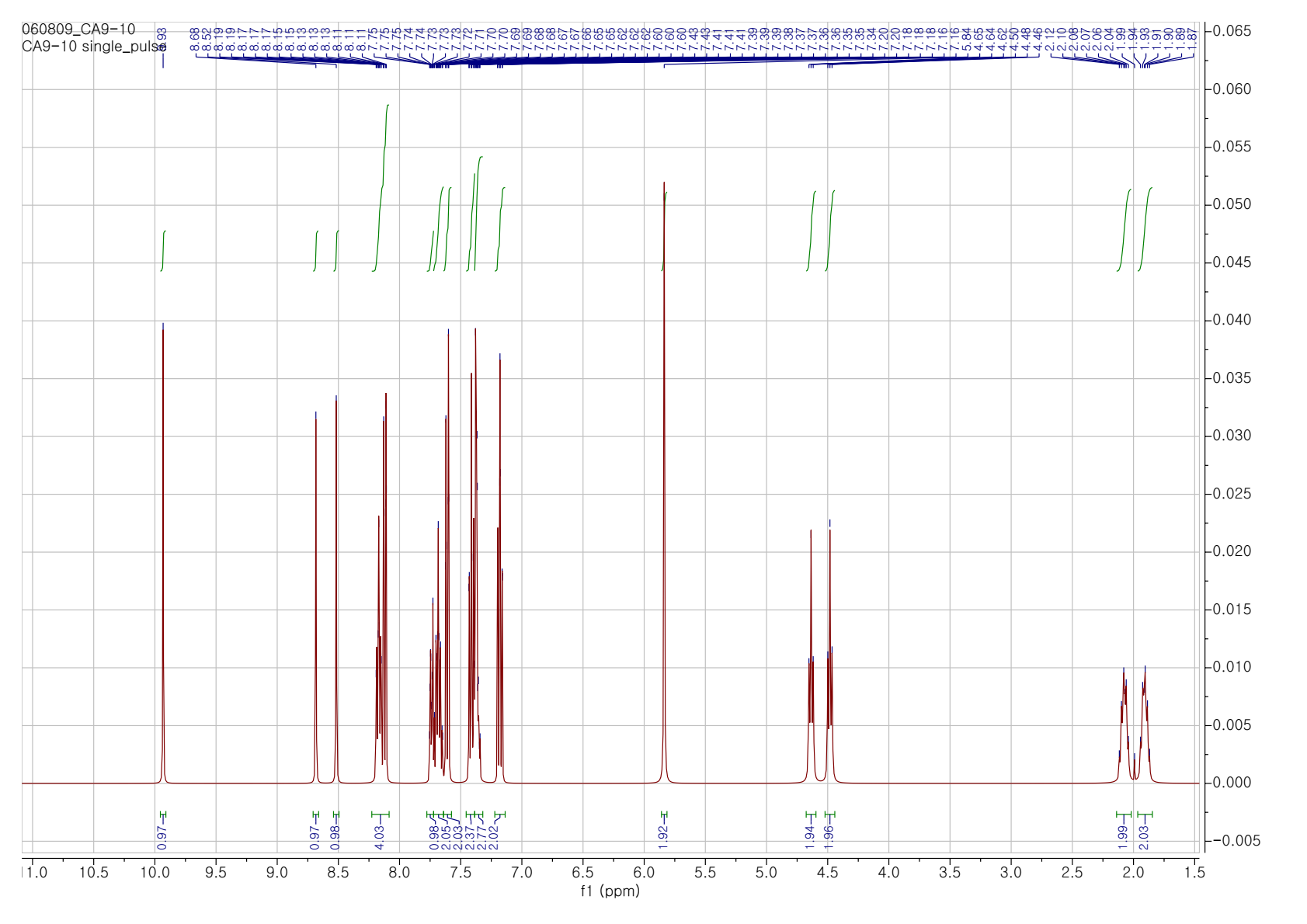

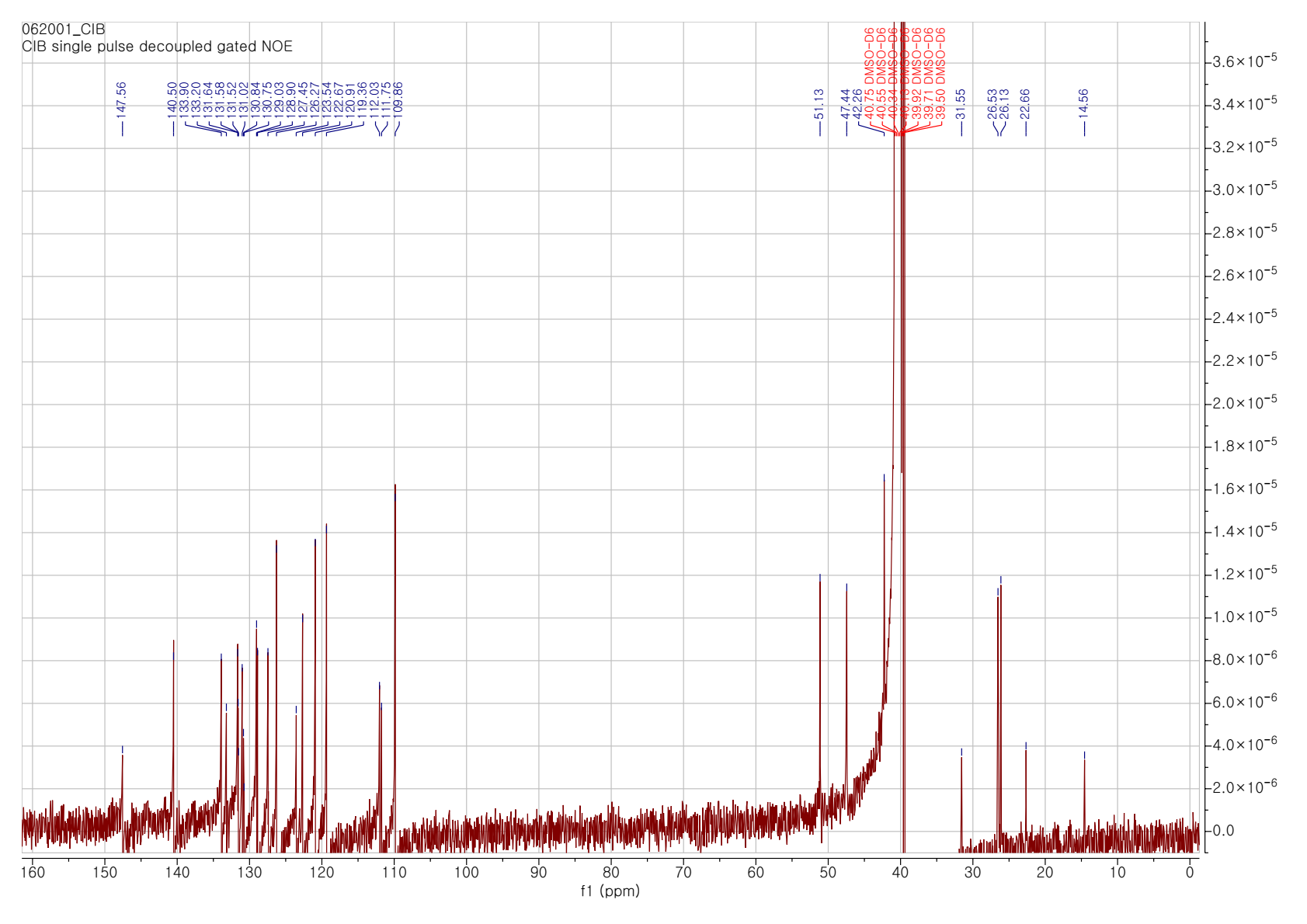


Figure S12. ^1^H-NMR and ^13^C-NMR spectra of **CIB** in DMSO



| a) Mass spectra of **BIM**  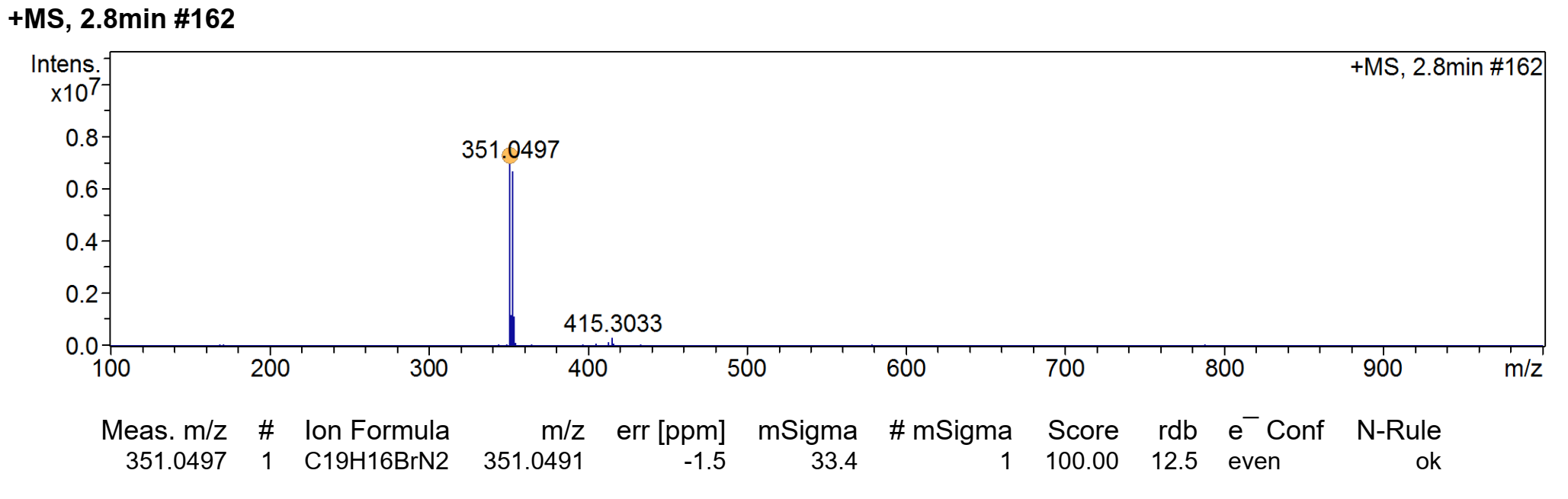 | b) Mass spectra of **CIB**  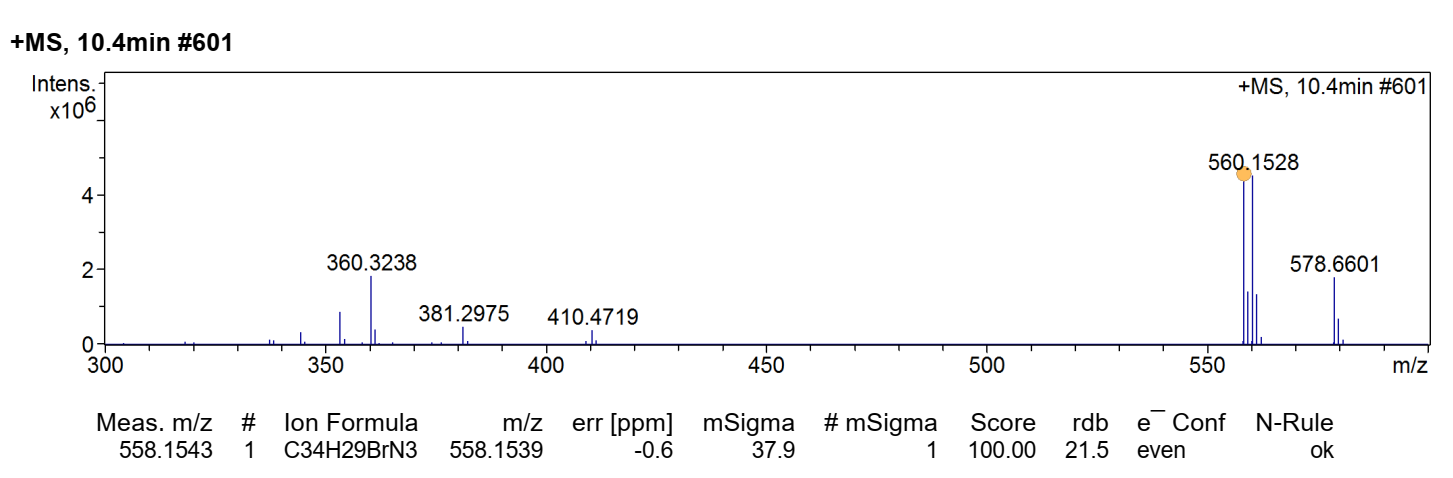 |
| --- | --- |

Figue S13. Mass spectra of (a) **BIM** and (b) **CIB**


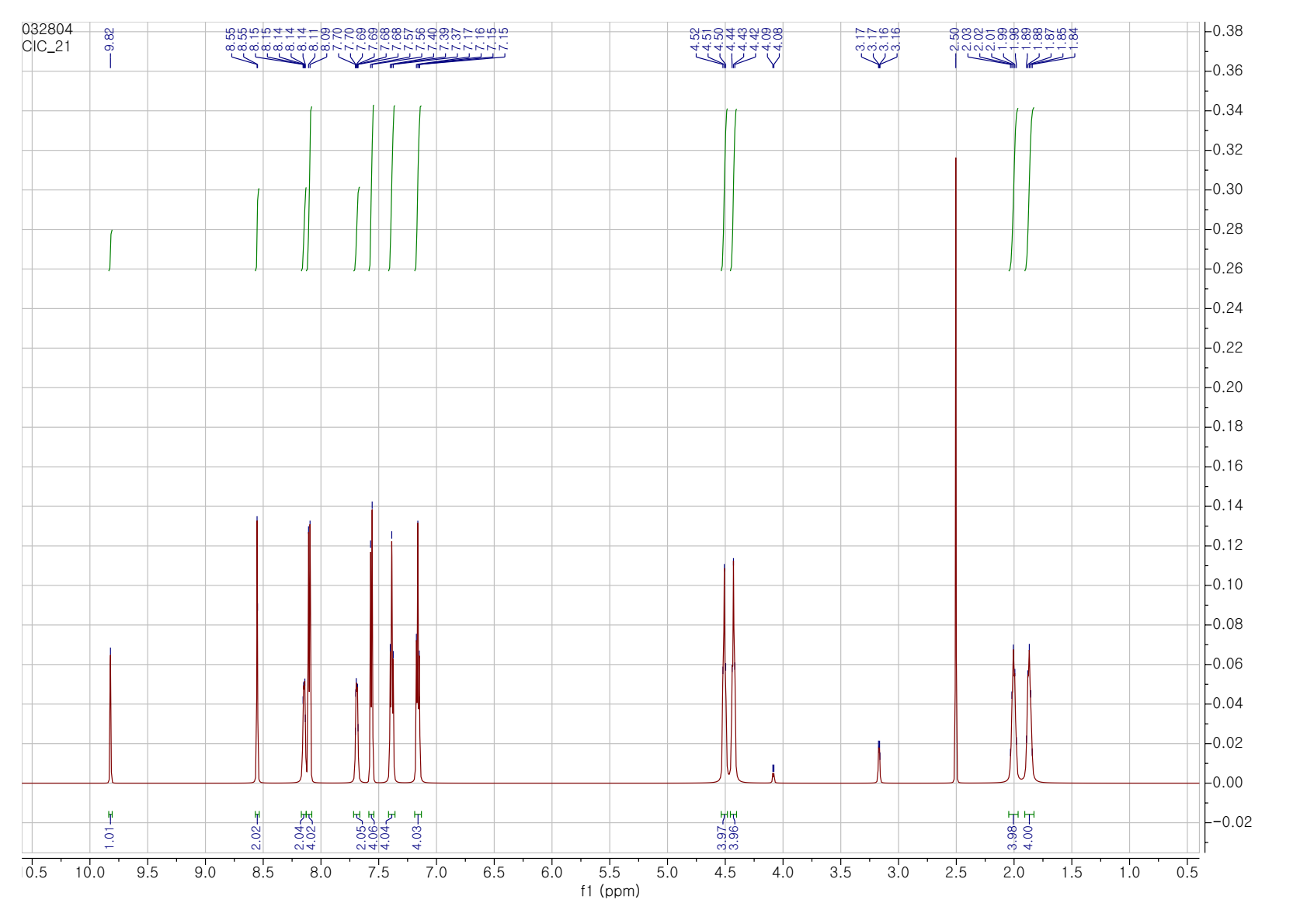

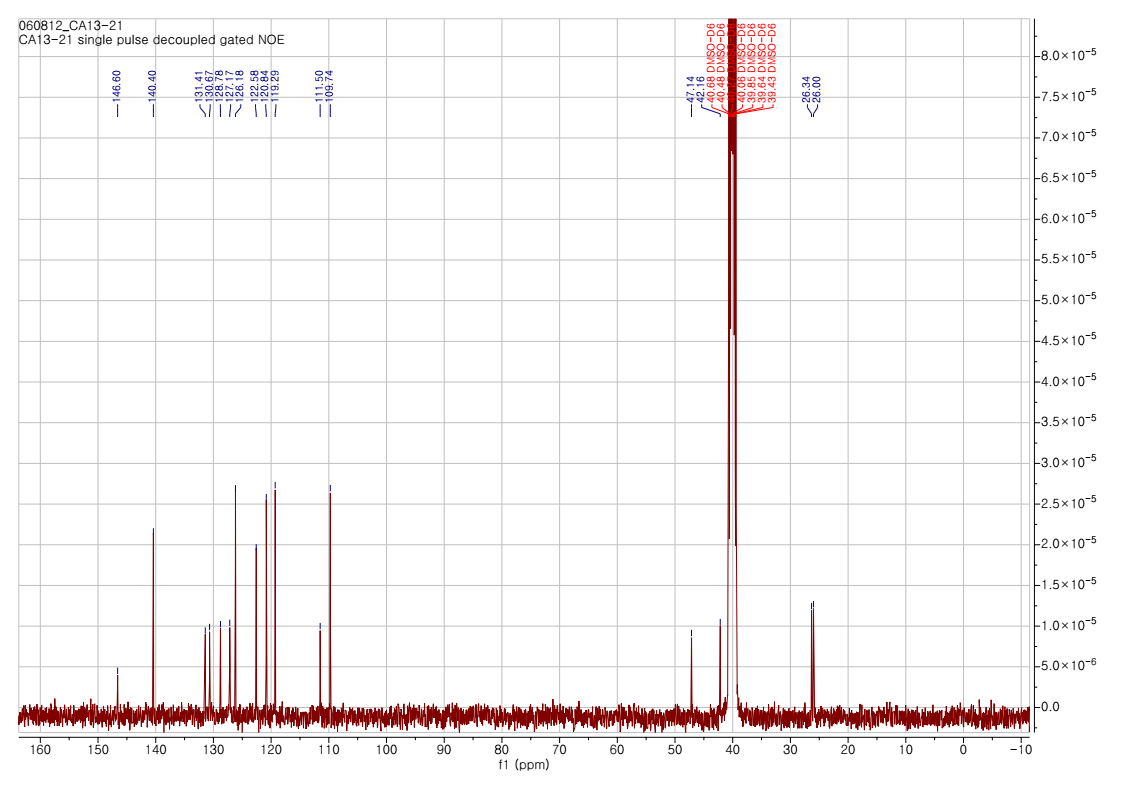


Figure S14. ^1^H-NMR and ^13^C-NMR spectra of **CIC** in DMSO



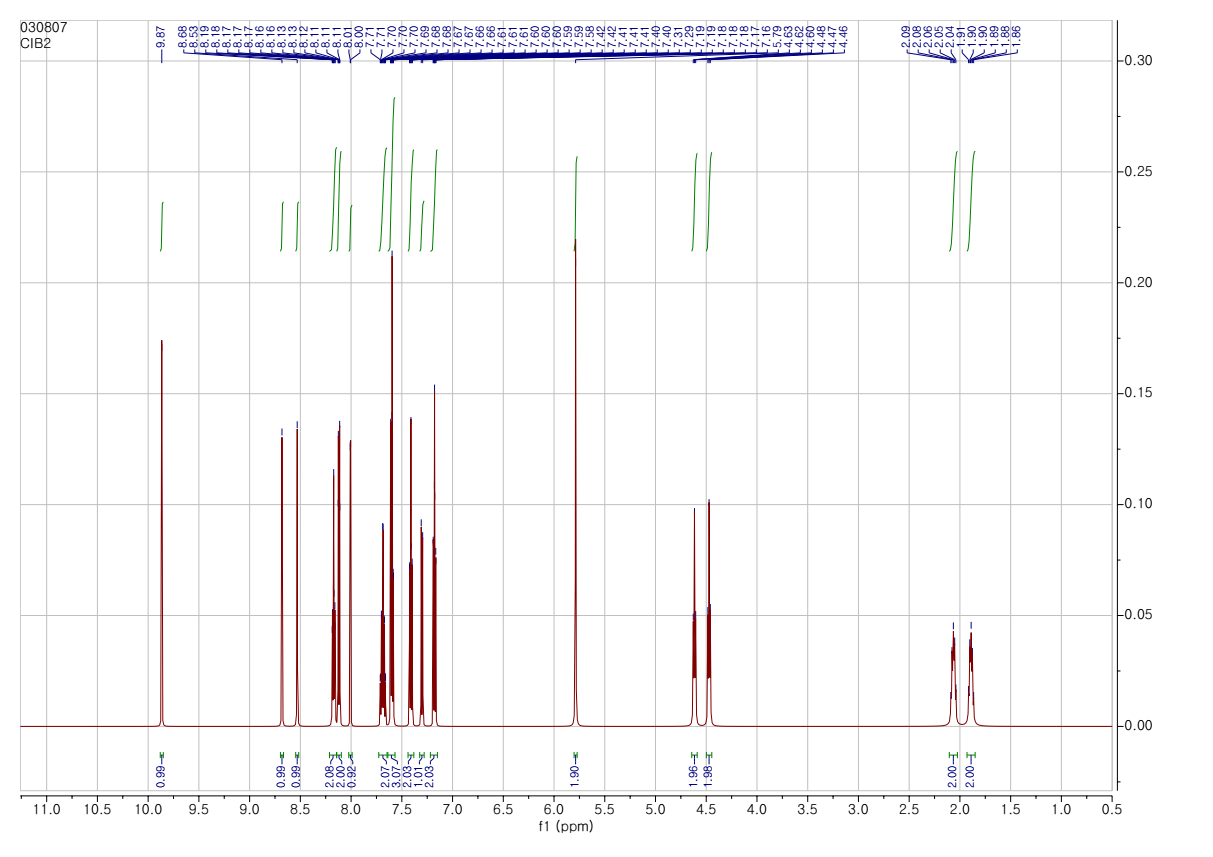

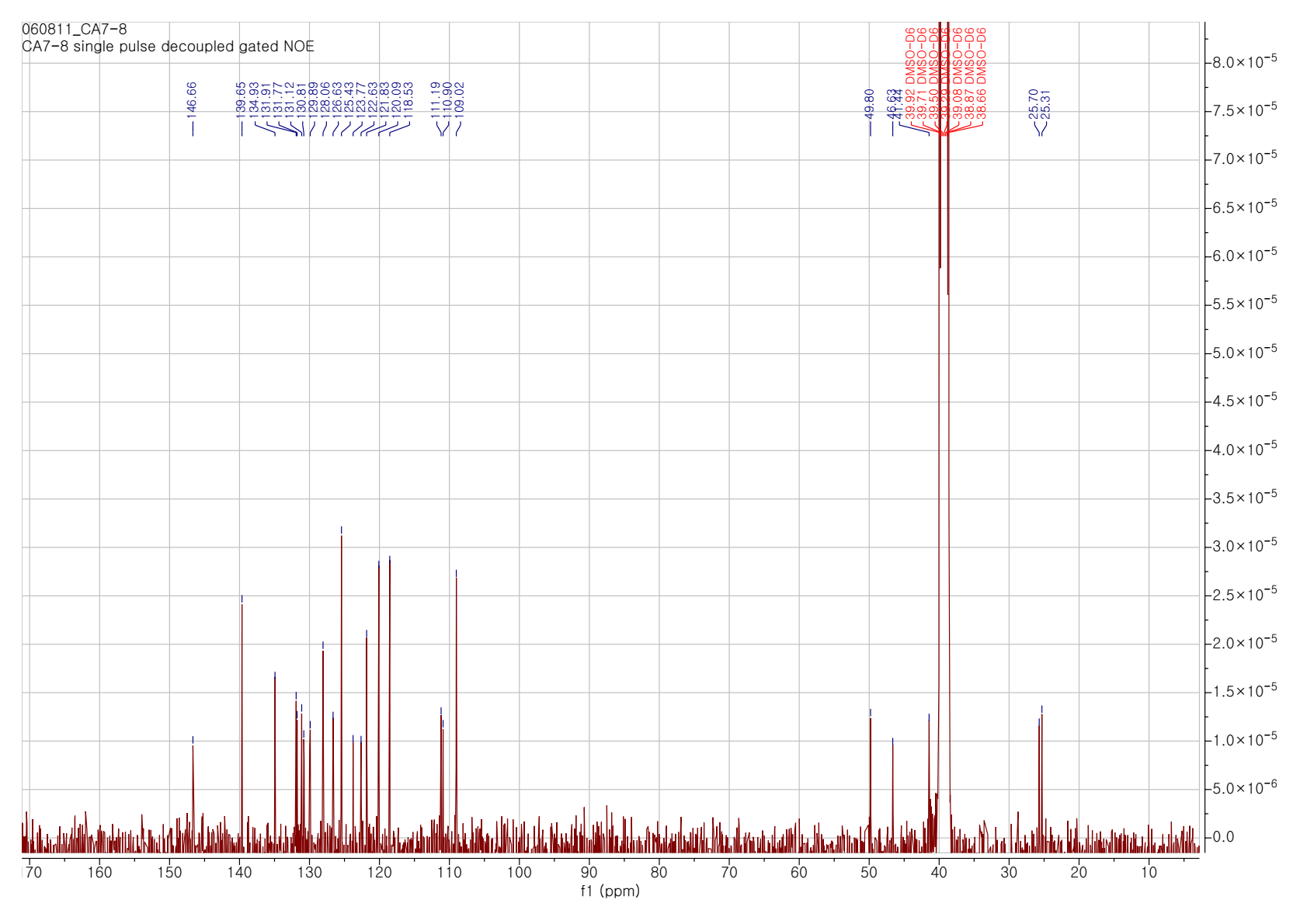


Figure S15. ^1^H-NMR and ^13^C-NMR spectra of **CID** in DMSO



| a) Mass spectra of **CIC**  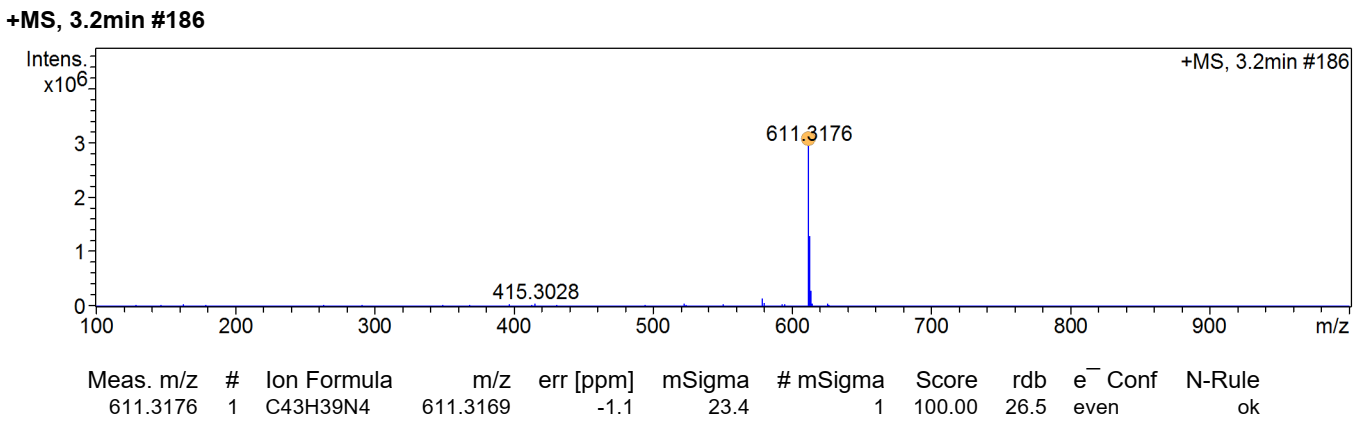 | b) Mass spectra of **CID**  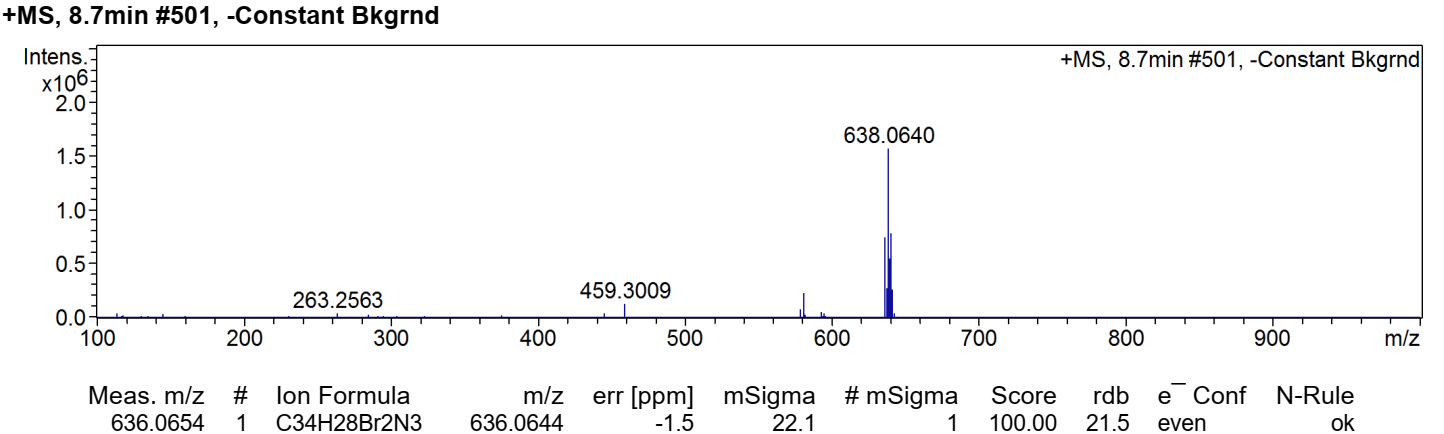 |
| --- | --- |
| c) Mass spectra of **CIM**  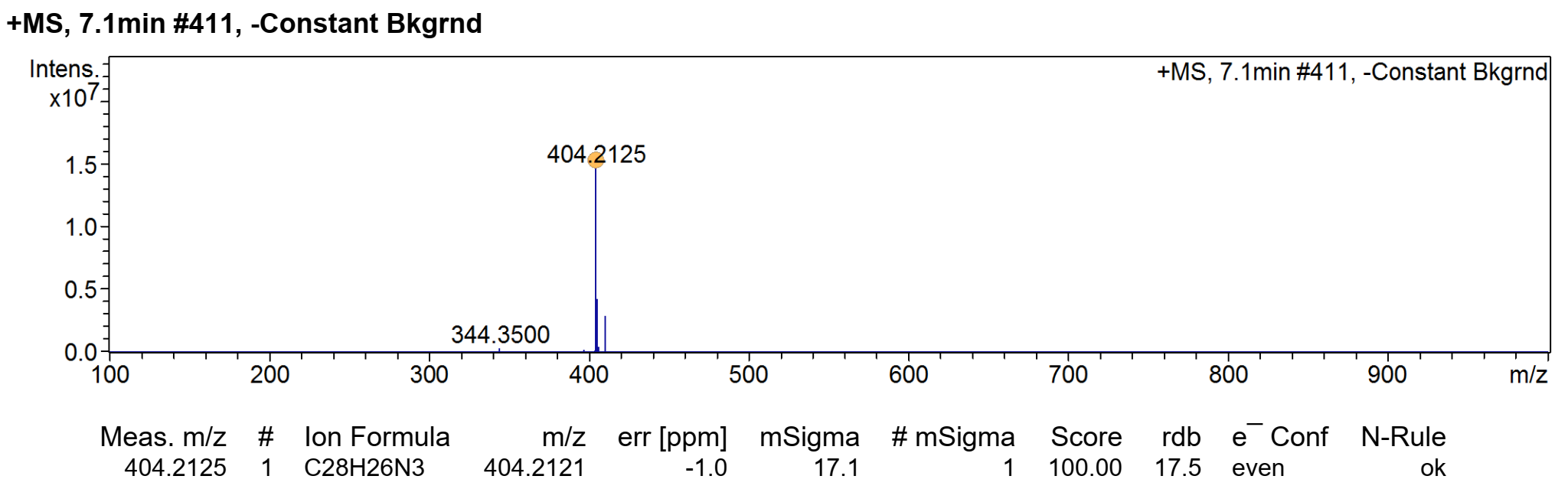 | d) Mass spectra of **DIB**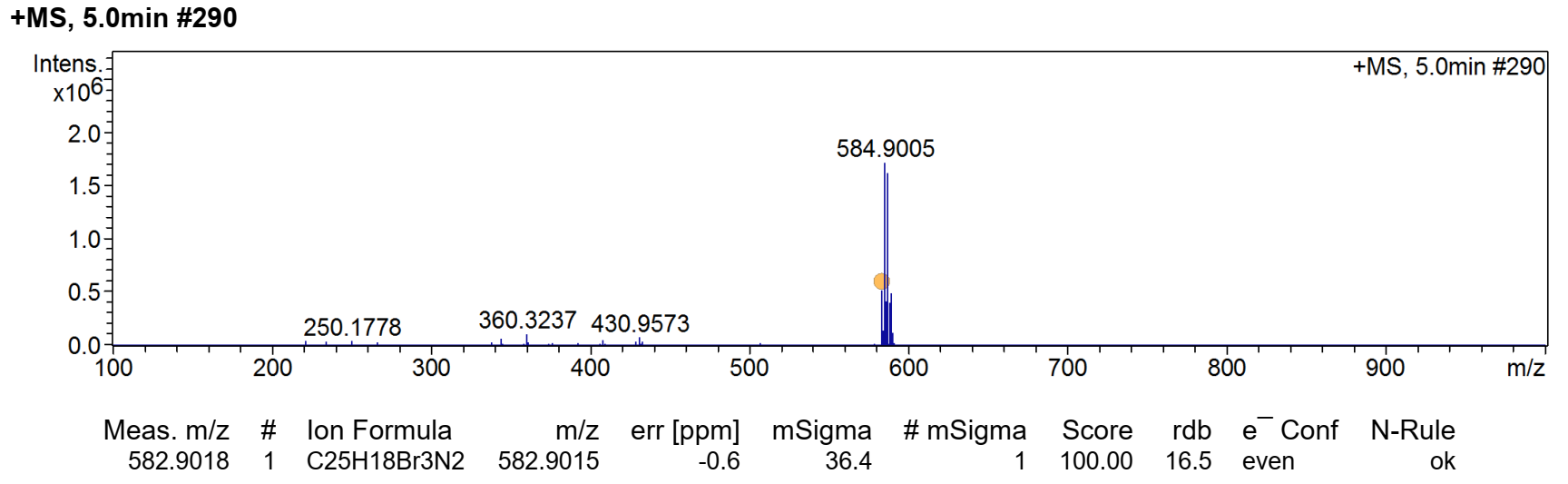 |

Figue S16. Mass spectra of (a) **CIC**, (b) **CIB,** (c) **CIM** and (d) **DIB.**


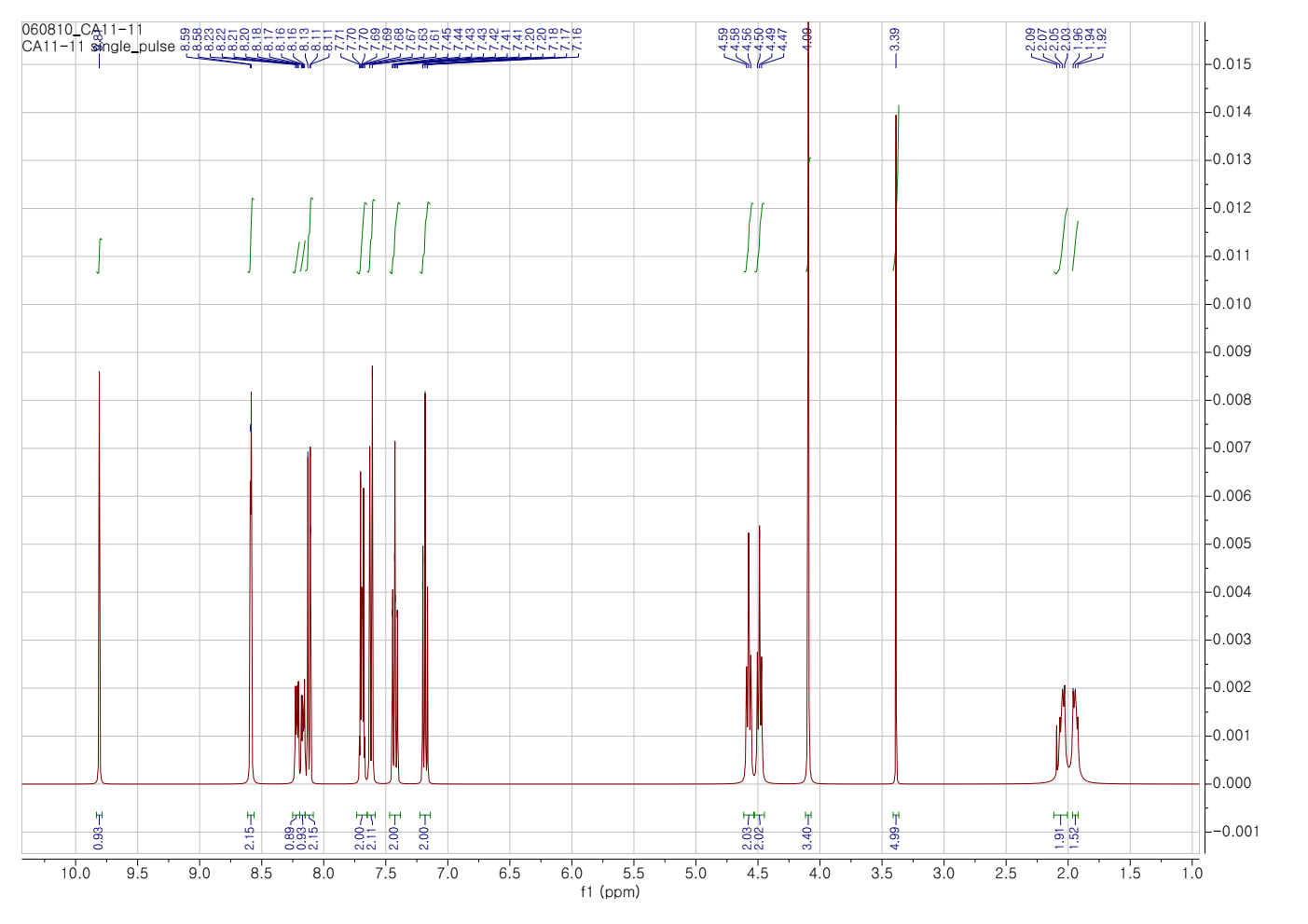

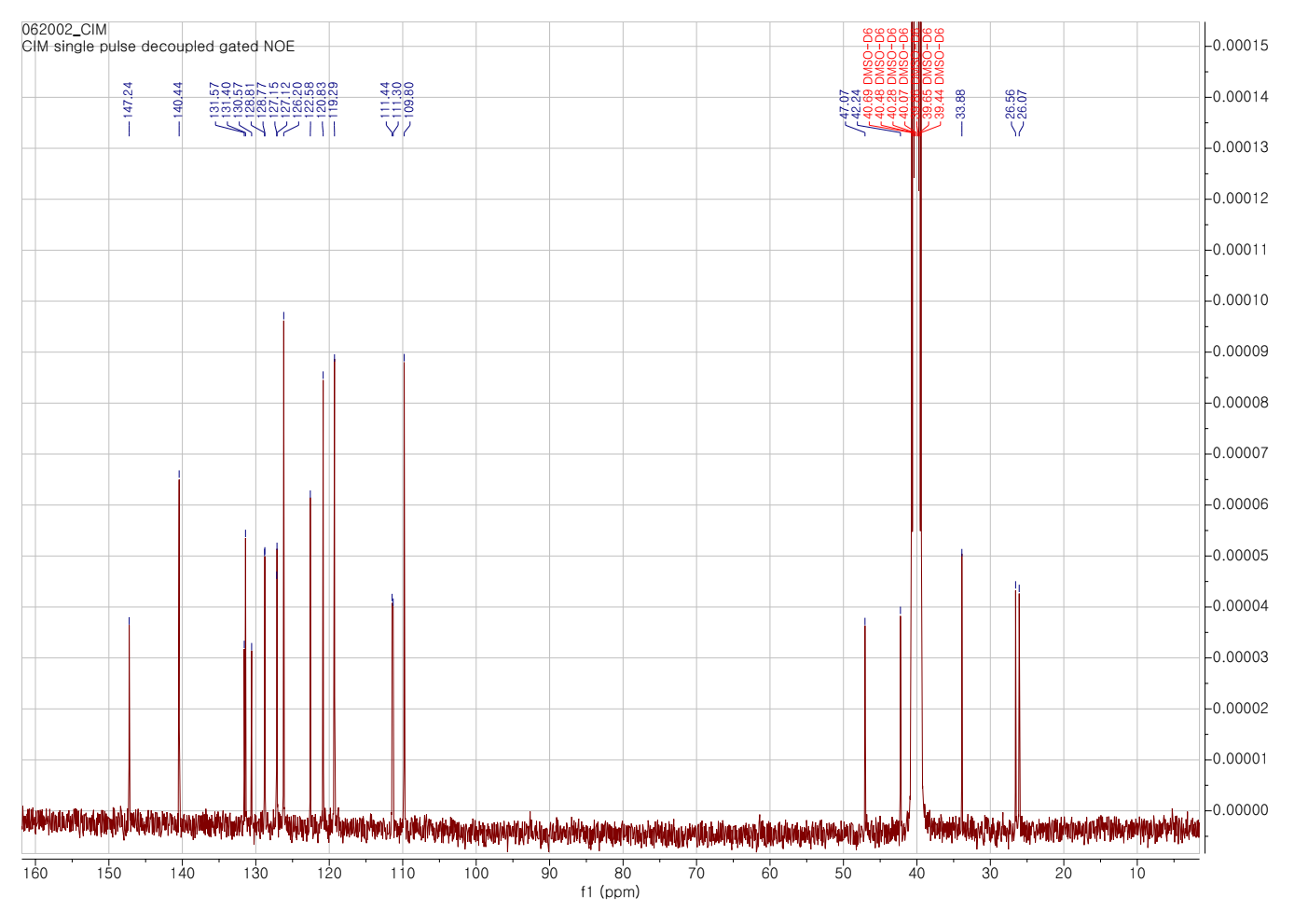


Figure S17. ^1^H-NMR and ^13^C-NMR spectra of **CIM** in DMSO



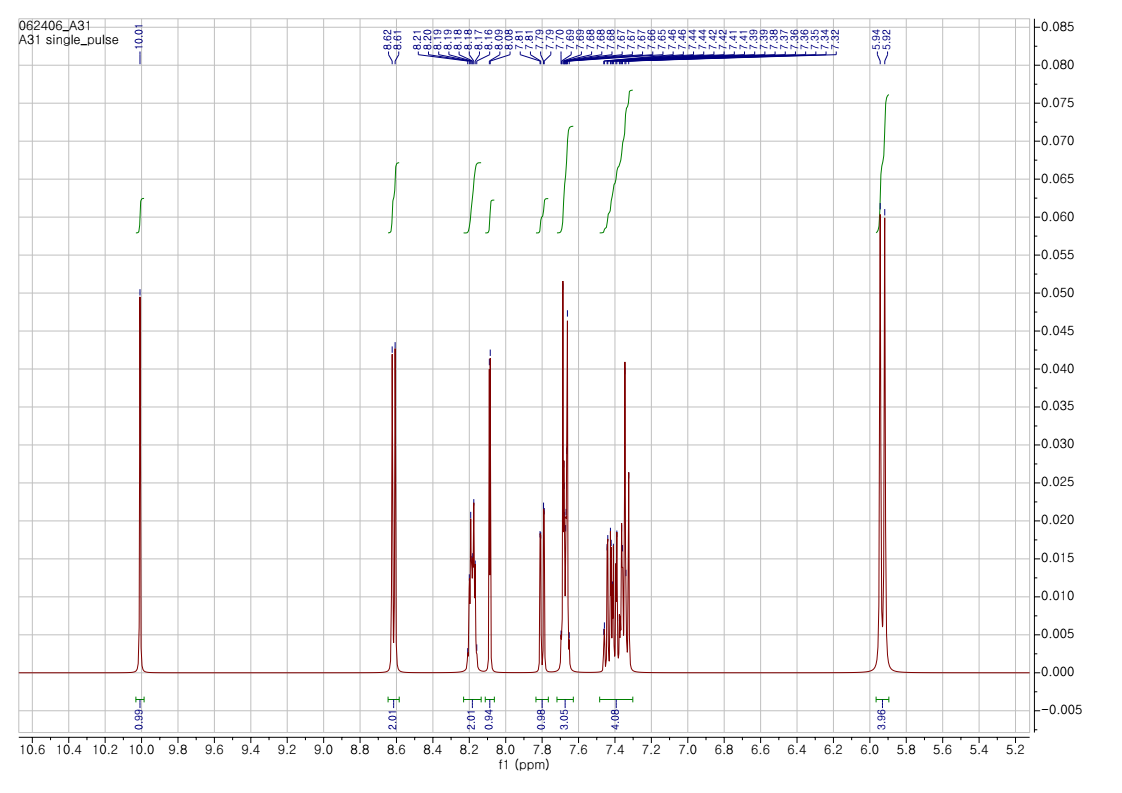

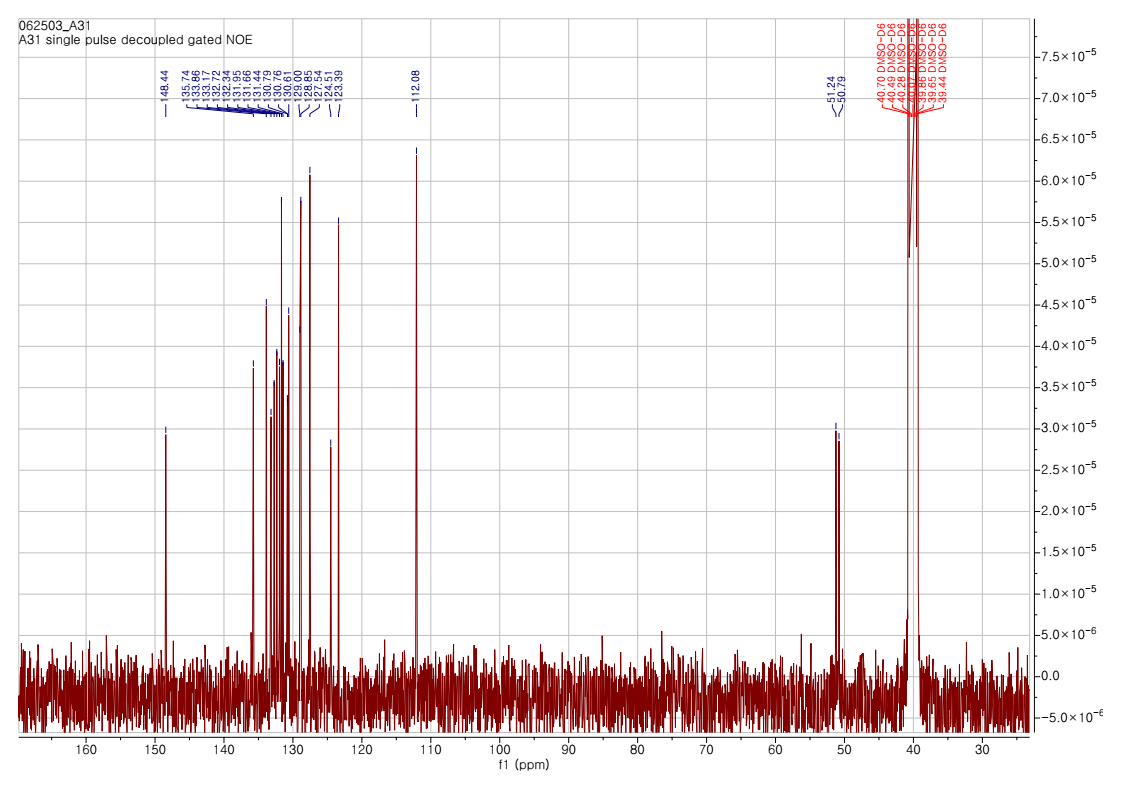


Figure S18. ^1^H-NMR and ^13^C-NMR spectra of **DIB** in DMSO



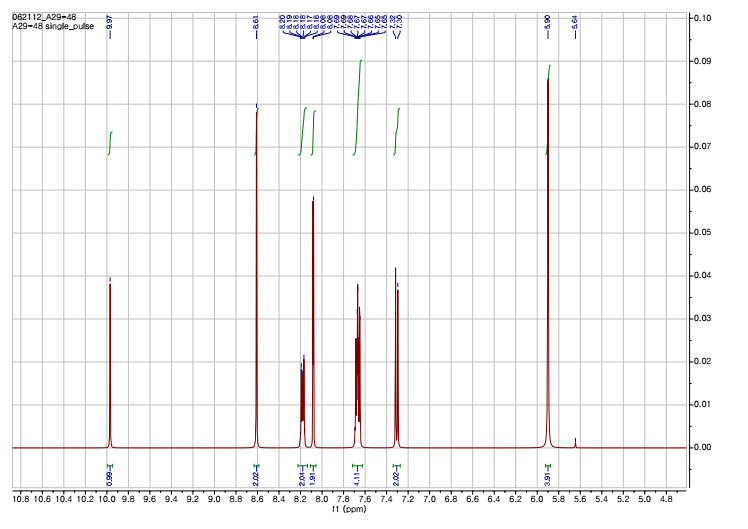

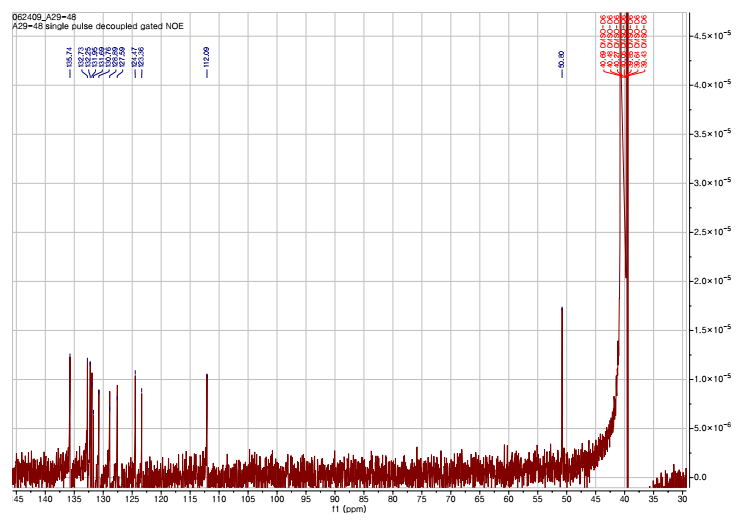


Figure S19. ^1^H-NMR and ^13^C-NMR spectra of **DID** in DMSO



| a) Mass spectra of **DID**  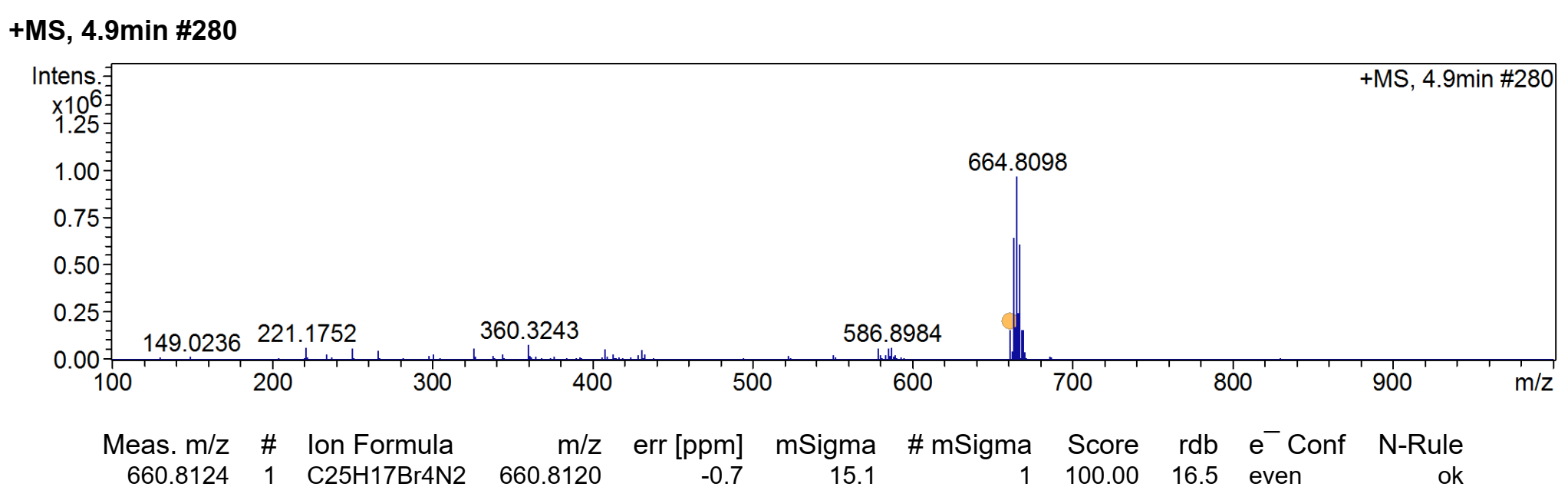 | b) Mass spectra of **DIM**  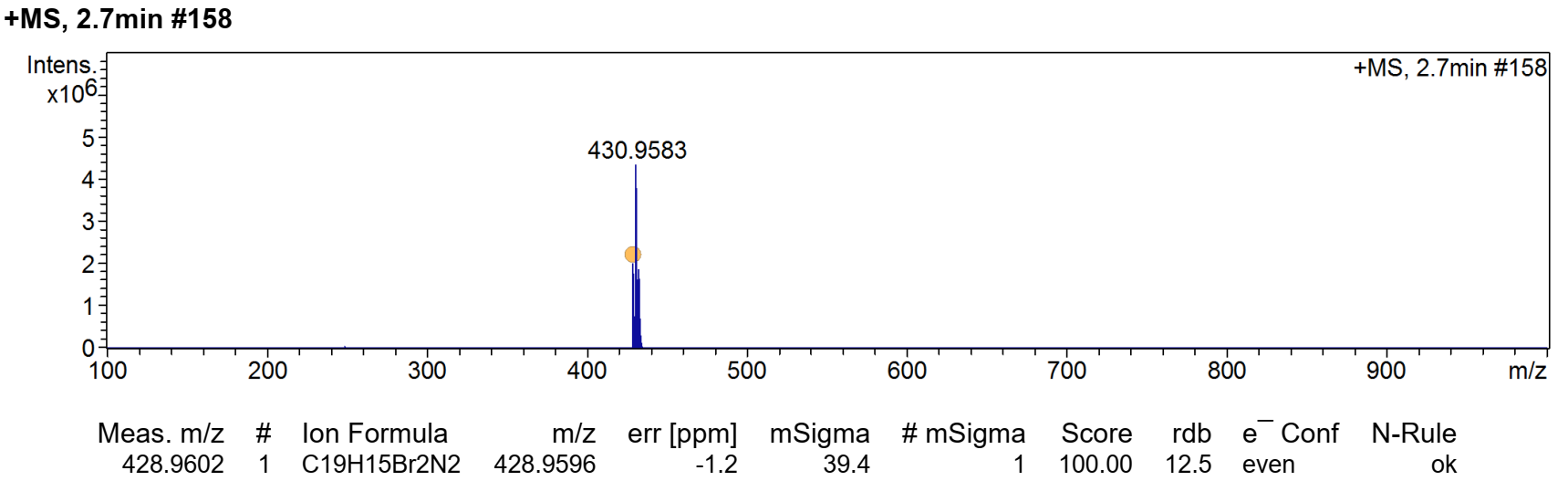 |
| --- | --- |

Figure S20. Mass spectra of (a) **DID** and (b) **DIM**.


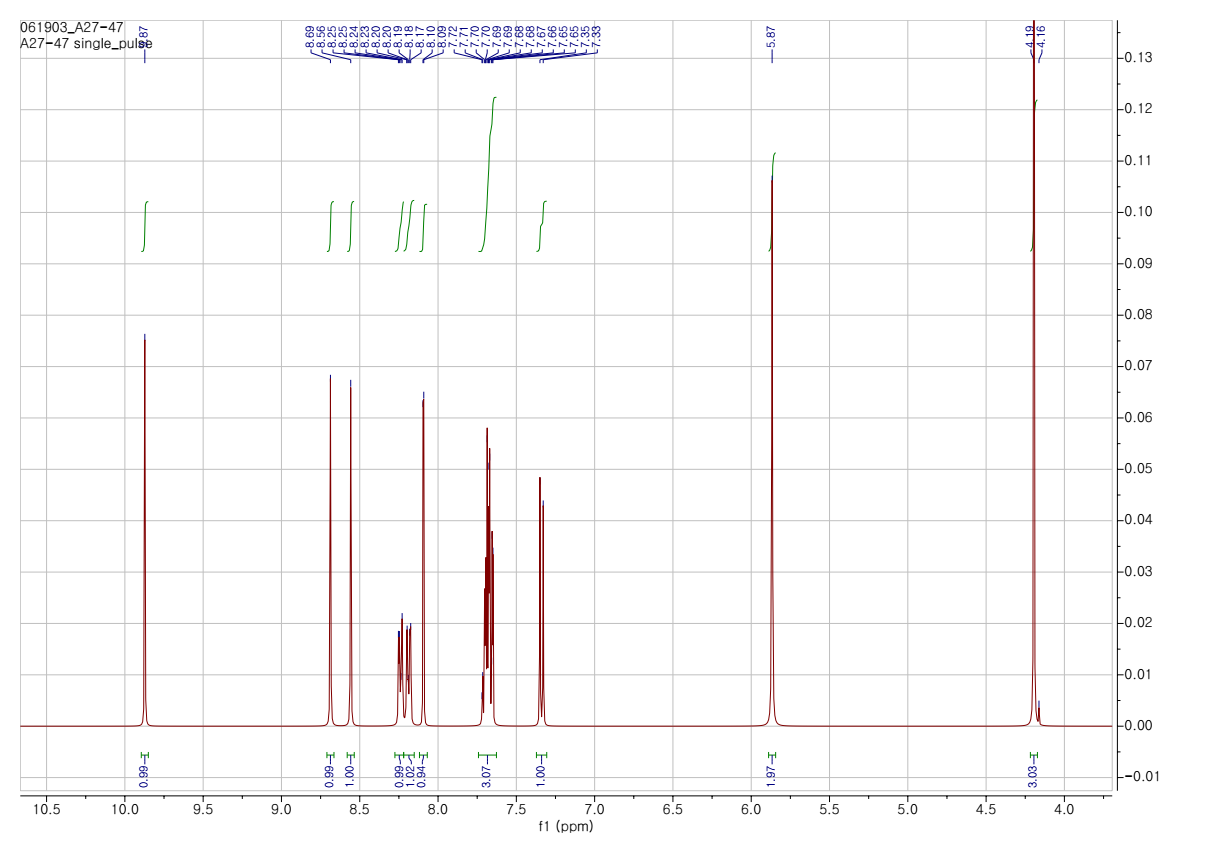

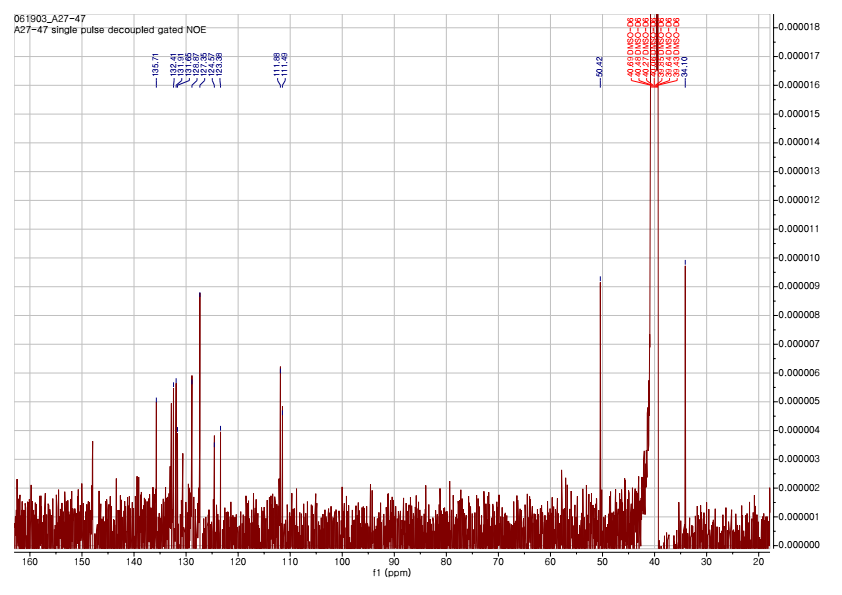


Figure S21. ^1^H-NMR and ^13^C-NMR spectra of **DIM** in DMSO



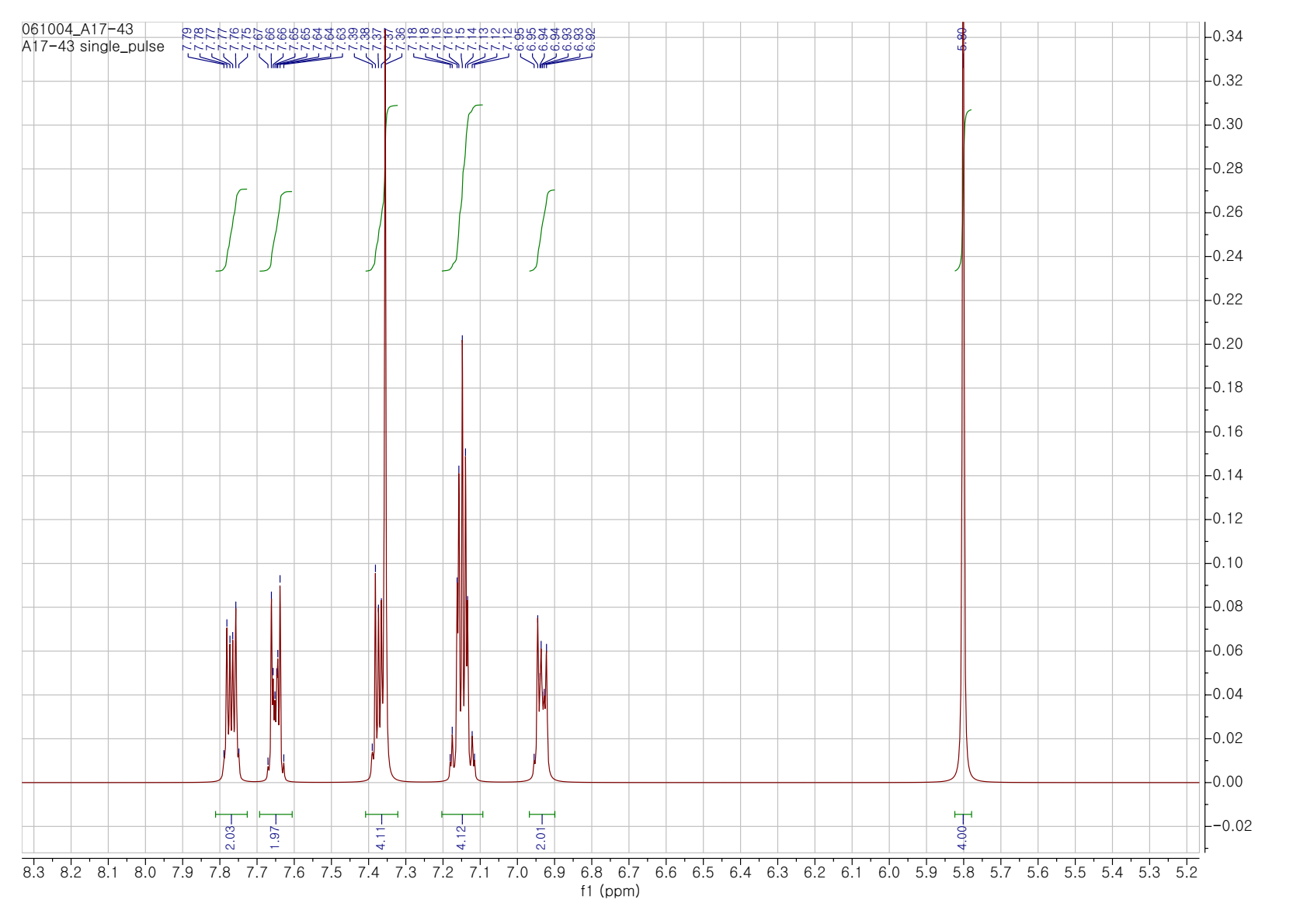

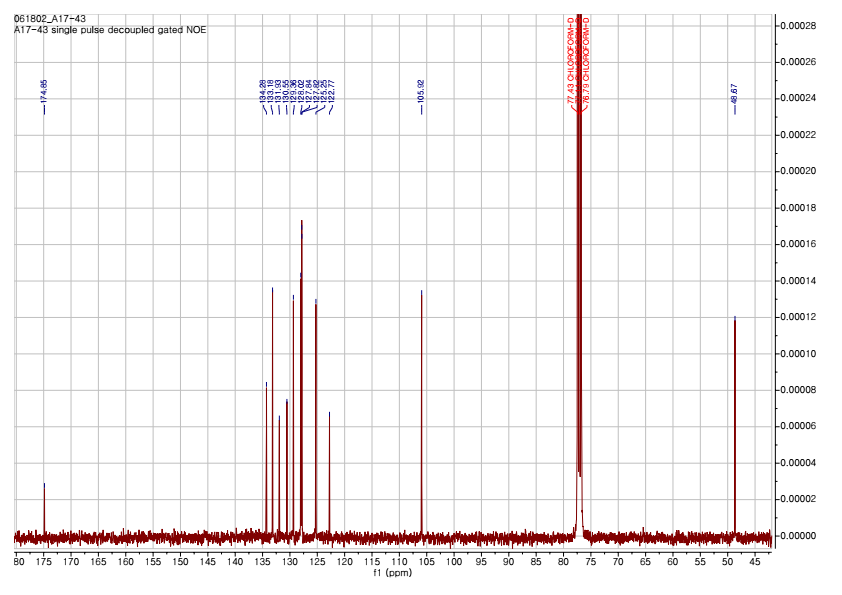


Figure S22. ^1^H-NMR and ^13^C-NMR spectra of **BSB** in CHCl_3_



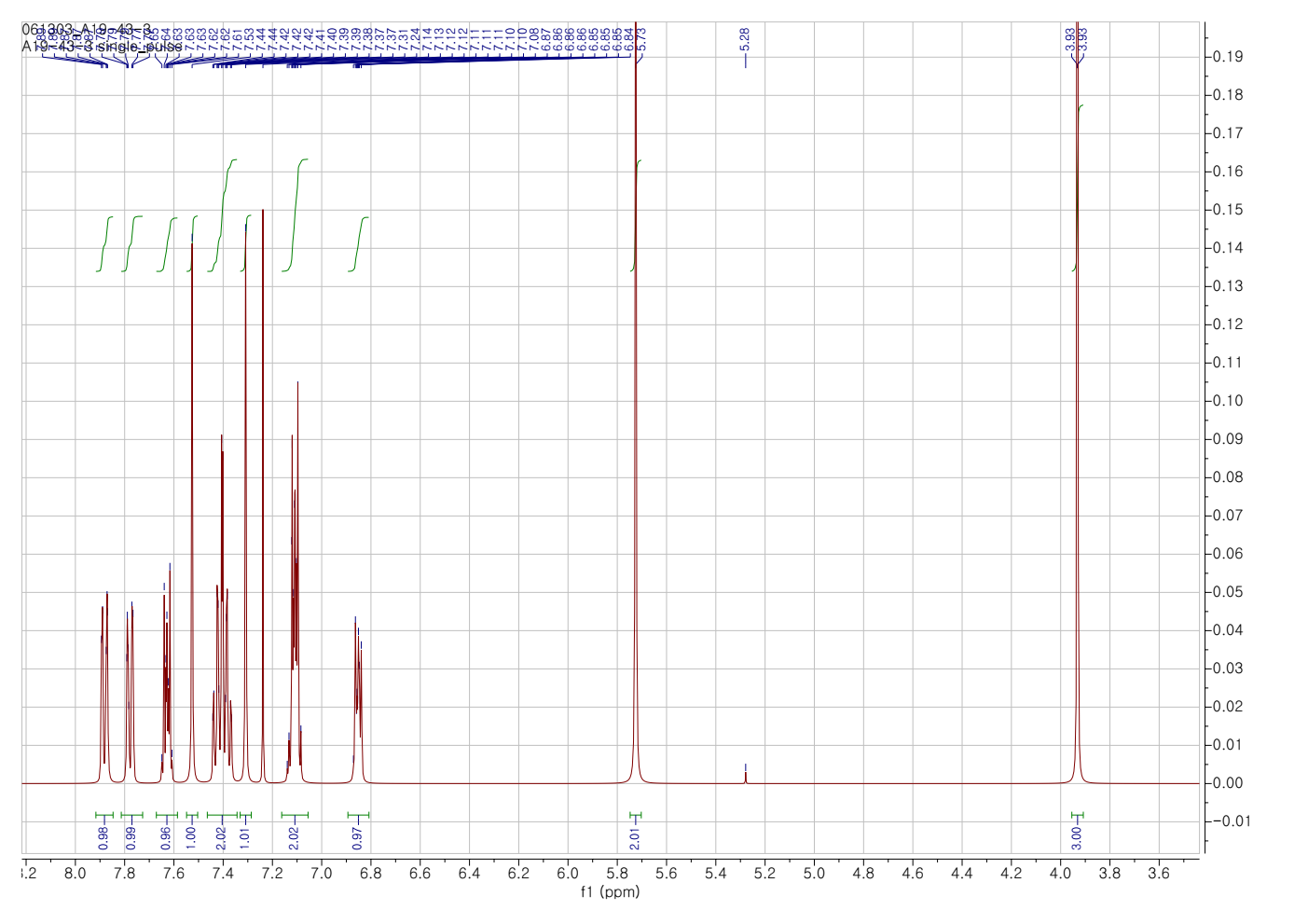

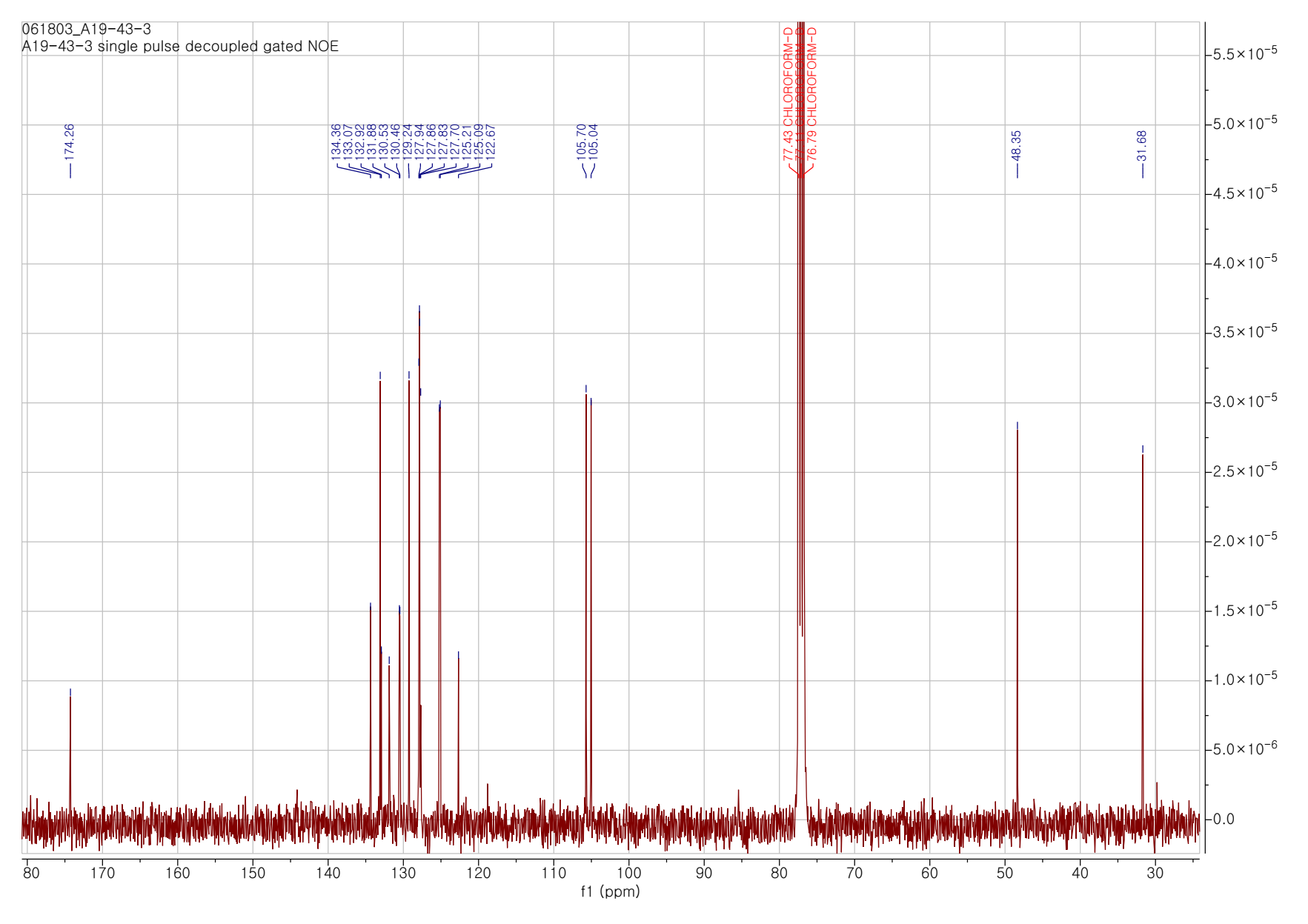


Figure S23. ^1^H-NMR and ^13^C-NMR spectra of **BSM** in CHCl_3_



| a) Mass spectra of **BSB**  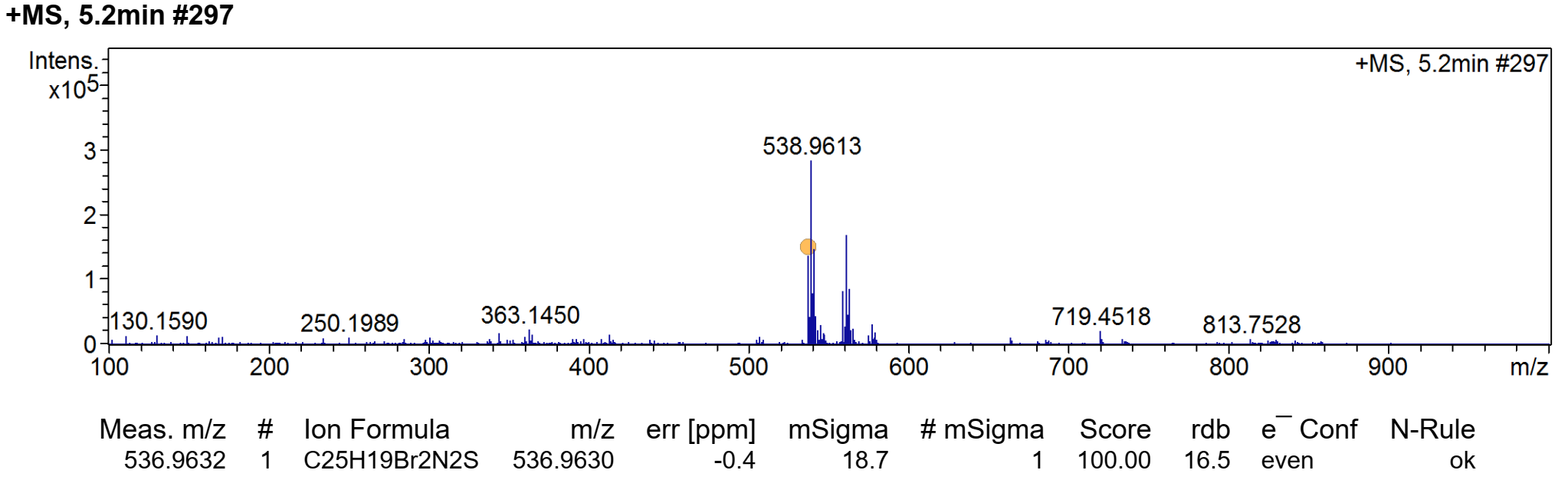 | b) Mass spectra of **BSM**  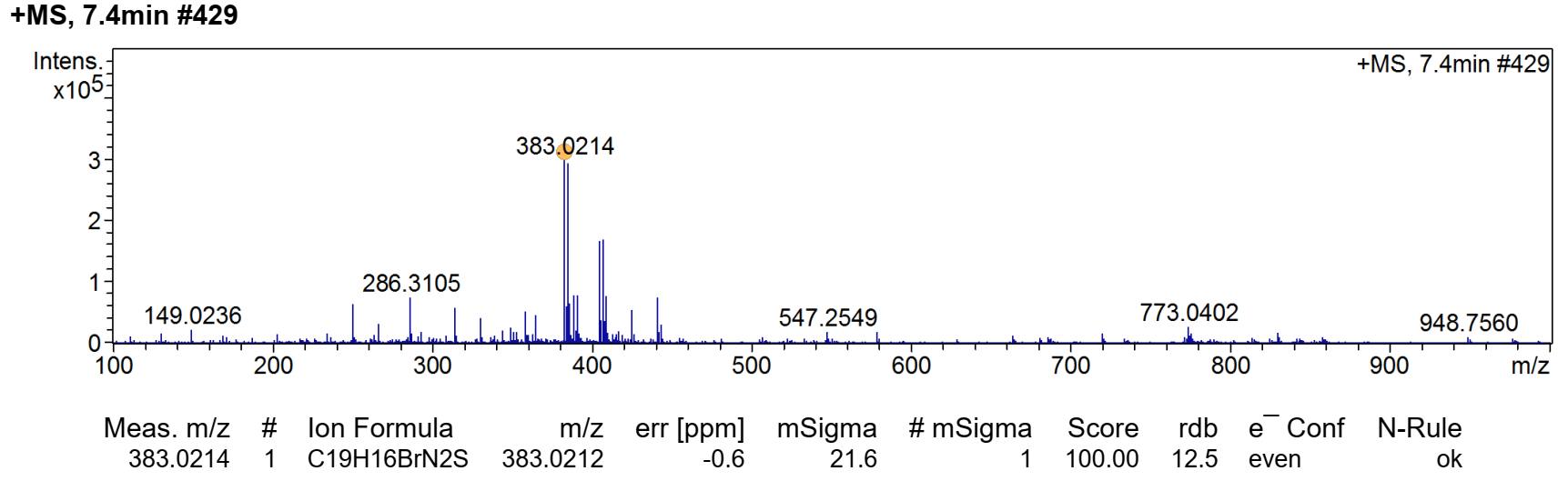 |
| --- | --- |

Figure S24. Mass spectra of (a) **BSB** and (b) **BSM**.

| a) Mass spectra of **CSB**  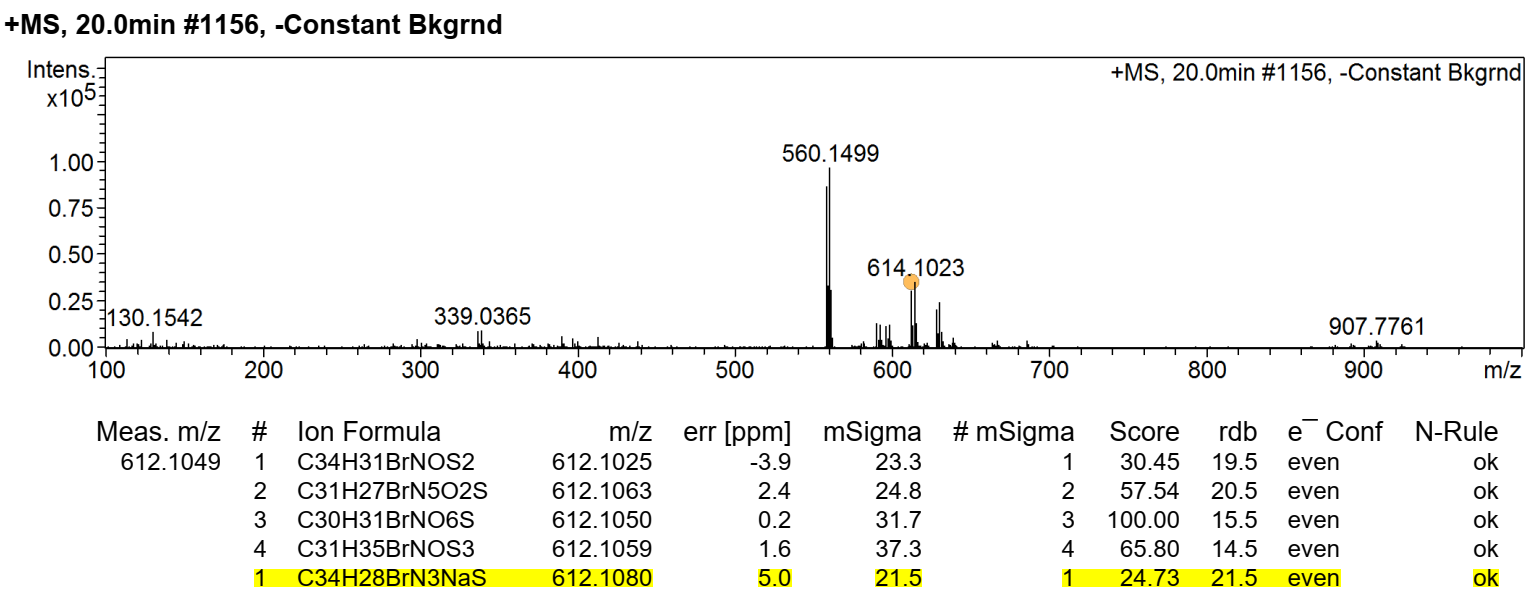 | b) Mass spectra of **CSC**  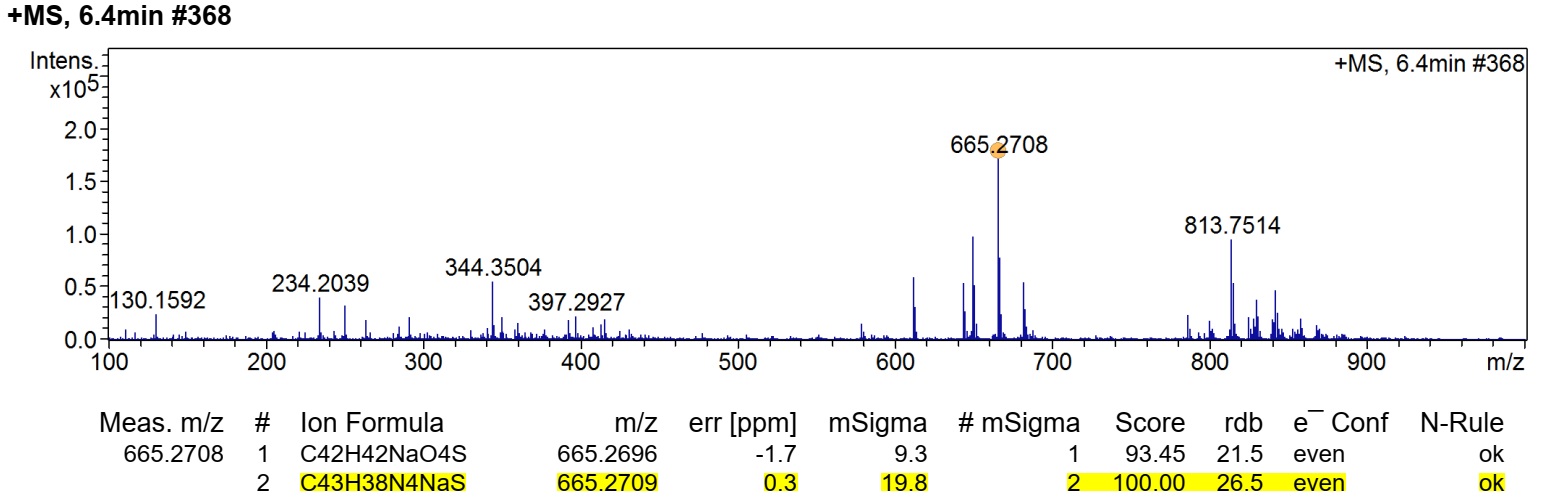 |
| --- | --- |

Figure S25. Mass spectra of (a) **CSB** and (b) **CSC**.


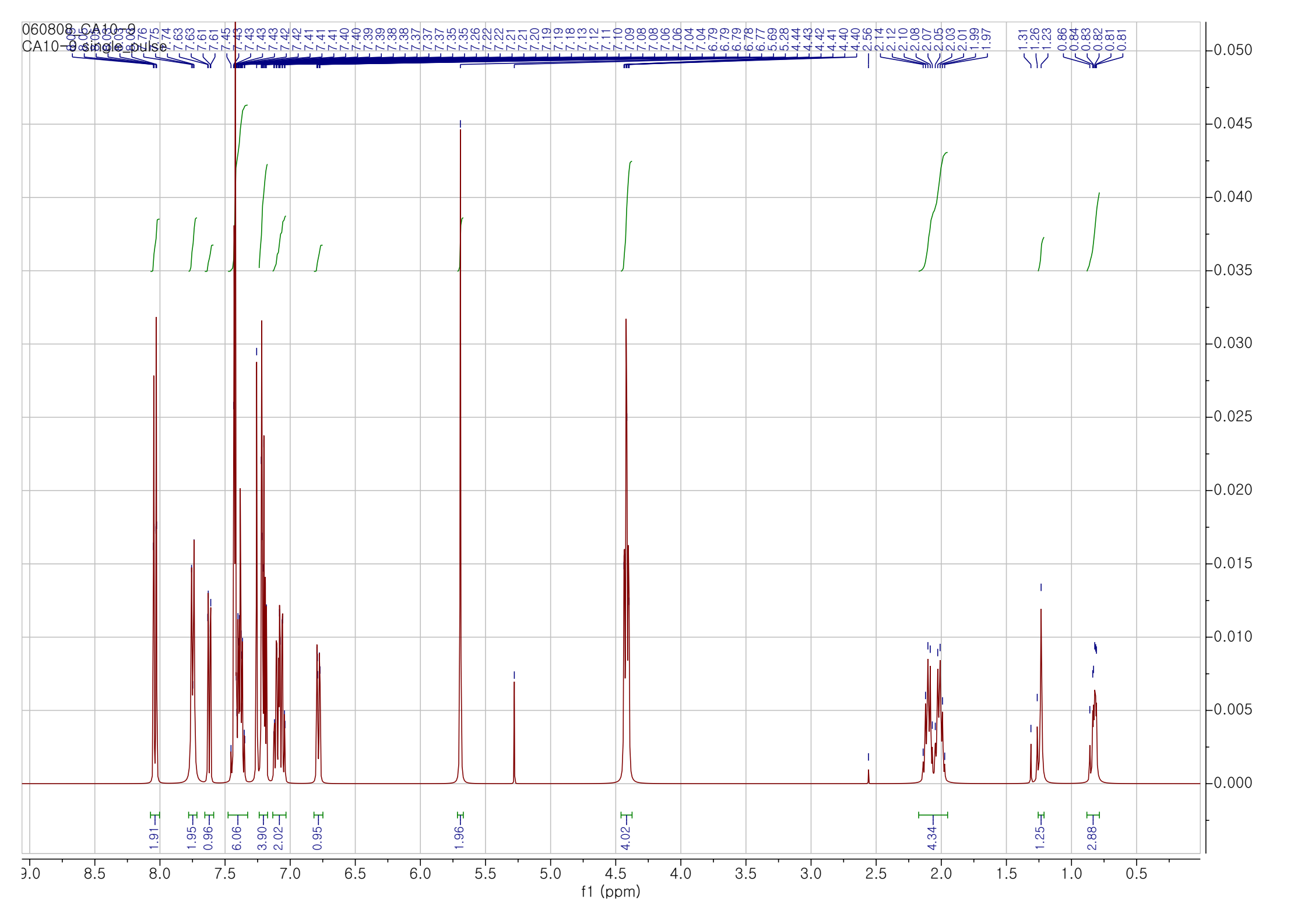

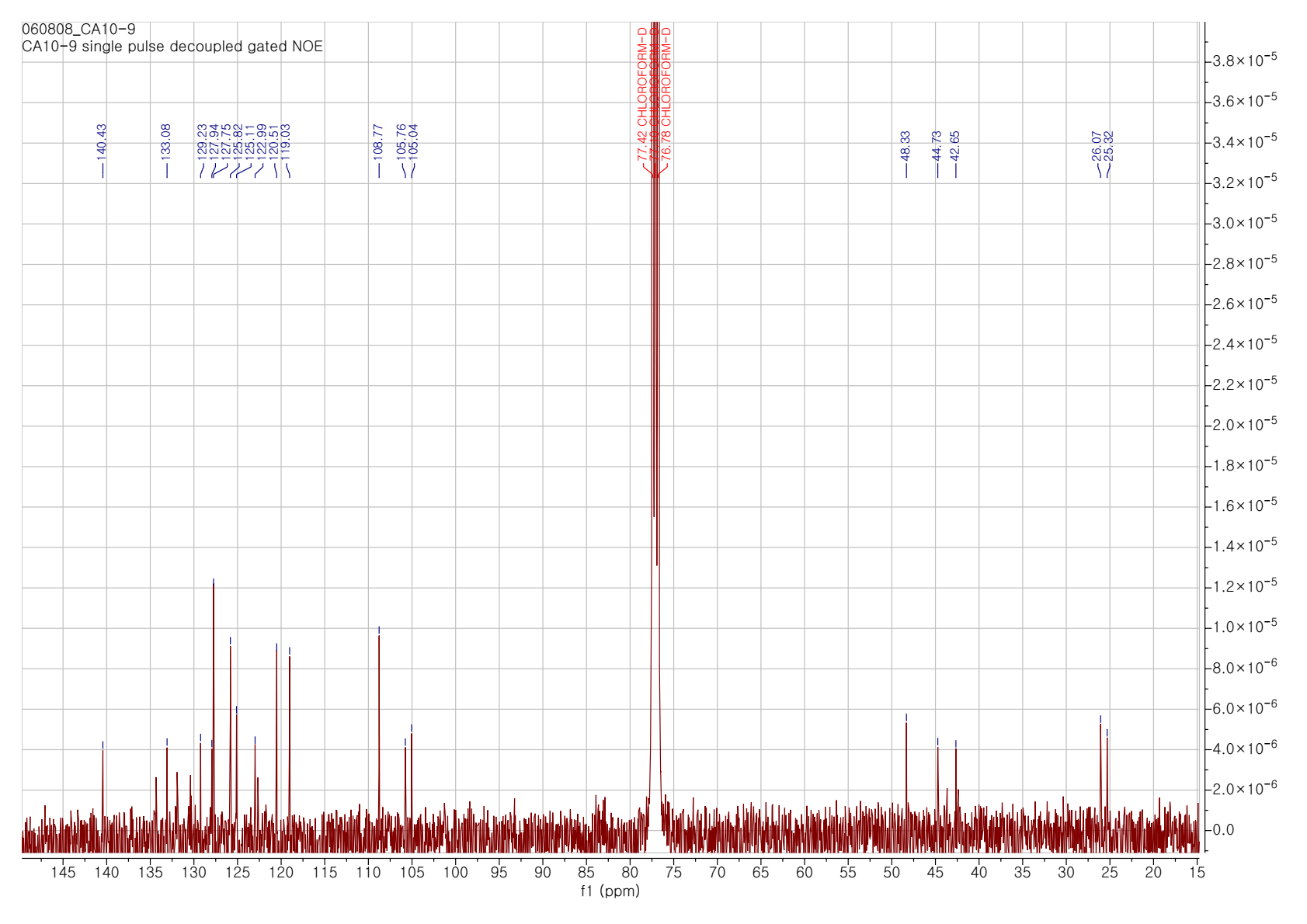


Figure S26. ^1^H-NMR and ^13^C-NMR spectra of **CSB** in CHCl_3_



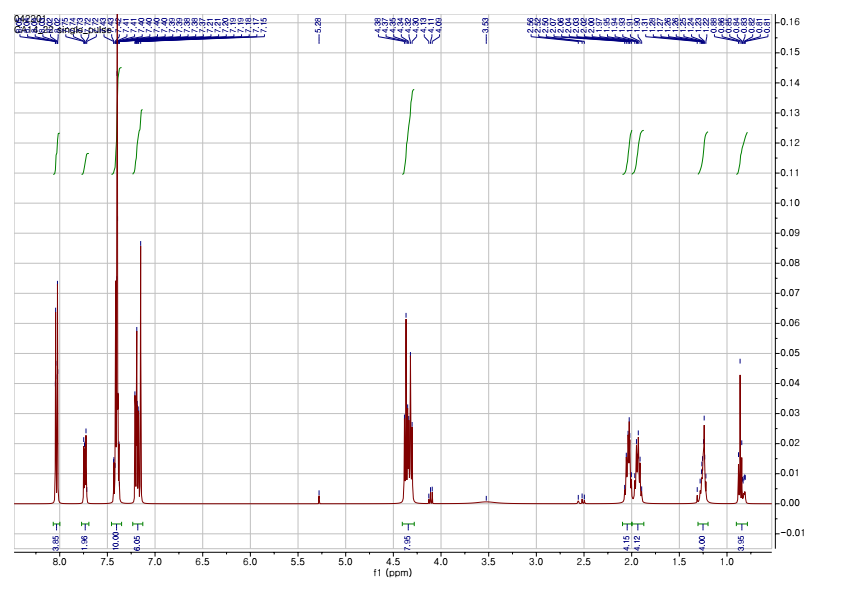

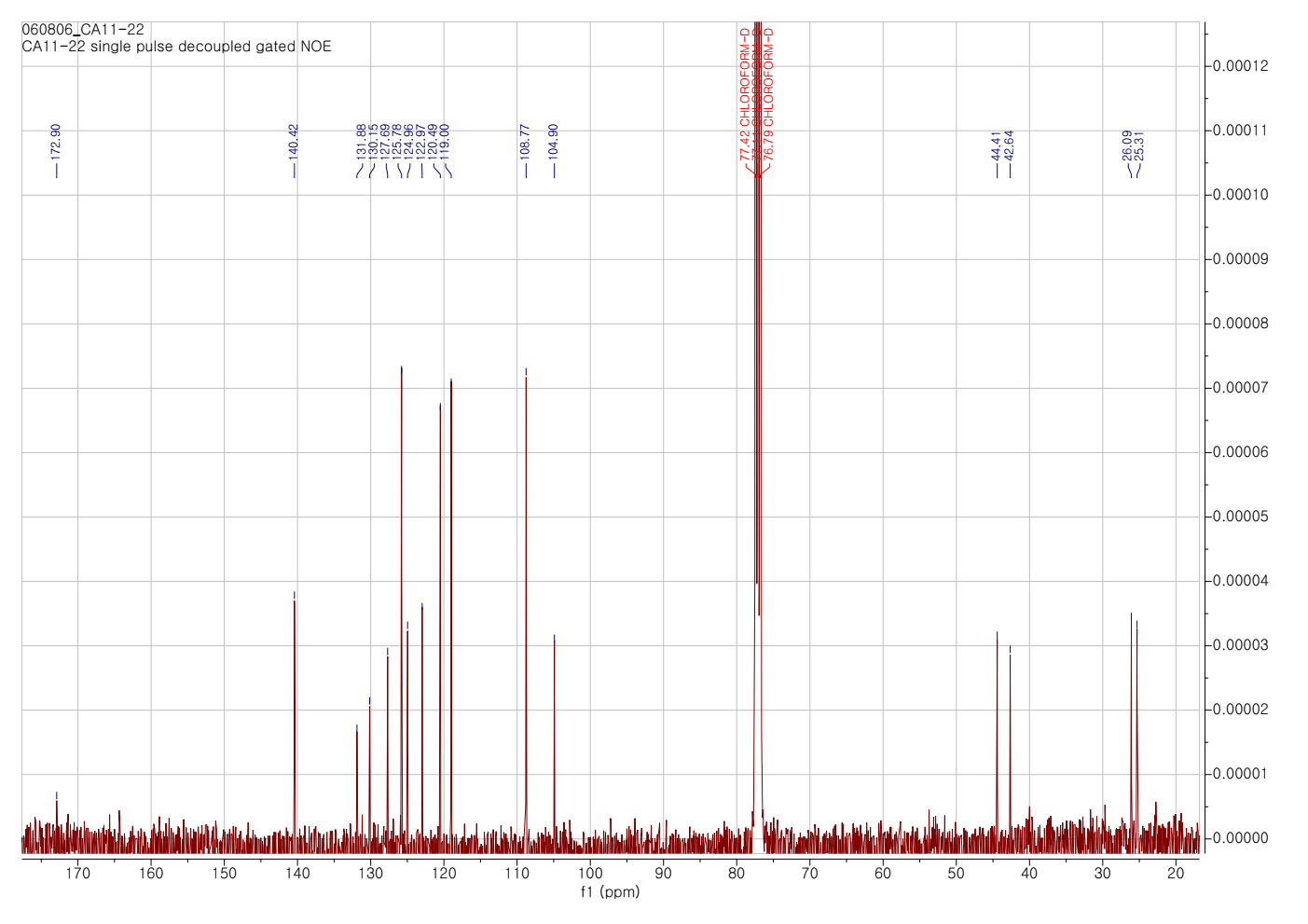


Figure S27. ^1^H-NMR and ^13^C-NMR spectra of **CSC** in CHCl_3_



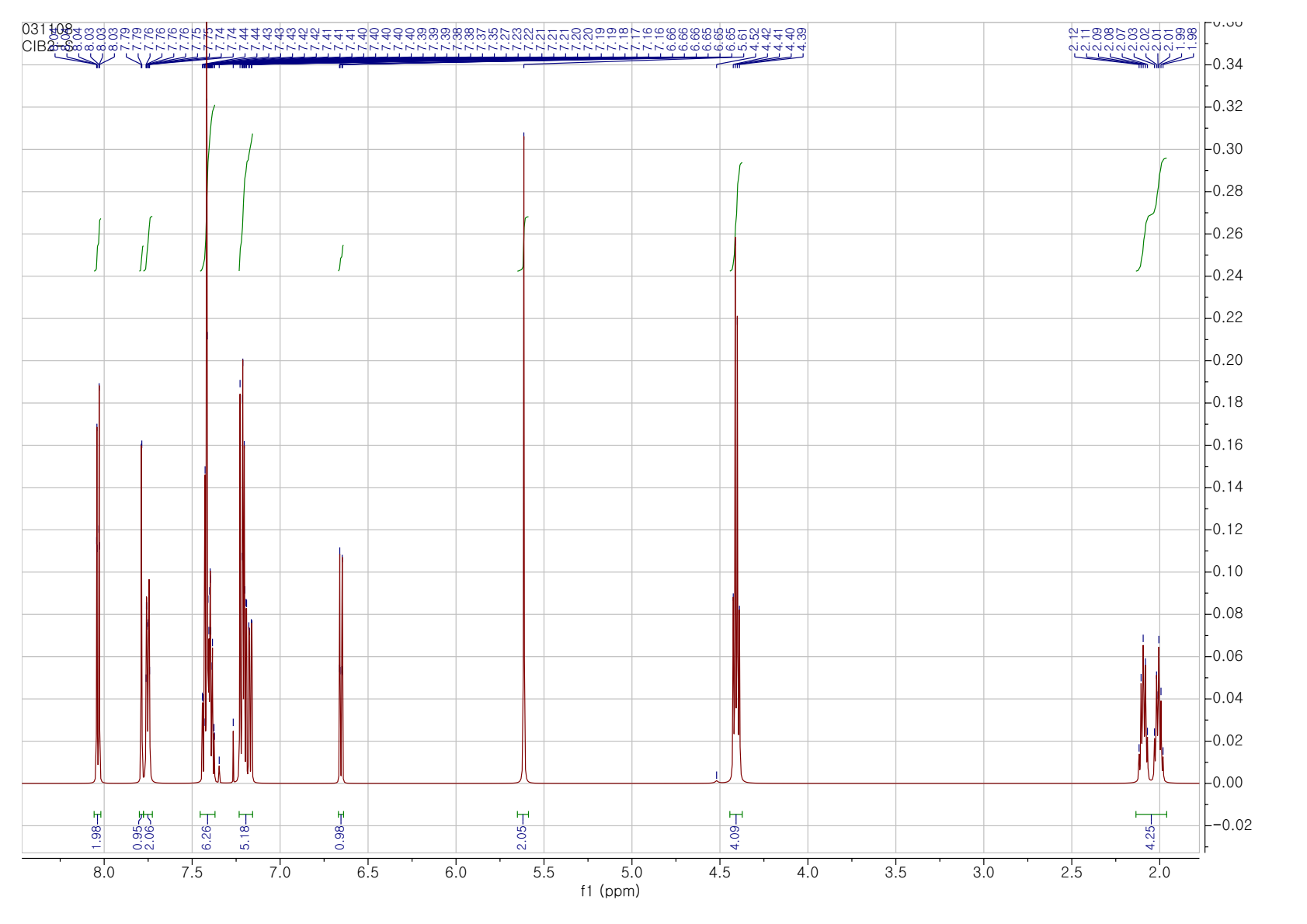

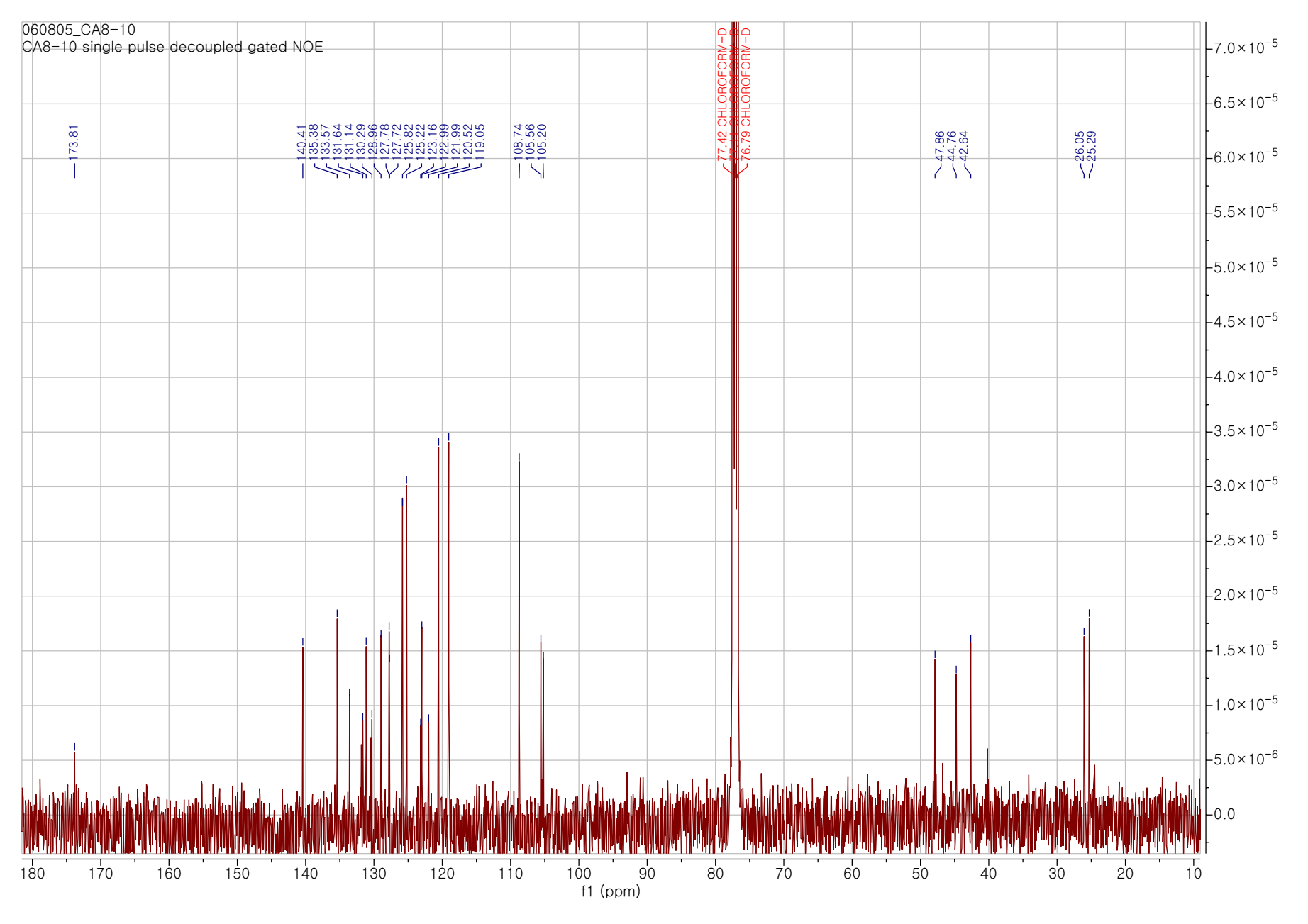


Figure S28. ^1^H-NMR and ^13^C-NMR spectra of **CSD** in CHCl_3_



| a) Mass spectra of **CSD**  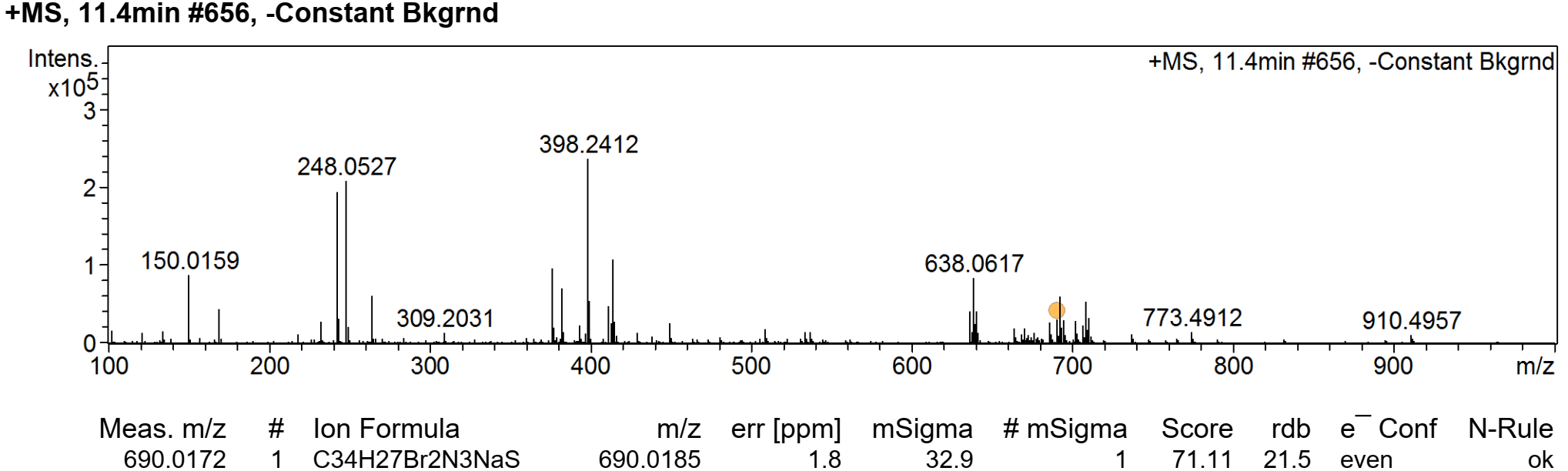 | b) Mass spectra of **CSM**  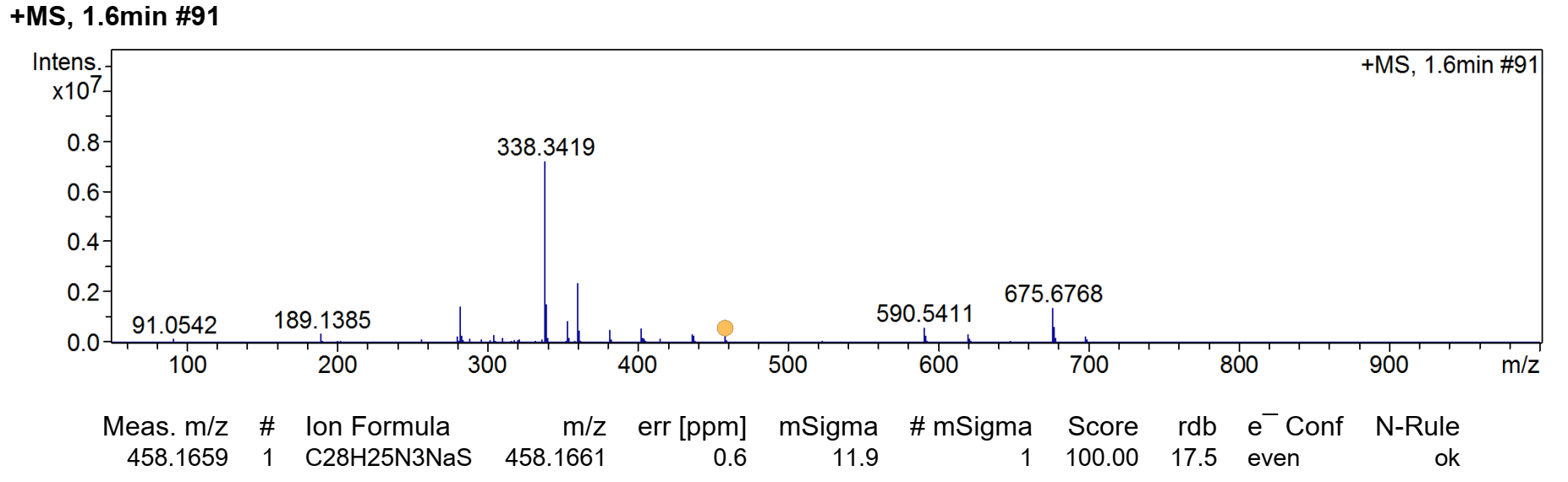 |
| --- | --- |

Figure S29. Mass spectra of (a) **CSD** and (b) **CSM**.


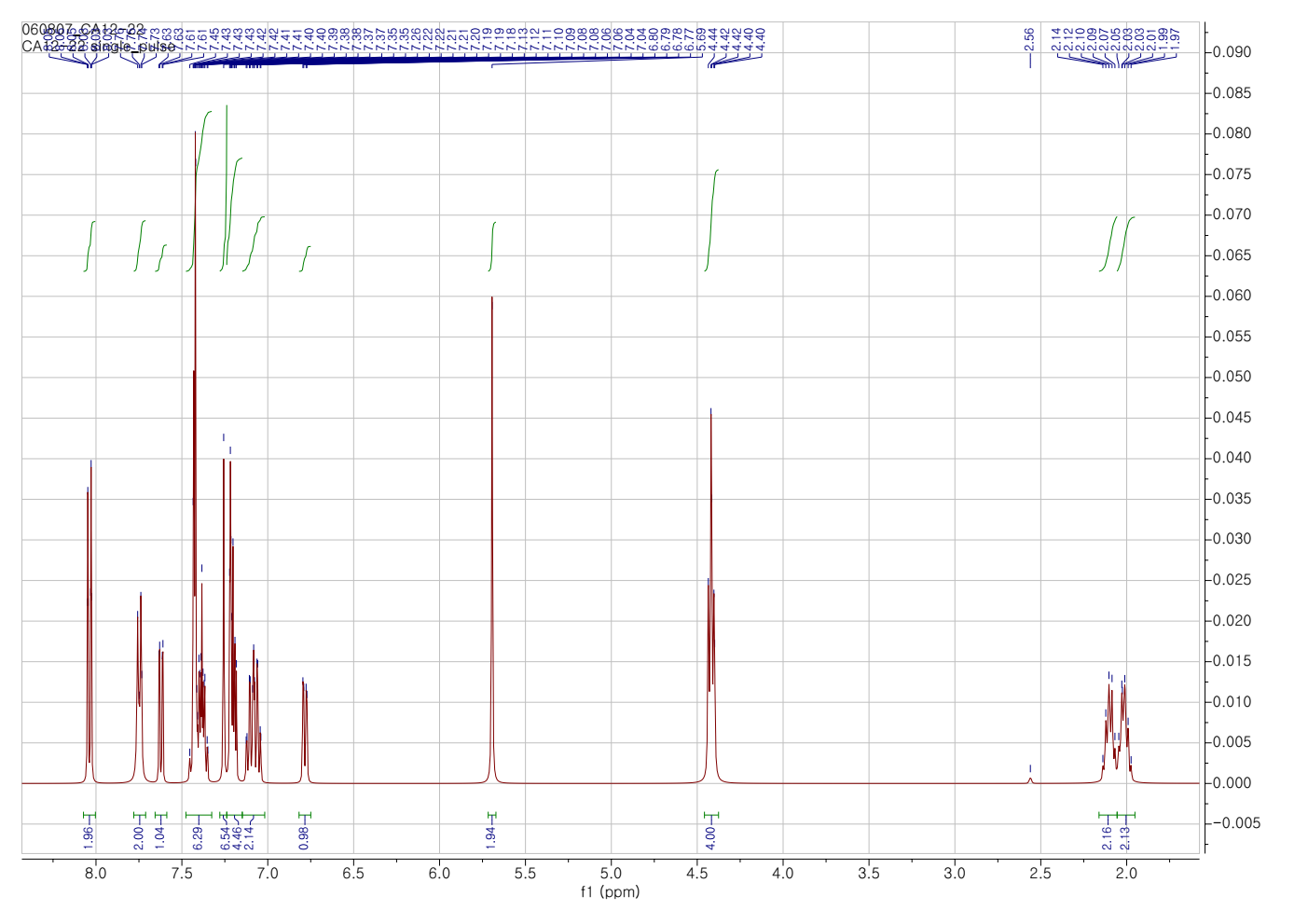

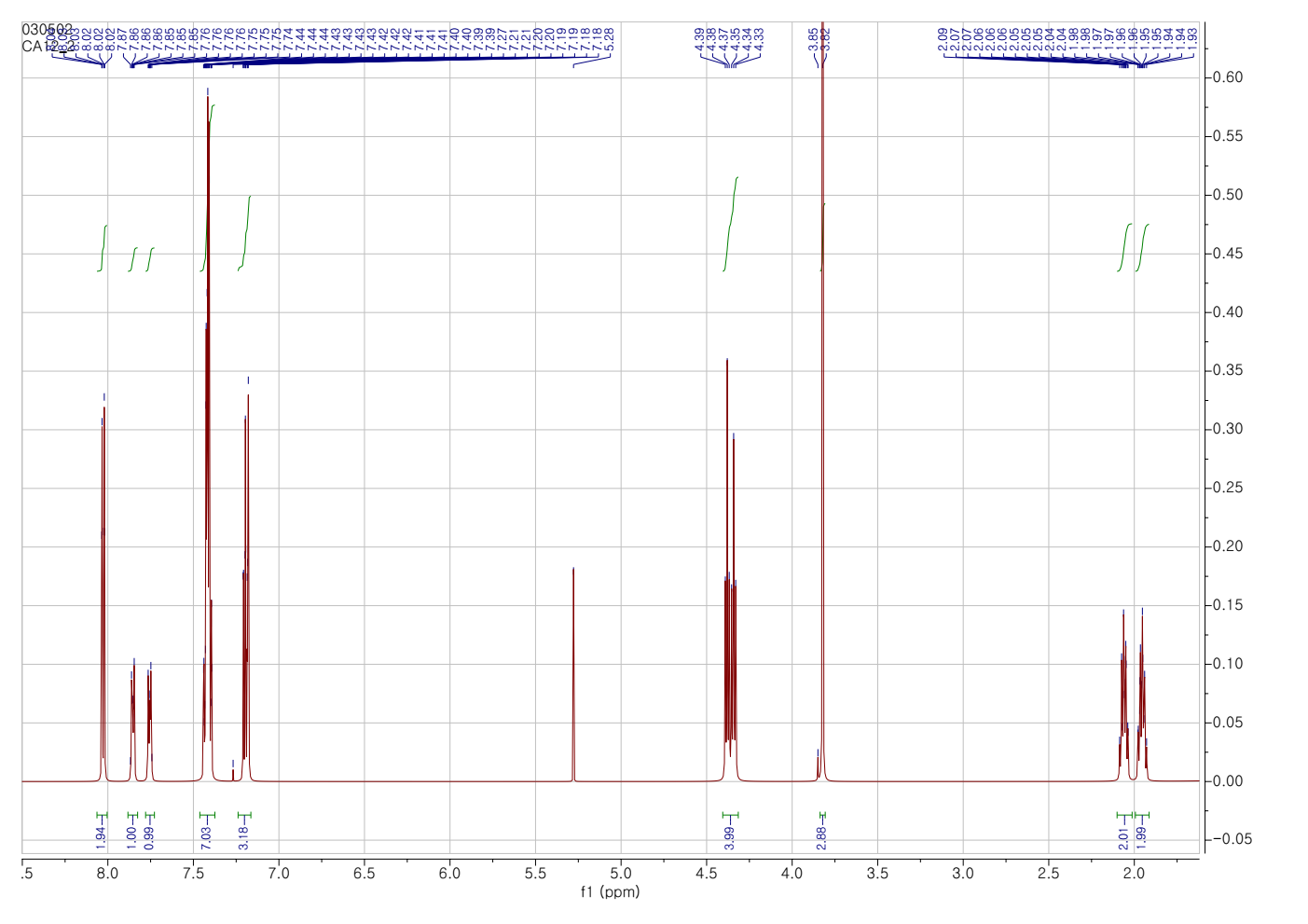


Figure S30. ^1^H-NMR and ^13^C-NMR spectra of **CSM** in CHCl_3_



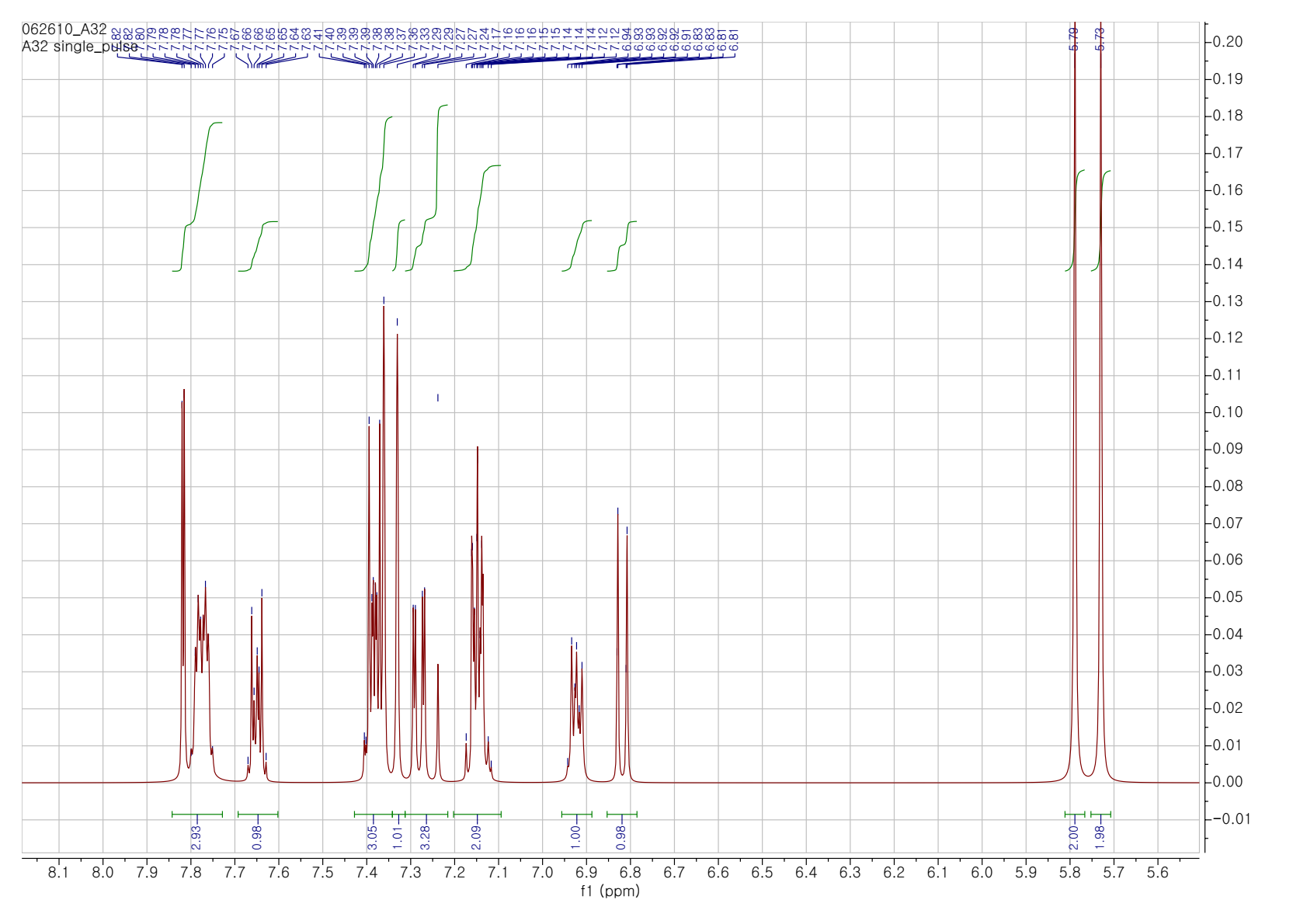

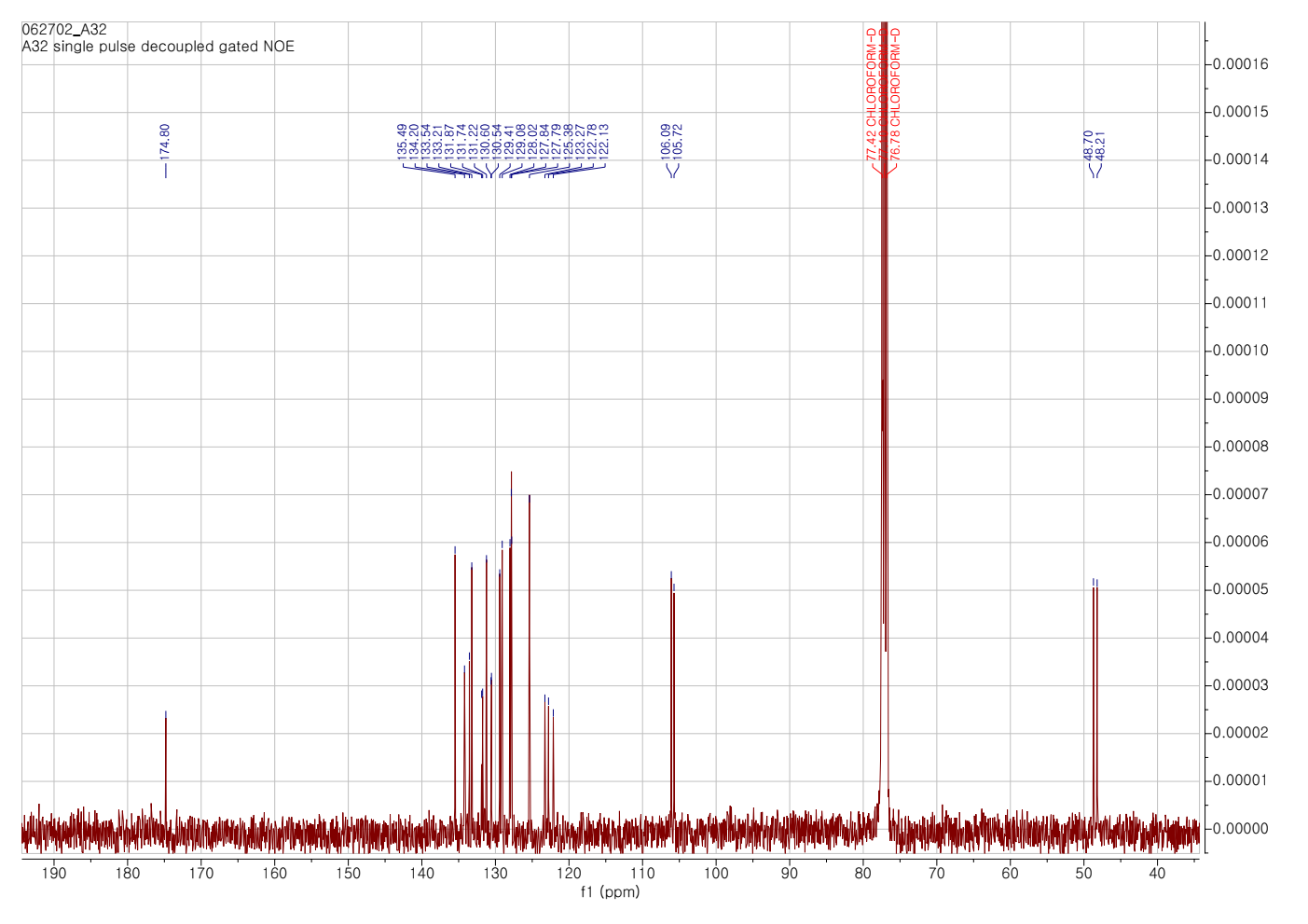


Figure S31. ^1^H-NMR and ^13^C-NMR spectra of **DSB** in CHCl_3_



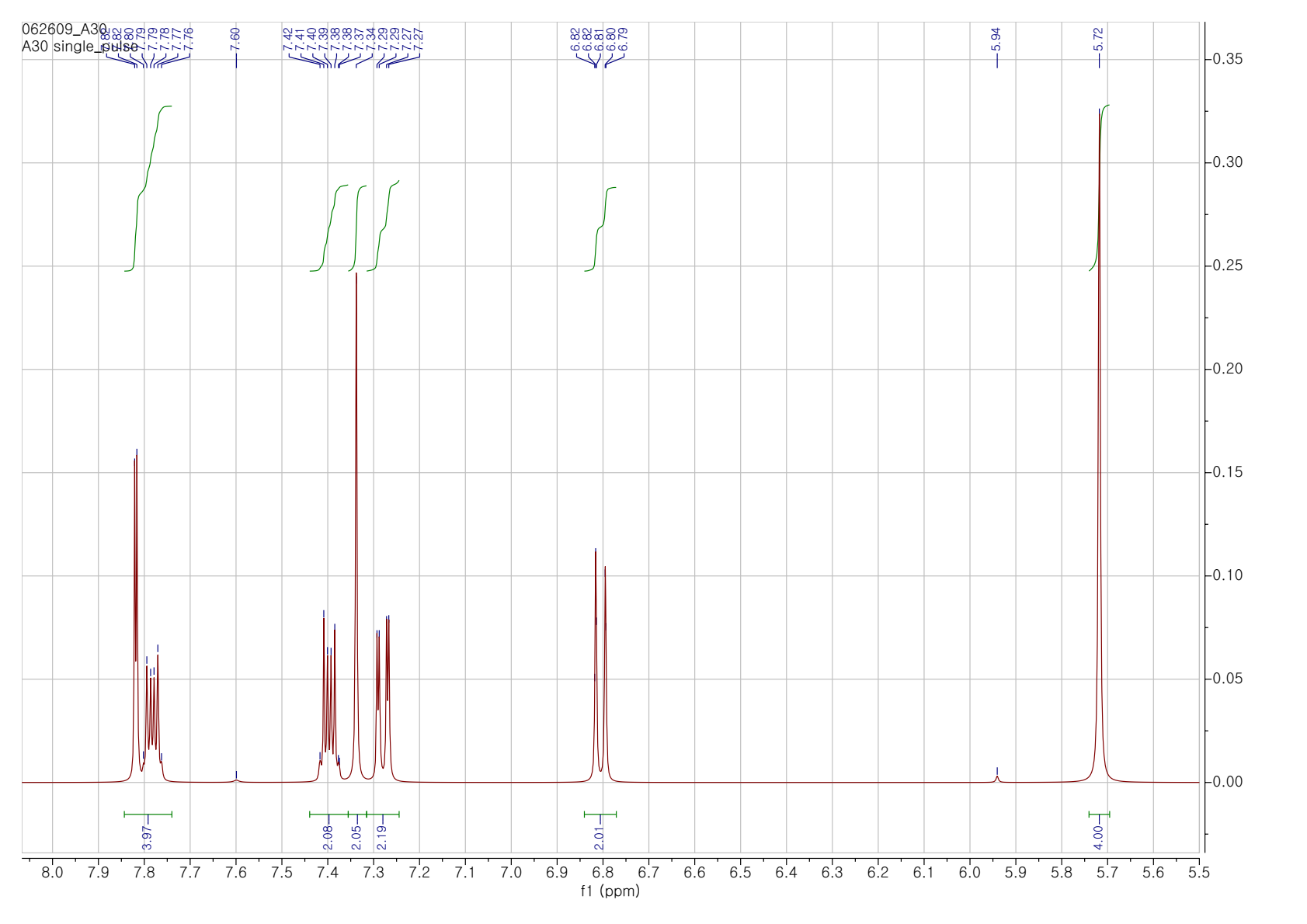

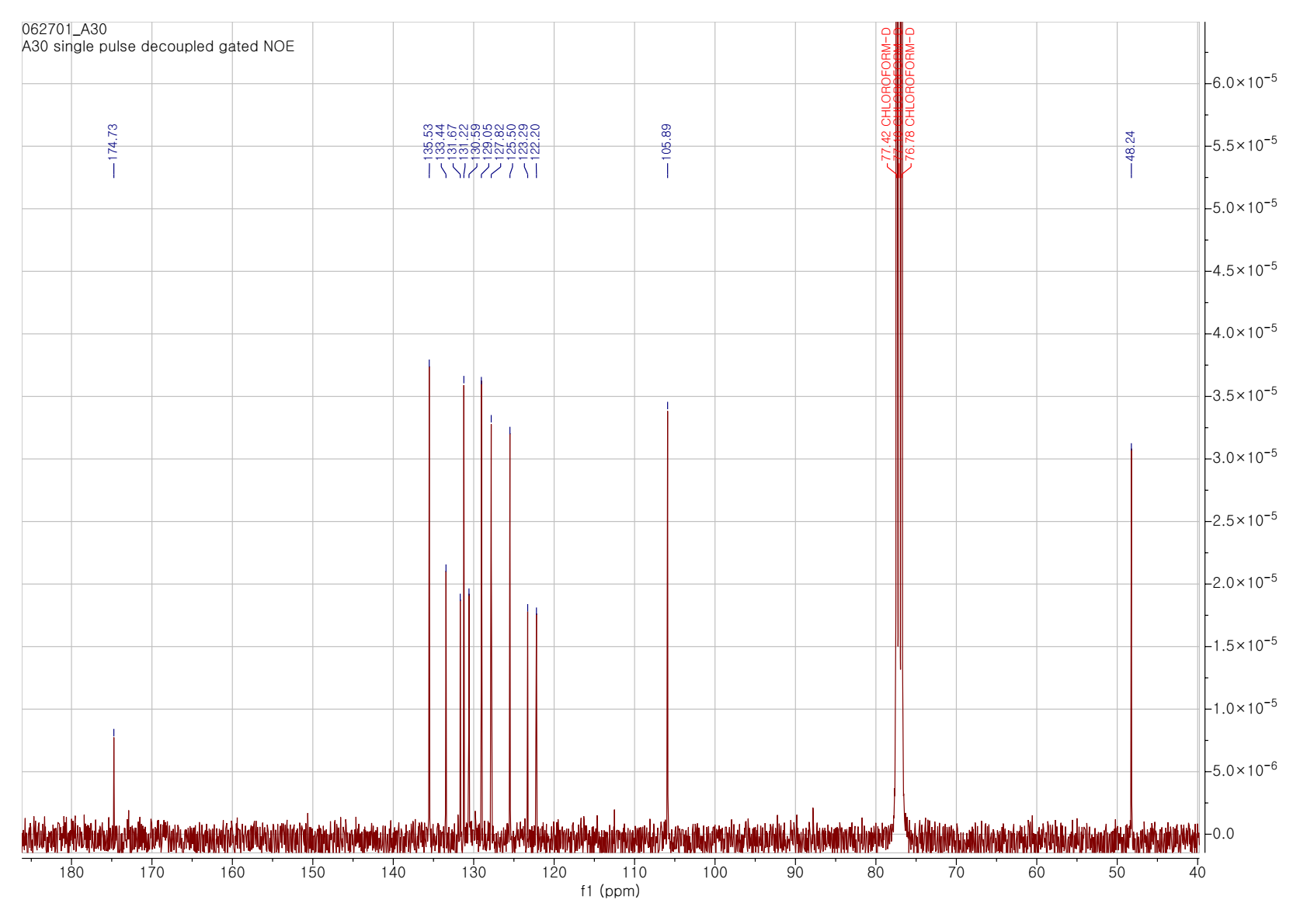


Figure S32. ^1^H-NMR and ^13^C-NMR spectra of **DSD** in CHCl_3_



| a) Mass spectra of **DSB** | b) Mass spectra of **DSD** |
| --- | --- |

Figure S33. Mass spectra of (a) **DSB** and (b) **DSD**.

Figure S34. ^1^H-NMR and ^13^C-NMR spectra of **DSM** in CHCl_3_

Figure S35. ^1^H-NMR and ^13^C-NMR spectra of **DSM’ (DIM)** in DMSO

| a) Mass spectra of **DSM** | b) Mass spectra of **DSM’ (DIM)** |
| --- | --- |

Figure S36. Mass spectra of (a) **DSM** and (b) **DSM’ (DIM)**.

- 1. **Crystallization data**

Table S1. Crystal data of **CIM** and **CIC**.

|  | **CIM** | **CIC** |
| --- | --- | --- |
| Empirical formula | C29 H30 I N3 O | C45 H47 Br N4 O2 |
| Formula weight | 563.46 | 755.77 |
| Temperature | 173(2) K | 171(2) K |
| Wavelength | 0.700 Å | 0.650 Å |
| Crystal system | Monoclinic | Triclinic |
| Space group | P2**_1_** | P-1 |
| Unit cell dimensions | a = 10.308(2) Å α= 90°.  b = 7.8540(16) Å β= 102.96(3)°.  c = 16.040(3) Å γ = 90°. | a = 8.4980(17) Å α= 84.53(3)°.  b = 10.255(2) Å β= 86.87(3)°.  c = 23.313(5) Å γ = 72.93(3)°. |
| Volume | 1265.5(5) Å3 | 1932.5(7) Å3 |
| Z | 2 | 2 |
| Density (calculated) | 1.479 Mg/m3 | 1.299 Mg/m3 |
| Absorption coefficient | 1.225 mm-1 | 0.880 mm-1 |
| F(000) | 572 | 792 |
| Crystal size | 0.120 x 0.053 x 0.044 mm3 | 0.120 x 0.080 x 0.070 mm3 |
| Theta range for data collection | 2.118 to 27.996°. | 1.606 to 30.000°. |
| Index ranges | -13<=h<=13, -10<=k<=10, -21<=l<=21 | -13<=h<=13, -15<=k<=15, -35<=l<=35 |
| Reflections collected | 11493 | 29303 |
| Independent reflections | 6350 [R(int) = 0.0633] | 14668 [R(int) = 0.0192] |
| Completeness to theta = | (24.835°) 99.3 % | (22.955°**)** 99.8 % |
| Absorption correction | Empirical | Empirical |
| Max. and min. transmission | 1.000 and 0.882 | 1.000 and 0.934 |
| Refinement method | Full-matrix least-squares on F2 | Full-matrix least-squares on F2 |
| Data / restraints / parameters | 6350 / 1 / 310 | 14668 / 0 / 474 |
| Goodness-of-fit on F2 | 1.096 | 1.151 |
| Final R indices [I>2sigma(I)] | R1 = 0.0394, wR2 = 0.1146 | R1 = 0.0624, wR2 = 0.1884 |
| R indices (all data) | R1 = 0.0410, wR2 = 0.1156 | R1 = 0.0760, wR2 = 0.1963 |
| Absolute structure parameter | -0.006(8) |  |
| Extinction coefficient | n/a | n/a |
| Largest diff. peak and hole | 1.533 and -0.765 e.Å-3 | 2.352 and -1.296 e.Å-3 |
| CCDC deposition number | 2084935 | 2084931 |

| a) | b) |
| --- | --- |

Figure S37. Crystal structure of (a) CIM and (b) CIC

| c) | d) |
| --- | --- |

Figure S38. Crystal structure of (a) CSM and (b) CSC

Table S2. Crystal data of **CSM** and **CSC**.

|  | **CSM** | **CSC** |
| --- | --- | --- |
| Empirical formula | C28 H25 N3 S | _C43 H38 N4 S_ |
| Formula weight | 435.57 | 642.83 |
| Temperature | 173(2) K | 173(2) K |
| Wavelength | 0.650 Å | 0.800 Å |
| Crystal system | Monoclinic | Triclinic |
| Space group | P2**_1_**/n | P-1 |
| Unit cell dimensions | a = 9.6770(19) Å α= 90°  b = 14.016(3) Å β= 90.67(3)°  c = 16.317(3) Å γ = 90° | a = 9.3930(19) Å α= 89.06(3)°.  b = 15.420(3) Å β= 86.63(3)°.  c = 23.453(5) Å γ = 84.27(3)°. |
| Volume | 2213.0(8) Å3 | 3373.9(12) Å3 |
| Z | 4 | 4 |
| Density (calculated) | 1.307 Mg/m3 | 1.266 Mg/m3 |
| Absorption coefficient | 0.133 mm-1 | 0.177 mm-1 |
| F(000) | 920 | 1360 |
| Crystal size | 0.300 x 0.120 x 0.100 mm3 | 0.110 x 0.020 x 0.019 mm3 |
| Theta range for data collection | 2.226 to 25.998°. | 1.494 to 28.000°. |
| Index ranges | -13<=h<=13, -18<=k<=18, -21<=l<=22 | -11<=h<=11, -18<=k<=18, -27<=l<=27 |
| Reflections collected | 18790 | 22674 |
| Independent reflections | 5588 [R(int) = 0.1117] | 11358 [R(int) = 0.0400] |
| Completeness to theta | 22.955° - 98.0 % | 28.000° - 99.6 % |
| Absorption correction | Empirical | Empirical |
| Max. and min. transmission | 1.000 and 0.923 | 1.000 and 0.859 |
| Refinement method | Full-matrix least-squares on F2 | Full-matrix least-squares on F2 |
| Data / restraints / parameters | 5588 / 0 / 290 | 11358 / 825 / 1009 |
| Goodness-of-fit on F2 | 1.065 | 1.724 |
| Final R indices [I>2sigma(I)] | R1 = 0.0776, wR2 = 0.2205 | R1 = 0.1376, wR2 = 0.4293 |
| R indices (all data) | R1 = 0.0835, wR2 = 0.2291 | R1 = 0.1672, wR2 = 0.4424 |
| Extinction coefficient | n/a | 0.017(4) |
| Largest diff. peak and hole | 1.023 and -0.422 e.Å-3 | 0.549 and -0.817 e.Å-3 |
| CCDC deposition number | 2084934 | 2084933 |

- 1. **Photophysical results**

| a) | b) |
| --- | --- |

Figure S39. UV-Vis (dot line) and Fluorescence emission (dash dot line) spectra of (a) **BIB** (80 µM, λ_ex_ = 325 nm) and (b) **BSB** (20 µM, λ_ex_ = 350 nm) in several solvent.

| a) | b) |
| --- | --- |

Figure S40. UV-Vis (dot line) and Fluorescence emission (dash dot line) spectra of (a) **BIM** (80 µM, λ_ex_ = 325 nm) and (b) **BSM** (10 µM, λ_ex_ = 350 nm) in several solvent.

| a) | b) |
| --- | --- |

Figure S41. UV-Vis (dot line) and Fluorescence emission (dash dot line) spectra of (a) **CIB** (80 µM, λ_ex_ = 325 nm) and (b) **CSB** (10 µM, λ_ex_ = 350 nm) in several solvent.

| a) | b) |
| --- | --- |

Figure S42. UV-Vis (dot line) and Fluorescence emission (dash dot line) spectra of (a) **CIC** (80 µM, λ_ex_ = 325 nm) and (b) **CSC** (10 µM, λ_ex_ = 350 nm) in several solvent.

| a) | b) |
| --- | --- |

Figure S43. UV-Vis (dot line) and Fluorescence emission (dash dot line) spectra of (a) **CID** (80 µM, λ_ex_ = 325 nm) and (b) **CSD** (20 µM, λ_ex_ = 350 nm) in several solvent.

| a) | b) |
| --- | --- |

Figure S44. UV-Vis (dot line) and Fluorescence emission (dash dot line) spectra of (a) **CIM** (80 µM, λ_ex_ = 325 nm) and (b) **CSM** (20 µM, λ_ex_ = 350 nm) in several solvent.

| a) | b) |
| --- | --- |

Figure S45. UV-Vis (dot line) and Fluorescence emission (dash dot line) spectra of (a) **DIB** (80 µM, λ_ex_ = 325 nm) and (b) **DSB** (10 µM, λ_ex_ = 350 nm) in several solvent.

| a) | b) |
| --- | --- |

Figure S46. UV-Vis (dot line) and Fluorescence emission (dash dot line) spectra of (a) **DID** (80 µM, λ_ex_ = 325 nm) and (b) **DSD** (20 µM, λ_ex_ = 350 nm) in several solvent.

| a) | b) |
| --- | --- |

Figure S47. UV-Vis (dot line) and Fluorescence emission (dash dot line) spectra of (a) **DIM** (80 µM, λ_ex_ = 325 nm) and (b) **DSM** (10 µM, λ_ex_ = 350 nm) in several solvent.

| a) | b) |
| --- | --- |

Figure S48. (a) The fluorescence emission spectra of **DIM** (5 µM) in DMF/pH 7.4 PBS buffer (0 - 99.5%); (b) the emission under UV-365 nm irradiation **DIM** (5 µM) in DMF/pH 7.4 PBS buffer and DMF/Tol (0 - 99.5%).

| a) | b) |
| --- | --- |
| c) | d) |

Figure S49. The fluorescence emission spectra of **DID** (5 µM) in (a) DMF/pH 7.4 PBS buffer and (b) DMF/Tol (0 - 99.5%); (c) the fluorescence emission intensity and (d) the emission under UV-365 nm irradiation of **DID** (5 µM) in DMF/ pH 7.4 PBS buffer and DMF/Tol (0 - 99.5%).

| a) | b) |
| --- | --- |

Figure S50. (a) Fluorescence emission spectra (λ_ex_ = 325 nm; slit 5/5) of **DSD** (5 µM) upon the treatment of ClO^-^ (0 - 80 µM) in pH 7.4 PBS buffer (0.05 % DMF); (b) Plot of fluorescence intensity of **DSM** (5 µM) upon the treatment of ClO^-^ (0 - 55 µM) in pH 7.4 PBS buffer (0.05 % DMF).

| a) | b) |
| --- | --- |

Figure S51. (a) UV-vis absorbance and (b) fluorescence emission (λ_ex_ = 325 nm, slit 5/5) spectra of **DSM** (5 µM) upon the treatment of ClO^-^ (50 µM); ROO^•^ (1 mM); NO^•^ (1 mM), H_2_O_2_ (1 mM), TBHP (1 mM), ONOO^-^ (200 µM) and ^•^OH (200 µM) in pH 7.4 PBS buffer (0.05 % DMF).

- 1. **Computational calculation results**

Table S1. Molecular orbital and energies of **BIB** and **BSB**, **BIM** and **BSM**, **CIB** and **CSB**.

|  | **BIB** | **BSB** | **BIM** | **BSM** |
| --- | --- | --- | --- | --- |
| LUMO+2 | -3.88 eV | -0.90 eV | -3.83 eV | -0.83 eV |
| LUMO+1 | -3.91 eV | -0.94 eV | -4.04 eV | -0.89 eV |
| LUMO | -5.09 eV | -1.75 eV | -5.34 eV | -1.72 eV |
| HOMO | -8.95 eV | -5.87 eV | -9.13 eV | -5.80 eV |
| HOMO-1 | -9.75 eV | -6.05 eV | -9.90 eV | -6.02 eV |
| HOMO-2 | -9.78 eV | -6.20 eV | -10.04 eV | -6.14 eV |

Table S2. Molecular orbital and energies of **CIB** and **CSB**, **CID** and C**SD.**

|  | **CIB** | **CSB** | **CID** | **CSD** |
| --- | --- | --- | --- | --- |
| LUMO+2 | -3.82 eV | -0.93 eV | -4.03 eV | -1.15 eV |
| LUMO+1 | -4.03 eV | 1.11 eV | -4.20 eV | -1.23 eV |
| LUMO | -5.29 eV | -1.79 eV | -5.35 eV | -1.86 eV |
| HOMO | -7.30 eV | -5.63 eV | -7.31 eV | -5.65 eV |
| HOMO-1 | -7.59 eV | -5.88 eV | -7.60 eV | -5.96 eV |
| HOMO-2 | -8.64 eV | -5.98 eV | -8.65 eV | -6.00 eV |

Table S3. Molecular orbital and energies of **CIC** and **CSC**

|  | **CIC** | **CSC** |
| --- | --- | --- |
| LUMO+2 | -3.72 eV | -1.12 eV |
| LUMO+1 | -3.96 eV | -1.13 eV |
| LUMO | -5.50 eV | -1.82 eV |
| HOMO | -7.35 eV | -5.63 eV |
| HOMO-1 | -7.36 eV | -5.64 eV |
| HOMO-2 | -7.64 eV | -5.89 eV |

Table S4. Molecular orbital and energies of **CIM** and **CSM**, **DIB** and **DSB**.

|  | **CIM** | **CSM** | **DIB** | **DSB** |
| --- | --- | --- | --- | --- |
| LUMO+2 | -3.79 eV | -0.58 eV | -3.95 eV | -1.15 eV |
| LUMO+1 | -4.00 eV | -1.09 eV | -4.10 eV | -1.22 eV |
| LUMO | -5.57 eV | -1.75 eV | -5.14 eV | -1.82 eV |
| HOMO | -7.37 eV | -5.61 eV | -9.00 eV | -5.95 eV |
| HOMO-1 | -7.65 eV | -5.81 eV | -9.67 eV | -6.13 eV |
| HOMO-2 | -8.70 eV | -5.97 eV | -9.80 eV | -6.29 eV |

Table S5. Molecular orbital and energies of **DID** and **DSD**, **DIM** and **DSM**.

|  | **DID** | **DSD** | **DIM** | **DSM** |
| --- | --- | --- | --- | --- |
| LUMO+2 | -4.11 eV | -1.25 eV | -4.04 eV | -1.11 eV |
| LUMO+1 | -4.15 eV | -1.28 eV | -4.22 eV | -1.19 eV |
| LUMO | -5.19 eV | -1.90 eV | -5.40 eV | -1.80 eV |
| HOMO | -9.04 eV | -6.04 eV | -9.17 eV | -5.89 eV |
| HOMO-1 | -9.69 eV | -6.21 eV | -9.77 eV | -6.10 eV |
| HOMO-2 | -9.70 eV | -6.38 eV | -10.09 eV | -6.23 eV |

Table S6. Excitation energy (eV) with significant oscillator strength (f) for the **R_1_IR_2_** molecules predicted using different DFT functionals with 6-31+G(2d,p) basis set.

|  | **BIB** | **BIM** | **CIB** | **CIC** | **CID** |
| --- | --- | --- | --- | --- | --- |
| Expt. | 3.7754 eV | 3.8267 eV | 3.8149 eV | 3.7800 eV | 3.8126 eV |
| LSDA | 3.1504 eV  f=0.0485 | 3.9721 eV  f=0.0846 | 3.1343 eV  f=0.0528 | 3.1308 eV  f=0.0611 | 3.1255 eV  f=0.0556 |
| BVP86 | 3.1723 eV  f=0.0495 | 3.9822 eV  f=0.0742 | 3.1572 eV  f=0.0547 | 3.1508 eV  f=0.0628 | 3.1454 eV  f=0.0542 |
| B3LYP | 4.1823 eV  f=0.1082 | 4.1948 eV  f=0.0982 | 4.1895 eV  f=0.0910 | 3.5262 eV  f=0.0977 | 3.5244 eV  f=0.0843 |
| **CAM-B3LYP** | **3.9624 eV**  **f=0.1178** | **3.9625 eV**  **f=0.1229** | **3.9506 eV**  **f=0.1351** | **3.9413 eV**  **f=0.1577** | **3.9443 eV**  **f=0.1357** |
| B3PW91 | 4.2083 eV  f=0.1034 | 4.2223 eV  f=0.0935 | 3.2169 eV  f=0.0870 | 3.5597 eV  f=0.0984 | 3.5556 eV  f=0.0847 |
| MPW1PW91 | 4.2762 eV  f=0.1076 | 4.2830 eV  f=0.0941 | 3.6512 eV  f=0.0922 | 3.6449 eV  f=0.1054 | 4.2758 eV  f=0.0935 |
| PBEPBE | 3.1751 eV  f=0.0485 | 3.9796 eV  f=0.0762 | 3.1570 eV  f=0.0541 | 3.1477 eV  f=0.0627 | 3.1458 eV  f=0.0551 |
| HSEH1PBE | 4.2404 eV  f=0.1012 | 4.2560 eV  f=0.0944 | 3.5955 eV  f=0.0873 | 3.5895 eV  f=0.1021 | 3.5855 eV  f=0.0870 |
| HCTH | 3.9148 eV  f=0.0495 | 4.0018 eV  f=0.0527 | 3.1738 eV  f=0.0553 | 3.1553 eV  f=0.0615 | 3.1797 eV  f=0.0696 |
| TPSSTPSS | 4.0006 eV  f=0.0583 | 4.0127 eV  f=0.0546 | 3.2565 eV  f=0.0628 | 3.2418 eV  f=0.0677 | 3.2409 eV  f=0.0628 |
| 𝞈B97XD | 3.9935 eV  f=0.1211 | 3.9985 eV  f=0.1262 | 3.9872 eV  f=0.1391 | 3.9827 eV  f=0.1619 | 3.9817 eV  f=0.1397 |
| APFD | 4.2527 eV  f=0.1077 | 4.2619 eV  f=0.0940 | 3.6181 eV  f=0.0892 | 3.6123 eV  f=0.1028 | 4.2544 eV  f=0.0929 |
| BhandHLYP | 4.0075 eV  f=0.1180 | 4.0062 eV  f=0.1230 | 3.9942 eV  f=0.1346 | 3.9836 eV  f=0.1561 | 3.9872 eV  f=0.1351 |
| LC-𝞈PBE | 4.2624 eV  f=0.1483 | 4.2636 eV  f=0.1541 | 4.2545 eV  f=0.1696 | 4.2483 eV  f=0.1974 | 4.2503 eV  f=0.1706 |
| M06-2X | 3.9941 eV  f=0.1170 | 4.0009 eV  f=0.1224 | 3.9849 eV  f=0.1363 | 3.9797 eV  f=0.1613 | 3.9781 eV  f=0.1369 |
| M06 | 4.1486 eV  f=0.1229 | 4.1562 eV  f=0.1066 | 4.1498 eV  f=0.1008 | 3.8882 eV  f=0.1005 | 4.1465 eV  f=0.1073 |
| M06L | 4.0808 eV  f=0.1175 | 4.0964 eV  f=0.1095 | 3.3206 eV  f=0.0670 | 3.3179 eV  f=0.0724 | 3.3022 eV  f=0.0621 |
| M06HF | 4.3954 eV  f=0.1650 | 4.3953 eV  f=0.1725 | 4.3780 eV  f=0.2021 | 4.3627 eV  f=0.2455 | 4.3738 eV  f=0.2030 |
|  | **CIM** | **DIB** | **DID** | **DIM** | **MAD*** |
| Expt. | 3.8196 eV | 3.8172 eV | 3.8172 eV | 3.8267 eV |  |
| LSDA | 3.4456 eV  f=0.0274 | 3.1432 eV  f=0.0470 | 3.1297 eV  f=0.0441 | 3.9456 eV  f=0.0956 | 0.5157 |
| BVP86 | 3.1677 eV  f=0.0585 | 3.1627 eV  f=0.0464 | 3.1555 eV  f=0.0461 | 3.9468 eV  f=0.0879 | 0.5334 |
| B3LYP | 4.3534 eV  f=0.1438 | 4.1803 eV  f=0.1187 | 4.1831 eV  f=0.1230 | 4.1939 eV  f=0.1054 | 0.3691 |
| **CAM-B3LYP** | **3.9538 eV**  **f=0.1413** | **3.9607 eV**  **f=0.1171** | **3.9556 eV**  **f=0.1160** | **3.9573 eV**  **f=0.1237** | **0.1442** |
| B3PW91 | 4.3954 eV  f=0.1388 | 4.2063 eV  f=0.1138 | 4.2099 eV  f=0.1186 | 4.2218 eV  f=0.1005 | 0.7396 |
| MPW1PW91 | 4.4556 eV  f=0.1642 | 4.2738 eV  f=0.1148 | 4.2737 eV  f=0.1178 | 4.2812 eV  f=0.0997 | 0.4136 |
| PBEPBE | 3.1661 eV  f=0.0578 | 3.1731 eV  f=0.0493 | 3.1613 eV  f=0.0469 | 3.9473 eV  f=0.0905 | 0.5316 |
| HSEH1PBE | 4.4204 eV  f=0.1466 | 4.2380 eV  f=0.1123 | 4.2411 eV  f=0.1171 | 4.2550 eV  f=0.1015 | 0.3783 |
| HCTH | 3.1724 eV  f=0.0570 | 3.1996 eV  f=0.0520 | 3.1924 eV  f=0.0522 | 3.9540 eV  f=0.1019 | 0.47 |
| TPSSTPSS | 3.2570 eV  f=0.0627 | 3.2679 eV  f=0.0566 | 3.2476 eV  f=0.0487 | 4.0281 eV  f=0.0934 | 0.4403 |
| 𝞈B97XD | 3.9948 eV  f=0.1451 | 3.9917 eV  f=0.1204 | 3.9873 eV  f=0.1195 | 3.9939 eV  f=0.1272 | 0.1801 |
| APFD | 4.4347 eV  f=0.1538 | 4.2501 eV  f=0.1155 | 4.2508 eV  f=0.1183 | 4.2603 eV  f=0.0997 | 0.4038 |
| BhandHLYP | 3.9962 eV  f=0.1409 | 4.0054 eV  f=0.1173 | 4.4946 eV  f=0.1165 | 4.0005 eV  f=0.1238 | 0.2428 |
| LC-𝞈PBE | 4.2580 eV  f=0.1768 | 4.2617 eV  f=0.1477 | 4.2588 eV  f=0.1467 | 4.2603 eV  f=0.1554 | 0.4475 |
| M06-2X | 3.9958 eV  f=0.1429 | 3.9918 eV  f=0.1162 | 3.9854 eV  f=0.1148 | 3.9956 eV  f=0.1234 | 0.1796 |
| M06 | 4.2891 eV  f=0.1702 | 4.1455 eV  f=0.1325 | 4.1440 eV  f=0.1367 | 4.1537 eV  f=0.1133 | 0.3257 |
| M06L | 3.3338 eV  f=0.0669 | 3.3120 eV  f=0.0554 | 3.3074 eV  f=0.0565 | 4.1046 eV  f=0.1429 | 0.4245 |
| M06HF | 4.3807 eV  f=0.2115 | 4.3954 eV  f=0.1639 | 4.3910 eV  f=0.1628 | 4.3920 eV  f=0.1741 | 0.5749 |

^*^Mean absolute deviations, $MAD=\left( \frac{1}{n} \right)\sum_{i=1}^{n} \left| E_{cal}-E_{expt} \right|$

Table S7. Excitation energy (eV) with significant oscillator strength (f) for the **R_1_IR_2_** molecules predicted using different DFT functionals with def2-TZVP basis set.

|  | **BIB** | **BIM** | **CIB** | **CIC** | **CID** |
| --- | --- | --- | --- | --- | --- |
| Expt. | 3.7754 eV | 3.8267 eV | 3.8149 eV | 3.7800 eV | 3.8126 eV |
| LSDA | 3.1420 eV  f=0.0447 | 3.9675 eV  f=0.0817 | 3.1342 eV  f=0.0547 | 3.1281 eV  f=0.0607 | 3.1191 eV  f=0.0519 |
| BVP86 | 3.8884 eV  f=0.0477 | 3.9804 eV  f=0.0690 | 3.1554 eV  f=0.0556 | 3.1493 eV  f=0.0629 | 3.1429 eV  f=0.0546 |
| B3LYP | 4.1747 eV  f=0.1045 | 4.1890 eV  f=0.0948 | 4.1834 eV  f=0.0889 | 3.5240 eV  f=0.0972 | 3.5225 eV  f=0.0841 |
| **CAM-B3LYP** | **3.9542 eV**  **f=0.1171** | **3.9549 eV**  **f=0.1207** | **3.9422 eV**  **f=0.1338** | **3.9325 eV**  **f=0.1550** | **3.9360 eV**  **f=0.1343** |
| B3PW91 | 4.2005 eV  f=0.1008 | 4.2159 eV  f=0.0914 | 4.2104 eV  f=0.0853 | 3.5557 eV  f=0.0982 | 3.5515 eV  f=0.0849 |
| MPW1PW91 | 4.2712 eV  f=0.1072 | 4.2791 eV  f=0.0925 | 3.6493 eV  f=0.0922 | 3.6436 eV  f=0.1051 | 4.2718 eV  f=0.0926 |
| PBEPBE | 3.1703 eV  f=0.0459 | 3.1738 eV  f=0.0486 | 3.1574 eV  f=0.0554 | 3.1479 eV  f=0.0621 | 3.1456 eV  f=0.0540 |
| HSEH1PBE | 4.2327 eV  f=0.0975 | 4.2503 eV  f=0.0917 | 3.5925 eV  f=0.0875 | 3.5868 eV  f=0.1018 | 3.5827 eV  f=0.0873 |
| HCTH | 3.9099 eV  f=0.0560 | 3.1851 eV  f=0.0484 | 3.1725 eV  f=0.0550 | 3.1550 eV  f=0.0623 | 3.1583 eV  f=0.0545 |
| TPSSTPSS | 4.0004 eV  f=0.0691 | 4.0149 eV  f=0.0611 | 3.2532 eV  f=0.0595 | 3.2448 eV  f=0.0684 | 3.2396 eV  f=0.0596 |
| 𝞈B97XD | 3.9837 eV  f=0.1210 | 3.9885 eV  f=0.1249 | 3.9769 eV  f=0.1379 | 3.9721 eV  f=0.1603 | 3.9717 eV  f=0.1390 |
| APFD | 4.2461 eV  f=0.1066 | 4.2561 eV  f=0.0923 | 3.6141 eV  f=0.0893 | 3.6085 eV  f=0.1025 | 4.2488 eV  f=0.0922 |
| BhandHLYP | 4.0007 eV  f=0.1174 | 4.0007 eV  f=0.1215 | 3.9877 eV  f=0.1337 | 3.9773 eV  f=0.1542 | 3.9809 eV  f=0.1341 |
| LC-𝞈PBE | 4.2458 eV  f=0.1463 | 4.2469 eV  f=0.1509 | 4.2376 eV  f=0.1668 | 4.2312 eV  f=0.1932 | 4.2336 eV  f=0.1679 |
| M06-2X | 3.9920 eV  f=0.1161 | 4.0000 eV  f=0.1206 | 3.9834 eV  f=0.1351 | 3.9791 eV  f=0.1593 | 3.9769 eV  f=0.1357 |
| M06 | 4.1346 eV  f=0.1212 | 4.1438 eV  f=0.1035 | 4.1368 eV  f=0.0994 | 3.5255 eV  f=0.0991 | 4.1341 eV  f=0.1063 |
| M06L | 4.0694 eV  f=0.0968 | 4.0809 eV  f=0.1100 | 3.3077 eV  f=0.0643 | 3.3102 eV  f=0.0738 | 3.2964 eV  f=0.0635 |
| M06HF | 4.3782 eV  f=0.1596 | 4.3789 eV  f=0.1655 | 4.3609 eV  f=0.1886 | 4.3461 eV  f=0.2251 | 4.3566 eV  f=0.1901 |
|  | **CIM** | **DIB** | **DID** | **DIM** | **MAD*** |
| Expt. | 3.8196 eV | 3.8172 eV | 3.8172 eV | 3.8267 eV |  |
| LSDA | 3.1507 eV  f=0.0566 | 3.1362 eV  f=0.0439 | 3.1248 eV  f=0.0424 | 3.9419 eV  f=0.1031 | 0.5509 |
| BVP86 | 3.1681 eV  f=0.0587 | 3.1586 eV  f=0.0456 | 3.1462 eV  f=0.0438 | 3.9458 eV  f=0.1094 | 0.4808 |
| B3LYP | 4.3656 eV  f=0.1423 | 4.1728 eV  f=0.1158 | 4.1760 eV  f=0.1209 | 4.1883 eV  f=0.1025 | 0.3665 |
| **CAM-B3LYP** | **3.9455 eV**  **f=0.1381** | **3.9526 eV**  **f=0.1163** | **3.9472 eV**  **f=0.1153** | **3.9499 eV**  **f=0.1216** | **0.1361** |
| B3PW91 | 4.3994 eV  f=0.1391 | 4.1987 eV  f=0.1121 | 4.2025 eV  f=0.1175 | 4.2156 eV  f=0.0986 | 0.3812 |
| MPW1PW91 | 4.4678 eV  f=0.1637 | 4.2688 eV  f=0.1143 | 4.2687 eV  f=0.1177 | 4.2774 eV  f=0.0984 | 0.4124 |
| PBEPBE | 3.1668 eV  f=0.0575 | 3.1660 eV  f=0.0451 | 3.1533 eV  f=0.0431 | 3.9480 eV  f=0.1063 | 0.5893 |
| HSEH1PBE | 4.4280 eV  f=0.1459 | 4.2305 eV  f=0.1101 | 4.2338 eV  f=0.1156 | 4.2495 eV  f=0.0995 | 0.3764 |
| HCTH | 3.1698 eV  f=0.0561 | 3.1884 eV  f=0.0462 | 3.1761 eV  f=0.0442 | 3.9516 eV  f=0.1111 | 0.5269 |
| TPSSTPSS | 3.2607 eV  f=0.0627 | 3.2594 eV  f=0.0497 | 3.2465 eV  f=0.0475 | 4.0313 eV  f=0.1047 | 0.4417 |
| 𝞈B97XD | 3.9843 eV  f=0.1427 | 3.9822 eV  f=0.1204 | 3.9776 eV  f=0.1194 | 3.9843 eV  f=0.1259 | 0.1701 |
| APFD | 4.4413 eV  f=0.1536 | 4.2436 eV  f=0.1145 | 4.2443 eV  f=0.1177 | 4.2546 eV  f=0.0980 | 0.4013 |
| BhandHLYP | 3.9904 eV  f=0.1382 | 3.9988 eV  f=0.1168 | 3.9926 eV  f=0.1157 | 3.9952 eV  f=0.1221 | 0.1816 |
| LC-𝞈PBE | 4.2407 eV  f=0.1719 | 4.2452 eV  f=0.1458 | 4.2421 eV  f=0.1449 | 4.2437 eV  f=0.1522 | 0.4307 |
| M06-2X | 3.9952 eV  f=0.1403 | 3.9895 eV  f=0.1153 | 3.9830 eV  f=0.1142 | 3.9948 eV  f=0.1215 | 0.1782 |
| M06 | 4.3208 eV  f=0.1715 | 4.1315 eV  f=0.1309 | 4.1308 eV  f=0.1350 | 4.1417 eV  f=0.1107 | 0.3354 |
| M06L | 3.3262 eV  f=0.0664 | 3.3043 eV  f=0.0541 | 3.2931 eV  f=0.0525 | 4.0925 eV  f=0.1400 | 0.4264 |
| M06HF | 4.3642 eV  f=0.1950 | 4.3777 eV  f=0.1588 | 4.3737 eV  f=0.1578 | 4.3758 eV  f=0.1672 | 0.558 |

^*^Mean absolute deviations, $MAD=\left( \frac{1}{n} \right)\sum_{i=1}^{n} \left| E_{cal}-E_{expt} \right|$

Table S8. Excitation energy (eV) with significant oscillator strength (f) for the **R_1_IR_2_** molecules predicted using different DFT functionals with cc-PVTZ basis set.

|  | **BIB** | **BIM** | **CIB** | **CIC** | **CID** |
| --- | --- | --- | --- | --- | --- |
| Expt. | 3.7754 eV | 3.8267 eV | 3.8149 eV | 3.7800 eV | 3.8126 eV |
| LSDA | 3.1488 eV  f=0.0451 | 3.9763 eV  f=0.0773 | 3.1380 eV  f=0.0533 | 3.1323 eV  f=0.0611 | 3.1253 eV  f=0.0521 |
| BVP86 | 3.8937 eV  f=0.0487 | 3.9901 eV  f=0.0630 | 3.1586 eV  f=0.0547 | 3.1523 eV  f=0.0628 | 3.1465 eV  f=0.0545 |
| B3LYP | 4.1823 eV  f=0.1053 | 4.1966 eV  f=0.0950 | 4.1905 eV  f=0.0894 | 3.5307 eV  f=0.0975 | 3.5299 eV  f=0.0848 |
| **CAM-B3LYP** | **3.9633 eV**  **f=0.1182** | **3.9647 eV**  **f=0.1212** | **3.9513 eV**  **f=0.1345** | **3.9414 eV**  **f=0.1555** | **3.9450 eV**  **f=0.1351** |
| B3PW91 | 4.2066 eV  f=0.1013 | 4.2219 eV  f=0.0916 | 3.5664 eV  f=0.0857 | 3.5608 eV  f=0.0986 | 3.5570 eV  f=0.0856 |
| MPW1PW91 | 4.2774 eV  f=0.1076 | 4.2857 eV  f=0.0927 | 3.6559 eV  f=0.0932 | 3.6501 eV  f=0.1059 | 3.6468 eV  f=0.0932 |
| PBEPBE | 3.8979 eV  f=0.0470 | 3.9931 eV  f=0.0625 | 3.1625 eV  f=0.0545 | 3.1524 eV  f=0.0627 | 3.1500 eV  f=0.0532 |
| HSEH1PBE | 4.2401 eV  f=0.0986 | 4.2576 eV  f=0.0922 | 3.5997 eV  f=0.0882 | 3.5931 eV  f=0.1022 | 3.5898 eV  f=0.0881 |
| HCTH | 3.9179 eV  f=0.0569 | 3.1925 eV  f=0.0487 | 3.1780 eV  f=0.0546 | 3.1589 eV  f=0.0615 | 3.1645 eV  f=0.0539 |
| TPSSTPSS | 4.0084 eV  f=0.0744 | 4.0231 eV  f=0.0669 | 3.2580 eV  f=0.0597 | 3.2495 eV  f=0.0685 | 3.2447 eV  f=0.0588 |
| 𝞈B97XD | 3.9912 eV  f=0.1220 | 3.9966 eV  f=0.1254 | 3.9844 eV  f=0.1388 | 3.9794 eV  f=0.1606 | 3.9790 eV  f=0.1397 |
| APFD | 4.2527 eV  f=0.1069 | 4.2629 eV  f=0.0924 | 4.2568 eV  f=0.0875 | 3.6145 eV  f=0.1030 | 4.2550 eV  f=0.0930 |
| BhandHLYP | 4.0102 eV  f=0.1185 | 4.0111 eV  f=0.1223 | 3.9974 eV  f=0.1347 | 3.9867 eV  f=0.1549 | 3.9905 eV  f=0.1352 |
| LC-𝞈PBE | 4.2525 eV  f=0.1475 | 4.2541 eV  f=0.1515 | 4.2444 eV  f=0.1676 | 4.2378 eV  f=0.1935 | 4.2403 eV  f=0.1687 |
| M06-2X | 3.9931 eV  f=0.1179 | 4.0021 eV  f=0.1218 | 3.9850 eV  f=0.1363 | 3.9809 eV  f=0.1598 | 3.9785 eV  f=0.1369 |
| M06 | 4.1338 eV  f=0.1146 | 4.1429 eV  f=0.0977 | 4.3412 eV  f=0.1646 | 3.5388 eV  f=0.0996 | 4.1332 eV  f=0.1006 |
| M06L | 4.0528 eV  f=0.0583 | 4.0818 eV  f=0.1047 | 3.3169 eV  f=0.0648 | 3.3211 eV  f=0.0735 | 3.3056 eV  f=0.0642 |
| M06HF | 4.3629 eV  f=0.1621 | 4.3649 eV  f=0.1670 | 4.3463 eV  f=0.1885 | 4.3318 eV  f=0.2221 | 4.3421 eV  f=0.1900 |
|  | **CIM** | **DIB** | **DID** | **DIM** | **MAD*** |
| Expt. | 3.8196 eV | 3.8172 eV | 3.8172 eV | 3.8267 eV |  |
| LSDA | 3.1555 eV  f=0.0559 | 3.1428 eV  f=0.0440 | 3.1293 eV  f=0.0424 | 3.9480 eV  f=0.1017 | 0.5484 |
| BVP86 | 3.1707 eV  f=0.0577 | 3.1630 eV  f=0.0458 | 3.1508 eV  f=0.0439 | 3.9512 eV  f=0.1084 | 0.4806 |
| B3LYP | 4.3869 eV  f=0.1386 | 4.1803 eV  f=0.1164 | 4.1834 eV  f=0.1216 | 4.1959 eV  f=0.1027 | 0.3722 |
| **CAM-B3LYP** | **3.9552 eV**  **f=0.1384** | **3.9615 eV**  **f=0.1173** | **3.9560 eV**  **f=0.1164** | **3.9596 eV**  **f=0.1222** | **0.1453** |
| B3PW91 | 4.4161 eV  f=0.1356 | 4.2048 eV  f=0.1124 | 4.2085 eV  f=0.1179 | 4.2216 eV  f=0.0989 | 0.3689 |
| MPW1PW91 | 4.4859 eV  f=0.1600 | 4.2750 eV  f=0.1147 | 4.2747 eV  f=0.1182 | 4.2839 eV  f=0.0986 | 0.3838 |
| PBEPBE | 3.1713 eV  f=0.0569 | 3.1714 eV  f=0.0457 | 3.1585 eV  f=0.0440 | 3.9545 eV  f=0.1062 | 0.4791 |
| HSEH1PBE | 3.6087 eV  f=0.0920 | 4.2379 eV  f=0.1110 | 4.2413 eV  f=0.1168 | 4.2569 eV  f=0.1002 | 0.334 |
| HCTH | 3.1759 eV  f=0.0553 | 3.1942 eV  f=0.0462 | 3.1817 eV  f=0.0447 | 3.9592 eV  f=0.1119 | 0.5242 |
| TPSSTPSS | 3.2667 eV  f=0.0630 | 3.2638 eV  f=0.0501 | 3.2502 eV  f=0.0478 | 4.0378 eV  f=0.1052 | 0.441 |
| 𝞈B97XD | 3.9924 eV  f=0.1428 | 3.9895 eV  f=0.1214 | 3.9847 eV  f=0.1204 | 3.9922 eV  f=0.1264 | 0.1777 |
| APFD | 4.4595 eV  f=0.1498 | 4.2502 eV  f=0.1148 | 4.2508 eV  f=0.1181 | 4.2613 eV  f=0.0983 | 0.4338 |
| BhandHLYP | 4.0008 eV  f=0.1388 | 4.0081 eV  f=0.1179 | 4.0018 eV  f=0.1170 | 4.0054 eV  f=0.1230 | 0.1913 |
| LC-𝞈PBE | 4.2480 eV  f=0.1721 | 4.2517 eV  f=0.1470 | 4.2486 eV  f=0.1462 | 4.2509 eV  f=0.1528 | 0.4376 |
| M06-2X | 3.9978 eV  f=0.1409 | 3.9905 eV  f=0.1171 | 3.9840 eV  f=0.1160 | 3.9970 eV  f=0.1227 | 0.1798 |
| M06 | 4.3416 eV  f=0.1674 | 4.1308 eV  f=0.1235 | 4.1296 eV  f=0.1277 | 4.1409 eV  f=0.1044 | 0.3583 |
| M06L | 3.3382 eV  f=0.0675 | 3.3107 eV  f=0.0551 | 3.2989 eV  f=0.0537 | 4.0932 eV  f=0.1343 | 0.4188 |
| M06HF | 4.3506 eV  f=0.1936 | 4.3624 eV  f=0.1615 | 4.3583 eV  f=0.1607 | 4.3618 eV  f=0.1687 | 0.5434 |

^*^Mean absolute deviations, $MAD=\left( \frac{1}{n} \right)\sum_{i=1}^{n} \left| E_{cal}-E_{expt} \right|$

Table S9. Excitation energy (eV) with significant oscillator strength (f) for the **R_1_SR_2_** molecules predicted using different DFT functionals with 6-31+G(2d,p) basis set.

|  | **BSB** | **BSM** | **CSB** | **CSC** | **CSD** |
| --- | --- | --- | --- | --- | --- |
| Expt. | 3.5751 eV | 3.5689 eV | 3.5123 eV | 3.5587 eV | 3.5648 eV |
| LSDA | 3.4135 eV  f=0.3907 | 3.3816 eV  f=0.4068 | 3.3919 eV  f=0.3553 | 3.3786 eV  f=0.3739 | 3.3874 eV  f=0.2782 |
| BVP86 | 3.4440 eV  f=0.3972 | 3.4133 eV  f=0.3722 | 3.4182 eV  f=0.2726 | 3.4095 eV  f=0.3078 | 3.4145 eV  f=0.2423 |
| B3LYP | 3.7942 eV  f=0.4352 | 3.7789 eV  f=0.4017 | 3.7705 eV  f=0.3558 | 3.7563 eV  f=0.3683 | 3.7721 eV  f=0.3456 |
| CAM-B3LYP | 4.0883 eV  f=0.5032 | 4.0751 eV  f=0.5497 | 4.0726 eV  f=0.4805 | 4.0613 eV  f=0.4746 | 4.0749 eV  f=0.4763 |
| B3PW91 | 3.8154 eV  f=0.4318 | 3.7994 eV  f=0.3948 | 3.7908 eV  f=0.3514 | 3.7760 eV  f=0.3666 | 3.7925 eV  f=0.3414 |
| MPW1PW91 | 3.8985 eV  f=0.4842 | 3.8790 eV  f=0.4222 | 3.8942 eV  f=0.3336 | 3.8508 eV  f=0.4234 | 3.8952 eV  f=0.3381 |
| PBEPBE | 3.4367 eV  f=0.3949 | 3.4068 eV  f=0.3872 | 3.4134 eV  f=0.3084 | 3.4024 eV  f=0.3706 | 3.4100 eV  f=0.2727 |
| HSEH1PBE | 3.8498 eV  f=0.4569 | 3.8319 eV  f=0.4107 | 3.8226 eV  f=0.3642 | 3.8082 eV  f=0.4181 | 3.8243 eV  f=0.3520 |
| HCTH | 3.4589 eV  f=0.4081 | 3.4303 eV  f=0.3852 | 3.4340 eV  f=0.3228 | 3.4206 eV  f=0.3534 | 3.4329 eV  f=0.2867 |
| TPSSTPSS | **3.5516 eV**  **f=0.3909** | **3.5387 eV**  **f=0.2849** | **3.5380 eV**  **f=0.2760** | **3.5193 eV**  **f=0.3039** | **3.5401 eV**  **f=0.2707** |
| 𝞈B97XD | 4.1226 eV  f=0.4883 | 4.1095 eV  f=0.5310 | 4.1078 eV  f=0.4652 | 4.0977 eV  f=0.4600 | 4.1102 eV  f=0.4612 |
| APFD | 3.8659 eV  f=0.4688 | 3.8474 eV  f=0.4193 | 3.8483 eV  f=0.3685 | 3.7994 eV  f=0.2237 | 3.8492 eV  f=0.3569 |
| BhandHLYP | 4.2197 eV  f=0.5256 | 4.2006 eV  f=0.4951 | 4.1953 eV  f=0.3976 | 4.1902 eV  f=0.4940 | 4.1966 eV  f=0.3901 |
| LC-𝞈PBE | 4.2368 eV  f=0.4508 | 4.2261 eV  f=0.4905 | 4.2255 eV  f=0.4353 | 4.2157 eV  f=0.4221 | 4.2278 eV  f=0.4322 |
| M06-2X | 4.0734 eV  f=0.5182 | 4.0640 eV  f=0.5880 | 4.0587 eV  f=0.5026 | 4.0469 eV  f=0.4953 | 4.0614 eV  f=0.4969 |
| M06 | 3.8296 eV  f=0.4312 | 3.7892 eV  f=0.1459 | 3.8158 eV  f=0.3435 | 3.7869 eV  f=0.4096 | 3.7790 eV  f=0.1607 |
| **M06L** | **3.6964 eV**  **f=0.4004** | **3.6764 eV**  **f=0.3974** | **3.6818 eV**  **f=0.2131** | **3.6693 eV**  **f=0.3549** | **3.6873 eV**  **f=0.2065** |
| M06HF | 4.1608 eV  f=0.3477 | 4.1806 eV  f=0.6170 | 4.1654 eV  f=0.4520 | 4.1659 eV  f=0.4904 | 4.1659 eV  f=0.4311 |
|  | **CSM** | **DSB** | **DSD** | **DSM** | **MAD*** |
| Expt. | 3.5587 eV | 3.5283 eV | 3.5772 eV | 3.5730 eV |  |
| LSDA | 3.3686 eV  f=0.4228 | 3.4086 eV  f=0.3414 | 3.4103 eV  f=0.3106 | 3.3758 eV  f=0.3578 | 0.1667 |
| BVP86 | 3.4007 eV  f=0.3977 | 3.4413 eV  f=0.3322 | 3.4417 eV  f=0.3215 | 3.4080 eV  f=0.3322 | 0.1362 |
| B3LYP | 3.7695 eV  f=0.4818 | 3.7963 eV  f=0.4173 | 3.7982 eV  f=0.4161 | 3.7796 eV  f=0.3780 | 0.2221 |
| CAM-B3LYP | 4.0637 eV  f=0.5336 | 4.0906 eV  f=0.4997 | 4.0927 eV  f=0.4941 | 4.0775 eV  f=0.5462 | 0.52 |
| B3PW91 | 3.7897 eV  f=0.4761 | 3.8176 eV  f=0.4132 | 3.8195 eV  f=0.4122 | 3.8000 eV  f=0.3723 | 0.2427 |
| MPW1PW91 | 3.8749 eV  f=0.3341 | 3.9007 eV  f=0.4580 | 3.9031 eV  f=0.4683 | 3.8798 eV  f=0.4026 | 0.3288 |
| PBEPBE | 3.3953 eV  f=0.4019 | 3.4312 eV  f=0.1806 | 3.4361 eV  f=0.3284 | 3.4032 eV  f=0.3476 | 0.1424 |
| HSEH1PBE | 3.8207 eV  f=0.4861 | 3.8519 eV  f=0.4198 | 3.8541 eV  f=0.4378 | 3.8325 eV  f=0.3867 | 0.2754 |
| HCTH | 3.4160 eV  f=0.3245 | 3.4584 eV  f=0.3392 | 3.4610 eV  f=0.3579 | 3.4291 eV  f=0.3551 | 0.1195 |
| **TPSSTPSS** | **3.5020 eV**  **f=0.2911** | **3.5500 eV**  **f=0.3605** | **3.5528 eV**  **f=0.3145** | **3.5411 eV**  **f=0.2505** | **0.0309** |
| 𝞈B97XD | 4.0992 eV  f=0.5162 | 4.1250 eV  f=0.4848 | 4.1270 eV  f=0.4795 | 4.1117 eV  f=0.5279 | 0.5549 |
| APFD | 3.8416 eV  f=0.4370 | 3.8682 eV  f=0.4473 | 3.8704 eV  f=0.4514 | 3.8482 eV  f=0.3983 | 0.2913 |
| BhandHLYP | 4.1929 eV  f=0.5428 | 4.2219 eV  f=0.5174 | 4.2243 eV  f=0.5175 | 4.2017 eV  f=0.4812 | 0.6474 |
| LC-𝞈PBE | 4.2164 eV  f=0.4722 | 4.2391 eV  f=0.4477 | 4.2406 eV  f=0.4437 | 4.2285 eV  f=0.4881 | 0.6711 |
| M06-2X | 4.0526 eV  f=0.5668 | 4.0755 eV  f=0.5124 | 4.0769 eV  f=0.5007 | 4.0672 eV  f=0.5828 | 0.5066 |
| M06 | 3.8009 eV  f=0.5001 | 3.8316 eV  f=0.4216 | 3.8328 eV  f=0.4083 | 3.8275 eV  f=0.4249 | 0.2529 |
| **M06L** | **3.6233 eV**  **f=0.1348** | **3.6954 eV**  **f=0.3830** | **3.6994 eV**  **f=0.3385** | **3.6796 eV**  **f=0.3564** | **0.1213** |
| M06HF | 4.1760 eV  f=0.5996 | 4.1599 eV  f=0.3211 | 4.2392 eV  f=0.3379 | 4.1832 eV  f=0.6014 | 0.62 |

^*^Mean absolute deviations, $MAD=\left( \frac{1}{n} \right)\sum_{i=1}^{n} \left| E_{cal}-E_{expt} \right|$

Table S10. Excitation energy (eV) with significant oscillator strength (f) for the **R_1_SR_2_** molecules predicted using different DFT functionals with def2-TZVP basis set.

|  | **BSB** | **BSM** | **CSB** | **CSC** | **CSD** |
| --- | --- | --- | --- | --- | --- |
| Expt. | 3.5751 eV | 3.5689 eV | 3.5123 eV | 3.5587 eV | 3.5648 eV |
| LSDA | 3.3939 eV  f=0.3686 | 3.3666 eV  f=0.3916 | 3.3751 eV  f=0.3464 | 3.3786 eV  f=0.3739 | 3.3689 eV  f=0.3029 |
| BVP86 | 3.4282 eV  f=0.3731 | 3.4032 eV  f=0.3861 | 3.4081 eV  f=0.3365 | 3.3963 eV  f=0.3615 | 3.4041 eV  f=0.2984 |
| B3LYP | 3.7859 eV  f=0.3185 | 3.7841 eV  f=0.4473 | 3.7650 eV  f=0.2847 | 3.7502 eV  f=0.2474 | 3.7672 eV  f=0.2857 |
| CAM-B3LYP | 4.0931 eV  f=0.4605 | 4.0837 eV  f=0.5003 | 4.0787 eV  f=0.4377 | 4.0690 eV  f=0.4308 | 4.0807 eV  f=0.4342 |
| B3PW91 | 3.8063 eV  f=0.3454 | 3.8006 eV  f=0.4266 | 3.7842 eV  f=0.3019 | 3.7688 eV  f=0.2754 | 3.7863 eV  f=0.3010 |
| MPW1PW91 | 3.8982 eV  f=0.4321 | 3.8850 eV  f=0.4247 | 3.8568 eV  f=0.2458 | 3.8554 eV  f=0.3986 | 3.9015 eV  f=0.2439 |
| PBEPBE | 3.4258 eV  f=0.3760 | 3.4006 eV  f=0.3895 | 3.4053 eV  f=0.3146 | 3.3932 eV  f=0.3594 | 3.4018 eV  f=0.3063 |
| HSEH1PBE | 3.8429 eV  f=0.3705 | 3.8352 eV  f=0.4420 | 3.8186 eV  f=0.3304 | 3.8026 eV  f=0.3389 | 3.8208 eV  f=0.3263 |
| HCTH | 3.4524 eV  f=0.3760 | 3.4293 eV  f=0.3881 | 3.4310 eV  f=0.3472 | 3.4166 eV  f=0.3605 | 3.4294 eV  f=0.3189 |
| **TPSSTPSS** | **3.5455 eV**  **f=0.3864** | **3.5112 eV**  **f=0.3093** | **3.5130 eV**  **f=0.1587** | **3.5118 eV**  **f=0.3177** | **3.5358 eV**  **f=0.1838** |
| 𝞈B97XD | 4.1246 eV  f=0.4518 | 4.1146 eV  f=0.4871 | 4.1110 eV  f=0.4273 | 4.1025 eV  f=0.4208 | 4.1130 eV  f=0.4245 |
| APFD | 3.8616 eV  f=0.4055 | 3.8507 eV  f=0.4290 | 3.8483 eV  f=0.3030 | 3.8105 eV  f=0.3202 | 3.8490 eV  f=0.3083 |
| BhandHLYP | 4.2267 eV  f=0.4856 | 4.2102 eV  f=0.4379 | 4.2027 eV  f=0.3504 | 4.1998 eV  f=0.4523 | 4.2038 eV  f=0.3456 |
| LC-𝞈PBE | 4.2362 eV  f=0.4223 | 4.2279 eV  f=0.4554 | 4.2259 eV  f=0.4056 | 4.2173 eV  f=0.3913 | 4.2279 eV  f=0.4028 |
| M06-2X | 4.0819 eV  f=0.4645 | 4.0771 eV  f=0.5373 | 4.0690 eV  f=0.4545 | 4.0596 eV  f=0.4497 | 4.0713 eV  f=0.4494 |
| M06 | 3.8194 eV  f=0.3610 | 3.8055 eV  f=0.2505 | 3.7908 eV  f=0.2589 | 3.7836 eV  f=0.3452 | 3.7923 eV  f=0.2520 |
| M06L | **3.6814 eV**  **f=0.4041** | **3.6252 eV**  **f=0.1464** | **3.6706 eV**  **f=0.3807** | **3.6574 eV**  **f=0.3682** | **3.6729 eV**  **f=0.3627** |
| M06HF | 4.1931 eV  f=0.2820 | 4.2211 eV  f=0.5809 | 4.2044 eV  f=0.4178 | 4.2102 eV  f=0.4661 | 4.2040 eV  f=0.3933 |
|  | **CSM** | **DSB** | **DSD** | **DSM** | **MAD*** |
| Expt. | 3.5587 eV | 3.5283 eV | 3.5772 eV | 3.5730 eV |  |
| LSDA | 3.3543 eV  f=0.4035 | 3.3893 eV  f=0.3353 | 3.3902 eV  f=0.3049 | 3.3603 eV  f=0.3406 | 0.1822 |
| BVP86 | 3.3914 eV  f=0.4056 | 3.4250 eV  f=0.3398 | 3.4272 eV  f=0.3161 | 3.3986 eV  f=0.3456 | 0.1483 |
| B3LYP | 3.7680 eV  f=0.2861 | 3.7880 eV  f=0.3143 | 3.7890 eV  f=0.3059 | 3.7852 eV  f=0.4271 | 0.2184 |
| CAM-B3LYP | 4.0732 eV  f=0.4818 | 4.0951 eV  f=0.4567 | 4.0968 eV  f=0.4515 | 4.0858 eV  f=0.4978 | 0.5266 |
| B3PW91 | 3.7866 eV  f=0.3359 | 3.8086 eV  f=0.3407 | 3.8096 eV  f=0.3296 | 3.8015 eV  f=0.4024 | 0.2373 |
| MPW1PW91 | 3.8634 eV  f=0.2932 | 3.9004 eV  f=0.4243 | 3.9020 eV  f=0.4149 | 3.8858 eV  f=0.4060 | 0.3257 |
| PBEPBE | 3.3883 eV  f=0.4049 | 3.4221 eV  f=0.3441 | 3.4242 eV  f=0.3176 | 3.3960 eV  f=0.3494 | 0.1511 |
| HSEH1PBE | 3.8187 eV  f=0.3681 | 3.8453 eV  f=0.3654 | 3.8465 eV  f=0.3535 | 3.8361 eV  f=0.4182 | 0.2722 |
| HCTH | 3.4143 eV  f=0.3978 | 3.4509 eV  f=0.3534 | 3.4540 eV  f=0.3323 | 3.4270 eV  f=0.3583 | 0.1236 |
| **TPSSTPSS** | **3.5038 eV**  **f=0.3916** | **3.5438 eV**  **f=0.3550** | **3.5462 eV**  **f=0.3222** | **3.5074 eV**  **f=0.2836** | **0.0368** |
| 𝞈B97XD | 4.1054 eV  f=0.4697 | 4.1267 eV  f=0.4488 | 4.1284 eV  f=0.4445 | 4.1165 eV  f=0.4845 | 0.5584 |
| APFD | 3.8462 eV  f=0.3534 | 3.8638 eV  f=0.3992 | 3.8652 eV  f=0.3877 | 3.8516 eV  f=0.4089 | 0.2922 |
| BhandHLYP | 4.2036 eV  f=0.4727 | 4.2287 eV  f=0.4774 | 4.2309 eV  f=0.4787 | 4.2110 eV  f=0.4266 | 0.6556 |
| LC-𝞈PBE | 4.2190 eV  f=0.4355 | 4.2383 eV  f=0.4196 | 4.2396 eV  f=0.4163 | 4.2300 eV  f=0.4536 | 0.6717 |
| M06-2X | 4.0668 eV  f=0.5156 | 4.0835 eV  f=0.4576 | 4.0840 eV  f=0.4422 | 4.0799 eV  f=0.5333 | 0.5173 |
| M06 | 3.8034 eV  f=0.3571 | 3.8203 eV  f=0.2951 | 3.8215 eV  f=0.3374 | 3.8057 eV  f=0.2470 | 0.2473 |
| **M06L** | **3.6240 eV**  **f=0.1466** | **3.6811 eV**  **f=0.3942** | **3.6850 eV**  **f=0.3671** | **3.6696 eV**  **f=0.3400** | **0.1056** |
| M06HF | 4.2201 eV  f=0.5684 | 4.2695 eV  f=0.3264 | 4.1799 eV  f=0.2007 | 4.2226 eV  f=0.5604 | 0.6564 |

^*^Mean absolute deviations, $MAD=\left( \frac{1}{n} \right)\sum_{i=1}^{n} \left| E_{cal}-E_{expt} \right|$

Table S11. Excitation energy (eV) with significant oscillator strength (f) for the **R_1_SR_2_** molecules predicted using different DFT functionals with cc-PVTZ basis set.

|  | **BSB** | **BSM** | **CSB** | **CSC** | **CSD** |
| --- | --- | --- | --- | --- | --- |
| Expt. | 3.5751 eV | 3.5689 eV | 3.5123 eV | 3.5587 eV | 3.5648 eV |
| LSDA | 3.4046 eV  f=0.3650 | 3.3780 eV  f=0.3904 | 3.3861 eV  f=0.3460 | 3.3786 eV  f=0.3739 | 3.3804 eV  f=0.3030 |
| BVP86 | 3.4395 eV  f=0.3717 | 3.4142 eV  f=0.3811 | 3.4195 eV  f=0.3313 | 3.4077 eV  f=0.3498 | 3.4153 eV  f=0.2917 |
| B3LYP | 3.8023 eV  f=0.3420 | 3.7981 eV  f=0.4320 | 3.7810 eV  f=0.3016 | 3.7661 eV  f=0.2791 | 3.7830 eV  f=0.3005 |
| **CAM-B3LYP** | 4.1075 eV  f=0.4597 | 4.0975 eV  f=0.4957 | 4.0924 eV  f=0.4346 | 4.0820 eV  f=0.4268 | 4.0945 eV  f=0.4313 |
| B3PW91 | 3.8204 eV  f=0.3656 | 3.8121 eV  f=0.4074 | 3.7977 eV  f=0.3140 | 3.7825 eV  f=0.2996 | 3.7997 eV  f=0.3108 |
| MPW1PW91 | 3.9119 eV  f=0.4410 | 3.8968 eV  f=0.4063 | 3.9112 eV  f=0.2631 | 3.8675 eV  f=0.3961 | 3.9126 eV  f=0.2750 |
| PBEPBE | 3.4375 eV  f=0.3694 | 3.4131 eV  f=0.3848 | 3.4182 eV  f=0.3394 | 3.4056 eV  f=0.3510 | 3.4143 eV  f=0.3015 |
| HSEH1PBE | 3.8581 eV  f=0.3896 | 3.8481 eV  f=0.4249 | 3.8332 eV  f=0.3375 | 3.8178 eV  f=0.3542 | 3.8352 eV  f=0.3316 |
| HCTH | 3.4657 eV  f=0.3711 | 3.4431 eV  f=0.3831 | 3.4447 eV  f=0.3430 | 3.4299 eV  f=0.3232 | 3.4425 eV  f=0.3110 |
| **TPSSTPSS** | **3.5564 eV**  **f=0.3785** | **3.5231 eV**  **f=0.3027** | **3.5468 eV**  **f=0.2635** | **3.5248 eV**  **f=0.3545** | **3.5483 eV**  **f=0.2347** |
| 𝞈B97XD | 4.1372 eV  f=0.4480 | 4.1265 eV  f=0.4795 | 4.1229 eV  f=0.4217 | 4.1137 eV  f=0.4143 | 4.1249 eV  f=0.4190 |
| APFD | 3.8756 eV  f=0.4176 | 3.8627 eV  f=0.4124 | 3.8607 eV  f=0.3229 | 3.8195 eV  f=0.2780 | 3.8615 eV  f=0.3221 |
| BhandHLYP | 4.2414 eV  f=0.4838 | 4.2240 eV  f=0.4234 | 4.2161 eV  f=0.3363 | 4.2131 eV  f=0.4477 | 4.2171 eV  f=0.3321 |
| LC-𝞈PBE | 4.2485 eV  f=0.4197 | 4.2395 eV  f=0.4503 | 4.2374 eV  f=0.4014 | 4.2280 eV  f=0.3862 | 4.2395 eV  f=0.3987 |
| M06-2X | 4.0965 eV  f=0.4723 | 4.0909 eV  f=0.5441 | 4.0824 eV  f=0.4593 | 4.0719 eV  f=0.4535 | 4.0847 eV  f=0.4542 |
| M06 | 3.8365 eV  f=0.3970 | 3.8177 eV  f=0.2605 | 3.8051 eV  f=0.2427 | 3.8009 eV  f=0.3712 | 3.8062 eV  f=0.2356 |
| **M06L** | **3.6835 eV**  **f=0.3951** | **3.6692 eV**  **f=0.3373** | **3.6732 eV**  **f=0.3683** | **3.6599 eV**  **f=0.3549** | **3.6753 eV**  **f=0.3510** |
| M06HF | 4.2852 eV  f=0.4149 | 4.2392 eV  f=0.5358 | 4.2163 eV  f=0.3506 | 4.2246 eV  f=0.4421 | 4.2150 eV  f=0.3199 |
|  | **CSM** | **DSB** | **DSD** | **DSM** | **MAD*** |
| Expt. | 3.5587 eV | 3.5283 eV | 3.5772 eV | 3.5730 eV |  |
| LSDA | 3.3654 eV  f=0.3977 | 3.4004 eV  f=0.3335 | 3.4007 eV  f=0.3016 | 3.3713 eV  f=0.3380 | 0.1724 |
| BVP86 | 3.4025 eV  f=0.3952 | 3.4361 eV  f=0.3360 | 3.4379 eV  f=0.3129 | 3.4095 eV  f=0.3402 | 0.1372 |
| B3LYP | 3.7830 eV  f=0.3245 | 3.8046 eV  f=0.3379 | 3.8054 eV  f=0.3279 | 3.7989 eV  f=0.4076 | 0.2339 |
| CAM-B3LYP | 4.0862 eV  f=0.4763 | 4.1097 eV  f=0.4566 | 4.1112 eV  f=0.4520 | 4.0995 eV  f=0.4932 | 0.5404 |
| B3PW91 | 3.7991 eV  f=0.3660 | 3.8229 eV  f=0.3608 | 3.8238 eV  f=0.3499 | 3.8127 eV  f=0.3842 | 0.2504 |
| MPW1PW91 | 3.8928 eV  f=0.2910 | 3.9142 eV  f=0.4312 | 3.9158 eV  f=0.4253 | 3.8975 eV  f=0.3883 | 0.3448 |
| PBEPBE | 3.4007 eV  f=0.3952 | 3.4347 eV  f=0.3409 | 3.4361 eV  f=0.3137 | 3.4086 eV  f=0.3452 | 0.1387 |
| HSEH1PBE | 3.8327 eV  f=0.3869 | 3.8606 eV  f=0.3843 | 3.8617 eV  f=0.3726 | 3.8696 eV  f=0.1380 | 0.2889 |
| HCTH | 3.4283 eV  f=0.3640 | 3.4643 eV  f=0.3479 | 3.4670 eV  f=0.3276 | 3.4405 eV  f=0.3522 | 0.1101 |
| **TPSSTPSS** | **3.5159 eV**  **f=0.3871** | **3.5557 eV**  **f=0.3504** | **3.5575 eV**  **f=0.3175** | **3.5191 eV**  **f=0.2776** | **0.0326** |
| 𝞈B97XD | 4.1165 eV  f=0.4611 | 4.1393 eV  f=0.4455 | 4.1410 eV  f=0.4418 | 4.1283 eV  f=0.4775 | 0.5704 |
| APFD | 3.8572 eV  f=0.3753 | 3.8780 eV  f=0.4107 | 3.8793 eV  f=0.4014 | 3.8635 eV  f=0.3928 | 0.3046 |
| BhandHLYP | 4.2170 eV  f=0.4565 | 4.2435 eV  f=0.4725 | 4.2455 eV  f=0.4772 | 4.2247 eV  f=0.4126 | 0.6695 |
| LC-𝞈PBE | 4.2298 eV  f=0.4296 | 4.2507 eV  f=0.4173 | 4.2519 eV  f=0.4145 | 4.2416 eV  f=0.4487 | 0.6833 |
| M06-2X | 4.0794 eV  f=0.5201 | 4.0982 eV  f=0.4658 | 4.0986 eV  f=0.4488 | 4.0936 eV  f=0.5403 | 0.531 |
| M06 | 3.8152 eV  f=0.3733 | 3.8389 eV  f=0.3522 | 3.8394 eV  f=0.3784 | 3.8180 eV  f=0.2551 | 0.2623 |
| M06L | **3.6533 eV**  **f=0.3174** | **3.6833 eV**  **f=0.3873** | **3.6874 eV**  **f=0.3601** | **3.6728 eV**  **f=0.3132** | **0.1157** |
| M06HF | 4.2380 eV  f=0.5696 | 4.2861 eV  f=0.4312 | 4.2863 eV  f=0.4657 | 4.2398 eV  f=0.4831 | 0.6904 |

^*^Mean absolute deviations, $MAD=\left( \frac{1}{n} \right)\sum_{i=1}^{n} \left| E_{cal}-E_{expt} \right|$

Table S12. Calculated electronic transition with significant oscillator strength (*f*) of **R_1_IR_2_** (CAM-B3LYP/Def-2-TZVP).

|  | **BIB** | **BIM** | **CIB** | **CIC** | **CID** |
| --- | --- | --- | --- | --- | --- |
| **S_1_** | **3.9542 eV; f=0.1171** | **3.9549 eV; f=0.1207**  [H]→[L]: 96.4% | **3.9422 eV; f=0.1338**  [H]→[L]: 95.9% | **3.9325 eV; f=0.1550**  [H-5] → [L+3]: 2.7%  [H-4] → [L]: 95.5% | **3.9360 eV; f=0.1343**  [H-3] → [L+4]: 2.3%  [H-2] → [L]: 95.8% |
| **S_2_** | 4.3794 eV; f=0.0870 | 4.3819 eV; f=0.0768 | 4.1921 eV; f=0.0623 | 4.1919 eV; f=0.1110 | 4.1916 eV; f=0.0624 |
|  | **CIM** | **DIB** | **DID** | **DIM** |  |
| **S_1_** | **3.9455 eV; f=0.1381**  [H-3] → [L+2]: 2.8%  [H-2] → [L]: 96.0% | **3.9526 eV; f=0.1163**  [H]→[L]: 96.2% | **3.9472 eV; f=0.1153**  [H]→[L]: 96.1% | **3.9499 eV; f=0.1216**  [H-1] → [L+3]: 2.2%  [H]→[L]: 96.3% |  |
| **S_2_** | 4.1923 eV; f=0.0613 | 4.3787 eV; f=0.0922 | 4.3779 eV; f=0.0958 | 4.3806 eV; f=0.0815 |  |

Table S13. Calculated electronic transition with significant oscillator strength (*f*) of **R_1_IR_2_** (TPSSTPSS/6-31+G(2d,p)).

|  | **BSB** | **BSM** | **CSB** | **CSC** | **CSD** |
| --- | --- | --- | --- | --- | --- |
| **S_1_** | 3.4480 eV; f=0.0379 | 3.4359 eV; f=0.0100 | 2.7423 eV; f=0.0011 | 2.7811 eV; f=0.0012 | 2.7313 eV; f=0.0011 |
| **S_2_** | 3.5150 eV; f=0.0636 | 3.5064 eV; f=0.2238 | 3.2048 eV; f=0.0000 | 2.7837 eV; f=0.0009 | 3.1943 eV; f=0.0000 |
| **S_3_** | **3.5516 eV; f=0.3909**  [H-2] → [L]:13.5%  [H-1] → [L]:75.5%  [H] → [L+1]:2.5%  [H] → [L+6]:3.3% | **3.5387 eV; f=0.2849**  [H-2] → [L]:9.5%  [H-1] → [L]:81.9%  [H] → [L+3]:3.9% | 3.4190 eV; f=0.0563 | 3.2454 eV; f=0.0000 | 3.2845 eV; f=0.0000 |
| **S_4_** | 3.9183 eV; f=0.0001 | 3.8746 eV; f=0.0099 | 3.5034 eV; f=0.0861 | 3.2454 eV; f=0.0000 | 3.3404 eV; f=0.0000 |
| **S_5_** |  |  | 3.5278 eV; f=0.0581 | 3.2821 eV; f=0.0000 | 3.4250 eV; f=0.0604 |
| **S_6_** |  |  | **3.5380 eV; f=0.2761**  [H-4] → [L]: 13.6%  [H-2] → [L]: 60.1%  [H-1] → [L]:2.0%  [H-1] → [L+4]: 2.8%  [H] → [L+2]: 13.8% | 3.2821 eV; f=0.0000 | 3.4597 eV; f=0.0007 |
| **S_7_** |  |  | 3.5432 eV; f=0.0490 | 3.3894 eV; f=0.0614 | 3.5011 eV; f=0.0894 |
| **S_8_** |  |  |  | 3.4972 eV; f=0.0746 | 3.5300 eV; f=0.0647 |
| **S_9_** |  |  |  | 3.5142 eV; f=0.0633 | **3.5401 eV; f=0.2707**  [H-4] → [L]:15.1%  [H-2] → [L]:66.7%  [H-2] → [L+2]:2.6%  [H-1] → [L+5]:4.8%  [H] → [L+1]:3.0% |
| **S_10_** |  |  |  | 3.5147 eV; f=0.0259 | 3.6199 eV; f=0.0218 |
| **S_11_** |  |  |  | **3.5193 eV; f=0.3039**  [H-6] → [L]:21.5%  [H-3] → [L]:61.0%  [H-2] → [L+2]:3.1%  [H-2] → [L+3]:4.8%  [H-1] → [L+1]:2.6%  [H] → [L+2]:2.5% |  |
| **S_12_** |  |  |  | 3.5641 eV; f=0.0002 |  |
|  | **CSM** | **DSB** | **DSD** | **DSM** |  |
| **S_1_** | 2.7856 eV; f=0.0011 | 3.4523 eV; f=0.0393 | 3.4570 eV; f=0.0464 | 3.4413 eV; f=0.0103 |  |
| **S_2_** | 3.2493 eV; f=0.0000 | 3.5063 eV; f=0.0599 | 3.5053 eV; f=0.0597 | 3.5023 eV; f=0.2185 |  |
| **S_3_** | 3.4044 eV; f=0.0243 | **3.5500 eV; f=0.3605**  [H-2] → [L]:13.4%  [H-1] → [L]: 71.7%  [H] → [L+1]:3.7%  [H-2] → [L+7]:2.3% | **3.5528 eV; f=0.3145**  [H-2] → [L]:16.5%  [H-1] → [L]: 64.3%  [H-1] → [L+2]:2.2%  [H] → [L+1]:9.4%  [H] → [L+7]:9.4% | **3.5411 eV; f=0.2505**  [H-2] → [L]:10.7%  [H-1] → [L]: 76.9%  [H] → [L+4]:5.1% |  |
| **S_4_** | **3.5020 eV; f=0.2911**  [H-2] → [L]:32.1%  [H-2] → [L+2]:2.3%  [H-1] → [L]:56.5%  [H-1] → [L+2]:2.4%  [H] → [L+1]:2.6% | 3.6781 eV; f=0.0346 | 3.6855 eV; f=0.0750 | 3.6219 eV; f=0.0176 |  |
| **S_5_** | 3.5144 eV; f=0.0239 |  |  |  |  |

Table S14. Calculated natural orbitals (NTOs) of **R_1_SR_2_** for most excitation channels.

|  | **BSB** | **BSM** | **CSB** | **CSC** | **CSD** |
| --- | --- | --- | --- | --- | --- |
| LUTO |  |  |  |  |  |
| HOTO |  |  |  |  |  |
|  | S_0_ → S_3_ | S_0_ → S_3_ | S_0_ → S_6_ | S_0_ → S_11_ | S_0_ → S_9_ |
|  | **CSM** |  | **DSB** | **DSD** | **DSM** |
| LUTO |  |  |  |  |  |
| HOTO |  |  |  |  |  |
|  | S_0_ → S_4_ |  | S_0_ → S_3_ | S_0_ → S_3_ | S_0_ → S_3_ |

- 1. **Bacterial experiments results**

| a) | b) |
| --- | --- |

Figure S52. The CFU percentage of bacteria in the presence of (a) **BIB** and (b) **BSB** (1 – 128 µM).

| a) | b) |
| --- | --- |

Figure S53. The CFU percentage of bacteria in the presence of (a) **BIM** and (b) **BSM** (1 – 128 µM).

| a) | b) |
| --- | --- |

Figure S54. The CFU percentage of bacteria in the presence of (a) **CIB** and (b) **CSB** (1 – 128 µM).

| a) | b) |
| --- | --- |

Figure S55. The CFU percentage of bacteria in the presence of (a) **CIC** and (b) **CSC** (1 – 128 µM).

| a) | b) |
| --- | --- |

Figure S56. The CFU percentage of bacteria in the presence of (a) **CID** and (b) **CSD** (1 – 128 µM).

| a) | b) |
| --- | --- |

Figure S57. The CFU percentage of bacteria in the presence of (a) **CIM** and (b) **CSM** (1 – 128 µM).

| a) | b) |
| --- | --- |

Figure S58. The CFU percentage of bacteria in the presence of (a) **DIB** and (b) **DSB** (1 – 128 µM).

| a) | b) |
| --- | --- |

Figure S59. The CFU percentage of bacteria in the presence of (a) **DID** and (b) **DSD** (1 – 128 µM).

| a) | b) |
| --- | --- |

Figure S60. The CFU percentage of bacteria in the presence of (a) **DIM** and (b) **DSM** (1 – 128 µM).

1. **Reference**

1. Brouwer, A. M., Standards for photoluminescence quantum yield measurements in solution (IUPAC Technical Report). *Pure and Applied Chemistry* **2011,** *83* (12), 2213-2228.

2. Halliwell, B.; Evans, P.; Whiteman, M., [35] Assessment of peroxynitrite scavengers in Vitro. *Methods in enzymology* **1999,** *301*, 333-342.

3. Shrivastava, A.; Gupta, V., Methods for the determination of limit of detection and limit of quantitation of the analytical methods. *Chronicles of Young Scientists* **2011,** (2), 21.

4. Beck, A. D., Density-functional thermochemistry. III. The role of exact exchange. *J. Chem. Phys* **1993,** *98* (7), 5648-6.

5. Lee, C.; Yang, W.; Parr, R. G. J. P. r. B., Development of the Colle-Salvetti correlation-energy formula into a functional of the electron density. *Physical review B* **1988,** *37* (2), 785.

6. Adamo, C.; Jacquemin, D., The calculations of excited-state properties with Time-Dependent Density Functional Theory. *Chem. Soc. Rev.* **2013,** *42* (3), 845-856.

7. Martin, R. L., Natural transition orbitals. *The Journal of chemical physics* **2003,** *118* (11), 4775-4777.
